# Integrative Analysis of Elicitor-Induced Camptothecin Biosynthesis in *Camptotheca acuminata* Plantlets Through a Combined Omics Approach

**DOI:** 10.3389/fpls.2022.851077

**Published:** 2022-03-24

**Authors:** Xiang Pu, Hu-Chuan Gao, Min-Ji Wang, Jia-Hua Zhang, Jia-Heng Shan, Meng-Han Chen, Li Zhang, Han-Guang Wang, An-Xiang Wen, Ying-Gang Luo, Qian-Ming Huang

**Affiliations:** ^1^College of Science, Sichuan Agricultural University, Ya’an, China; ^2^College of Life Science, Sichuan Agricultural University, Ya’an, China; ^3^Chengdu Institute of Biology, Chinese Academy of Sciences, Chengdu, China

**Keywords:** *Camptotheca acuminata*, abiotic elicitation, camptothecin biosynthesis, combined omics, bio-precursor

## Abstract

Treatments with abiotic elicitors can efficiently induce the accumulation of specialized metabolites in plants. We used a combined omics approach to analyze the elicitation effects of MeJa, AgNO_3_, and PEG on camptothecin (CPT) biosynthesis in *Camptotheca acuminata* plantlets. Untargeted analyses revealed that treatments with MeJa, AgNO_3_, and PEG significantly inhibited the photosynthetic pathway and promoted carbon metabolism and secondary metabolic pathways. The CPT levels increased by 78.6, 73.3, and 50.0% in the MeJa, AgNO_3_, and PEG treatment groups, respectively. Using *C. acuminata* plantlets after elicitation treatment, we mined and characterized 15 new alkaloids, 25 known CPT analogs and precursors, 9 iridoid biosynthetic precursors, and 15 tryptamine biosynthetic precursors based on their MS/MS fragmentation spectra. Using 32 characterized genes involved in CPT biosynthesis as bait, we mined 12 prioritized CYP450 genes from the 416 CYP450 candidates that had been identified based on co-expression analysis, conserved domain analysis, and their elicitation-associated upregulation patterns. This study provides a comprehensive perspective on CPT biosynthesis in *C. acuminata* plantlets after abiotic elicitation. The findings enable us to elucidate the previously unexplored CYP450-mediated oxidation steps for CPT biosynthesis.

## Introduction

The bio-manufacturing of camptothecin (CPT)—a plant-sourced anticancer alkaloid—depends on a complete understanding of its biosynthetic pathway. Since 1967, at least four different hypotheses have been proposed to describe the steps involved in the biosynthesis of CPT in *Camptotheca acuminata* (Wenkert et al., 1967; Winterfeldt, 1971; Hutchinson et al., 1974, 1979; Sadre et al., 2016; Pu et al., 2020). Over the last 55 years, 10 genes have been characterized based on the proposed biosynthetic pathways for CPT. These genes include *CaIPI1* and *CaIPI2* (Pan et al., 2008), *CaDXR* (Yao et al., 2008), *CaCPR* (Qu et al., 2015), *CaGES* (Chen et al., 2016), *CaIS* (Sadre et al., 2016), *CaGPPS* (Yang et al., 2017), *CaDL7H/CaSLAS1* and *CaDL7H/CaSLAS2* (Yang et al., 2019), *Ca10HGO* (Awadasseid et al., 2020) (involved in the iridoid pathway), *CaTDC* (López-Meyer and Nessler, 1997) (involved in generating tryptamine), *Ca10OMT* (Salim et al., 2018) (involved in the methyl modification of 10-hydroxycamptothecin), and 3 *CaSTR* (Yang et al., 2021) (responsible for the formation of strictosidine). However, the genes responsible for the downstream oxidation steps for CPT biosynthesis remain unknown. Recent studies have completed the transcriptomic and genomic sequencing of *C. acuminata* (Sun et al., 2011; Zhao et al., 2017; Kang et al., 2021). Based on these, thousands of unigenes and chromosome-scale genome assemblies are freely available for this plant. For each gene, dozens of homologous gene candidates can be mined from the genomic or transcriptomic datasets. Thus, the most challenging task in the post-genome era is the screening and characterizing the genes involved in CPT biosynthesis from numerous gene candidates.

**FIGURE 1:**
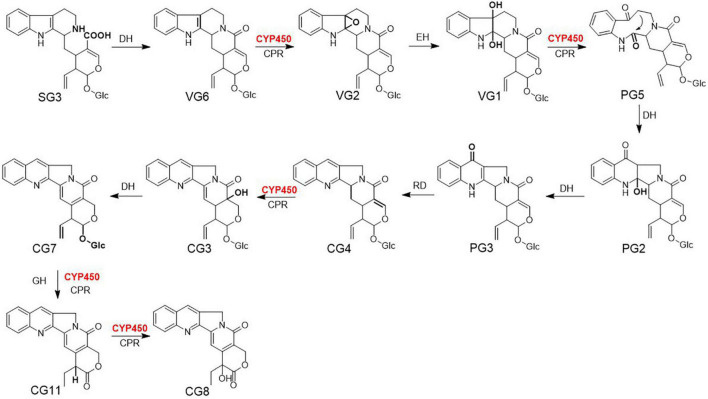
The downstream biosynthetic pathway for camptothecin (CPT) in *Camptotheca acuminata*. DH, dehydratase; CYP450, cytochrome P450; CPR, cytochrome P450 reductase; EH, epoxide hydrolase; RD, reductase; GH, glucose hydrolase; SG3, strictosidinic acid; VG6, strictosamide; VG2, strictosamide epoxide; VG1, strictosamide diol; PG5, strictosamide ketolactam; PG2, 2-hydroxypumiloside; PG3, pumiloside; CG4, deoxypumiloside; CG3, 16-hydroxy-15, 16-dihydrocamptothecoside; CG7, camptothecoside; CG11, 20-deoxycamptothecin; CG8, camptothecin. The catalytic site of each step is marked in bold.

Treatment with abiotic elicitors such as signal molecules, metal ions, drought stress, and salt stress is an efficient technique to induce the accumulation of specialized metabolites in plants (Ramakrishna and Ravishankar, 2011; Niazian and Sabbatini, 2021; Zhang et al., 2021). Recently, Burdziej et al. (2021) sprayed methyl jasmonate on *Vitis vinifera* cuttings to stimulate the production of defense metabolites, stilbenes and flavanols (). Drought and cold stress were proven to be an effective method to trigger flavone metabolism in *Poa crymophila Keng* (Wang et al., 2021). Zhang et al. (2018) verified that exposure to silver ions resulted in the upregulation of phenolic metabolites in *Cucumis sativus*. The genes involved in the biosynthesis of these specialized metabolites are likely synchronously upregulated after treatment with an abiotic elicitor. Thus, comparative omics analyses have served as an effective way to prioritize functional genes from numerous candidate genes. Strictosidinic acid and strictosamide have been identified as critical bio-precursors for CPT in *C. acuminata* (Sadre et al., 2016; Pu et al., 2020; Yang et al., 2021). The formation of strictosamide is followed by a sequence of enzymatically catalyzed reactions, including 2,7-epoxidation, epoxidation hydrolysis, dehydrogenation oxidation, intramolecular re-cyclization, 2-dehydration, 7-carbonyl reduction and dehydration, 16-hydroxylation and double-bond shift, glucosyl hydrolysis, 22-dehydrogenation oxidation, and 20-hydroxylation (Figure 1). The mining and characterization of CYP450 genes in *C. acuminata* is a critical step in elucidating the unidentified steps in CPT biosynthesis. In this study, we screened and utilized abiotic elicitors to induce CPT accumulation in *C. acuminata* plantlets and construct groups with different abiotic elicitors for comparative analyses. A combined omics approach was used to elucidate the response mechanism of CPT biosynthesis after elicitation. The results of these fundamental studies can be used to prioritize CYP450 gene candidates responsible for bio-catalyzing the downstream oxidation steps during CPT biosynthesis.

## Materials and Methods

### Seed Collection and Plantlets Culture

We collected mature seeds of *C. acuminata* at the beginning of November at Sichuan Agricultural University, Ya’an Campus. The seeds were air-dried, sealed in sample bags, and stored at 4°C. The seed coats were carefully removed before sterilization. The uncovered seeds were rinsed with 3% Triton X-100 for 3 min, washed with sterile water, soaked in 75% ethanol for 1 min, rinsed with 1% sodium hypochlorite solution for 3 min, and then washed with sterile water several times. The cleaned seeds were dried with sterile paper and transferred into tissue culture vessels (6–7 seeds/vessel) filled with half-strength MS medium (60 mL/vessel). The sterile seeds were cultivated at 25°C and 60% humidity in the dark until the cotyledons had germinated. The seedlings were then exposed to white light (photoperiod: 16 h/day). After germinating two real leaves (after ∼40 days), the plantlets were transferred to a different vessel for treatments with abiotic elicitors.

### Elicitation Treatments on *C. acuminata* Plantlets

Three elicitors—methyl jasmonate (MeJa), silver nitrate (AgNO_3_), and polyethylene glycol-20000 (PEG)—were dissolved in sterile water and filtered through a 0.22 μm filter to prepare sterile elicitation solutions. The autoclaved MS medium (60 mL/vessel) was cooled to 60°C, and different volumes of elicitor solutions were added and diluted to the respective target concentration (MeJa: 5–20 μM; PEG: 2.5–15 g/L; AgNO_3_: 5–75 μM). The 40-day old *C. acuminata* plantlets were transplanted into solidified MS medium for elicitor treatments (duration: 0–40 days) under sterile conditions. Equal volumes of sterile water were also added to the MS medium, and these plantlets were designated as the control (CK) group. All the *C. acuminata* plantlets were cultivated at 25°C and 60% humidity and exposed to white light (photoperiod: 16 h/day) throughout the treatment period. A single-factor experiment was introduced to optimize the elicitation conditions. The elicitor concentrations and treatment times for different elicitors are listed in Table 1. Six parallel treatments were conducted to minimize the differences between groups. The plantlets were collected after elicitor treatment and stored at −80°C.

**TABLE 1:** Treatment conditions for three abiotic elicitors.

Elicitor	Treatment concentration (C1–C4)	Treatment time (T0–T4)
MeJa	5, 10, 15, and 20 μM Treatment time: 10 days	0, 10, 20, 30, and 40 days Treatment concentration: 15 μM
PEG	2.5, 5.0, 10.0, and 15.0 g/L Treatment time: 10 days	0, 10, 20, 30, and 40 days Treatment concentration: 10 g/L
AgNO_3_	5, 25, 50, and 75 μM Treatment time: 10 days	0, 10, 20, 30, and 40 d Treatment concentration: 25 μM
CK	Sterile water	0, 10, 20, 30, and 40 days

*MeJa, AgNO_3_, and PEG. MeJa, methyl jasmonate; AgNO_3_, silver nitrate; PEG, polyethylene glycol-20000; CK, control group.*

### Growth Status Evaluation and Camptothecin Quantification

The growth status of each plantlet (leaf length, stem height, and root length) was determined immediately before cryogenic storage at −80°C. The statistical significance (*P*-value) of differences between groups was evaluated using univariate analysis (*t*-test). The frozen plantlets were air-dried at 25°C, ground to a fine powder, and used for extraction with a mixed solvent system (methanol-formic acid-H_2_O). The CPT content was determined using an Agilent 1290 Infinity UPLC system according to our previously developed method (Pu et al., 2020). In brief, a binary mobile phase (A: acetonitrile; B: H_2_O:0.1% HCOOH, 0.3 mL/min) was used. The gradient program was set as follows: 0–18 min, 5–50% (A); 18–25 min, 50–80% (A); 25–28 min, 80–98% (A); 28–30 min, 98–98% (A); and 30–32 min, 98–5% (A). A wavelength of 373 nm was used to quantify the CPT.

### Metabolite Extraction and LC-MS/MS Analysis

Fresh *C. acuminata* plantlets (0.1 g) from the control and elicitation groups were precooled with liquid nitrogen and thoroughly ground into fine powder. A 500 μL extraction solvent (CH_3_OH: H_2_O: HCOOH = 80:20:0.1) was used to re-suspend the homogenate. The extracts were chilled at 0°C for 10 min, carefully transferred, and filtered through centrifugation at 12,000 rpm. The supernatant was diluted with 150 μL distilled water. The diluents were rapidly filtered through a 0.22 μm filter in an Eppendorf tube via centrifugation at 12,000 rpm. The final sample filtrate was used for metabolomic analysis. In addition, an equal volume of the filtrate obtained from each sample was transferred into an Eppendorf tube and mixed by a vortex. This mixture was used for quality control, and the extraction solvent was designated as the blank sample. Sample analysis was performed on a Vanquish UHPLC system (Thermo Fisher, MA, United States) coupled with an Orbitrap Q Exactive™ HF-X mass spectrometer (Thermo Fisher). The sample solution was injected onto a Hyperil Gold C_18_ column (2.1 mm × 100 mm, 1.9 μm). The gradient program (phase A: 0.1% HCOOH; phase B: CH_3_OH; 0.0–1.5 min, 2–2% B; 1.5–12.0 min, 2–100% B; 12.0–14.0 min, 100–100%; 14.0–14.1 min, 100–2% B; 14.1–16.0 min, 2–2%; flow rate: 0.2 mL/min) was used for metabolite separation. The mass spectrometer parameters were as follows: positive mode; spray voltage: 3.2 kV; sheath gas flow rate: 35 arb; aux gas flow rate: 10 arb; capillary temperature: 320°C; collision energy: 20–60 eV.

### Multivariate Analysis, Metabolite Annotation, Differential Metabolites, and Enrichment Analysis

Peak alignment and quantitation of metabolites were performed using the Compound Discoverer 3.1 software. Retention time tolerance was set at 0.20 min. The actual mass tolerance was maintained at 5 ppm. The signal intensity tolerance was 30%, and the signal-to-noise ratio (S/N) was set at 3. The minimum intensity was assessed to be 100,000, and peak intensities were normalized and used to predict the molecular formula. The mzCloud and ChemSpider databases were used for alignment to generate qualitative and relative quantitative results. The statistical software R (version 3.4.3), Python (Python, version 2.7.6), and CentOS (CentOS, release 6.6) were used to perform the statistical analyses. The metabolites were annotated using the Kyoto Encyclopedia of Genes and Genomes (KEGG) database. The qualitative data matrices were exported and used for online multivariate analyses [principal component analysis (PCA) and partial least squares-discriminant analysis (PLS-DA)] and to identify discriminant metabolites using MetaboAnalyst 5.0 (Chong et al., 2019). The statistical significance (*P*-value) was evaluated using univariate analysis (*t*-test). The discriminant metabolites were screened according to their relative content variation (fold change ≥ 2 or ≤0.5, VIP > 2, *P*-value < 0.05). The enrichment of metabolic pathways was determined based on the ratio of the differential (MPDM) and background (MPBM) metabolites (N_MPDM_/N_DM_ > N_MPBM_/N_BM_). Metabolic pathways were designated as statistically significantly enriched based on the *P*-value (<0.05).

### Mining and Characterization of Camptothecin Analogs and Biosynthetic Precursors

The alkaloids were thoroughly mined and identified according to their typical cleavage patterns (Pu et al., 2020). In brief, the camptothecin group (CG) alkaloids were characterized according to the diagnostic fragment ion at *m/z* 168 and the specific cleavage (−56 Da or −70 Da). The pumiloside group (PG) alkaloids were characterized according to their diagnostic fragment ion at *m/z* 158 and the specific cleavage (−70 Da and −96 Da). The strictosidinic acid group (SG) alkaloids were determined according to the diagnostic fragment ion at *m/z* 144 and the typical cleavage (−17 and −70 Da). The vincosamide group alkaloids were determined according to the diagnostic fragment ion at *m/z* 144 and the specific cleavage (−70 and −96 Da). The vincosamide-camptothecin hybrid was characterized according to the diagnostic fragment ion at *m/z* 144 and the specific cleavage (−44 and −56 Da). Furthermore, the precursors involved in iridoid and tryptamine biosynthesis were mined and characterized according to their typical fragmentation patterns.

### Illumina Sequencing, Gene Annotation, Differential Analysis, and Pathway Enrichment Analysis

*Camptotheca acuminata* plantlets were collected from each sample group after elicitation treatment, immediately frozen in dry ice, and transported to Novogene Co., Ltd. (Beijing, China). Total RNA was extracted from the *C. acuminata* plantlets and purified using the Plant RNA Prep Kit (TIANGEN, Beijing, China) according to the manufacturer’s instructions. Sequencing libraries were prepared and sequenced on a Novaseq 6000 platform (Illumina, San Diego, CA, United States). The HISAT2 software (v2.0.5) (Kim et al., 2015) was used to generate paired-end reads and align them against the *C. acuminata* genome (Zhao et al., 2017). The StringTie software (v1.3.3b) was used to assemble the mapped reads and predict novel transcripts (Pertea et al., 2016). Gene expression levels were estimated based on FPKM values. The *DESeq2* package (1.16.1) in R was used to design a model based on the negative binomial distribution, which was used for comparative analyses between the elicitation and control groups (Robinson et al., 2010; Love et al., 2014). The resulting *P*-values were adjusted using the Benjamini–Hochberg method. Differential genes were identified according to the changes in their level of expression (| log_2_ fold change| > 0.0, adjusted *P*-value < 0.05). KEGG pathway enrichment analysis for differentially expressed genes was performed using the *ClusterProfiler* package in R (Kanehisa and Goto, 2000). Metabolic pathways were designated as statistically significantly enriched according to the *P*-value (< 0.05). Multivariate analyses (PCA) for genes were performed using MetaboAnalyst 5.0 (Chong et al., 2019).

### Co-expression Analysis

To screen the CYP450 candidates correlated with the characterized genes involved in CPT biosynthesis, we performed a weighted correlation network analysis using the gene expression data matrix, and the *WGCNA* package in R. The expression data were prefiltered based on the built-in threshold. The co-expression network was constructed using a soft threshold power of 8 and a mergeCutHeight parameter of 0.25. The Cytoscape 3.8.2 software was used to visualize the obtained network.

### qRT-PCR Verification

Total RNA was extracted from *C. acuminata* plantlets according to the methods described above, and traces of genomic DNA were removed with DNAase. Single-stranded cDNA was prepared using the PrimeScript™ Strand cDNA Synthesis Kit (TaKaRa, Kyoto, Japan). The TB Green™ Premix Ex Taq™ Kit (TaKaRa, Kyoto, Japan) was used to amplify the cDNA samples. qRT-PCR was performed on a CFX Connect™ real-time PCR system (Bio-Rad, Hercules, CA, United States) to evaluate gene expression levels. The primers used are listed in Supplementary Table 1. The relative expression level (2^–ΔΔCt^) was calculated using the 18S gene as the reference gene.

### Statistical Analysis

Excel 2019 was used to process the datasets and calculate the mean ± standard error (SE). Statistical significance analysis between the AgNO_3_, MeJa, PEG, and CK groups was performed using one-way ANOVA followed by Tukey’s test in SPSS 25.0 (IBM Corp., NY, United States). The Origin 8.5 software was used for graphical representations. Variables with different letters in the histograms represent significant differences between groups (*P* < 0.05).

## Results

### The Growth Status and Camptothecin Content of *C. acuminata* Plantlets After Elicitation Treatments

**FIGURE 2:**
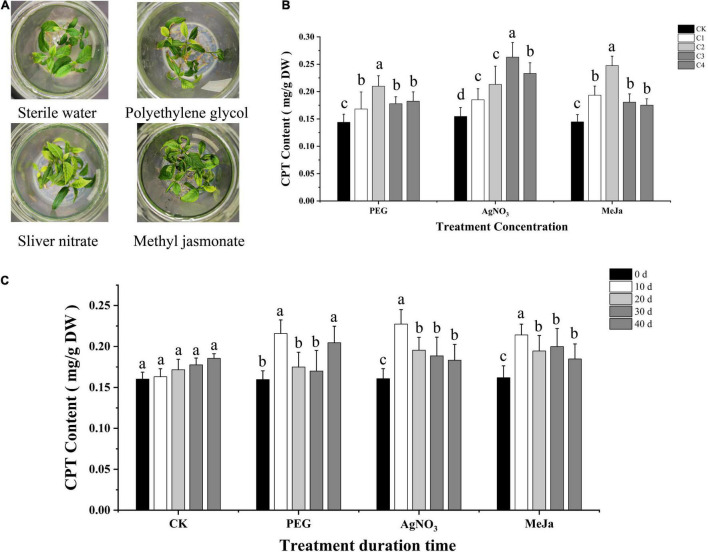
Optimization of elicitation conditions. **(A)** *C. acuminata* plantlets after elicitation treatments with PEG, AgNO_3_, and MeJa. **(B)** CPT contents (mg/g) under different treatment concentrations (C1– C4). **(C)** CPT contents (mg/g) at different treatment durations (0–40 days). Different letters (a, b, c, d) indicate significant differences at *P* < 0.05 (*t*-test). MeJa, methyl jasmonate; AgNO_3_, silver nitrate; PEG, polyethylene glycol-20000; CK, control group.

Compared with the control group (CK), the application of PEG (0–10 g/L) and AgNO_3_ (0–25 μM) slightly inhibited the growth of leaves and roots and reduced the stem height of *C. acuminata* plantlets. No significant inhibitory effect was observed after treatment with MeJa (0–15 μM) (Figure 2A). The leaves developed yellow spots at an elicitor (MeJa) concentration higher than 50 μM, and turned yellow when the concentration exceeded 1 mM. Severe dehydration was observed when the concentration of PEG was higher than 30 g/L. The *C. acuminata* plantlets exhibited withering when the concentration of AgNO_3_ exceeded 100 μM. The quantitative analysis of CPT levels indicated that the CPT levels were significantly altered 10 days after elicitation treatment (Figure 2C). Therefore, the elicitation time was fixed at 10 days for screening elicitor concentrations. The CPT content of *C. acuminata* plantlets was positively correlated with the elicitor concentration of PEG (2.5–15 g/L), AgNO_3_ (5–75 μM), and MeJa (5–20 μM). The CPT levels in the 5 g/L PEG, 50 μM AgNO_3_, and 10 μM MeJa treatment groups were 0.21, 0.26, and 0.25 mg/g dry weight, respectively. Compared with the CK group, elicitation treatment with 5 g/L PEG, 50 μM AgNO_3_, and 10 μM MeJa increased CPT levels by 50.0, 73.3, and 78.6%, respectively (*P* < 0.05) in Figure 2B. Therefore, the optimal elicitation conditions were 5 g/L PEG, 50 μM AgNO_3_, and 10 μM MeJa for 10 days, and these conditions were used for subsequent experiments.

### Untargeted Metabolomic Analysis, Metabolite Annotation, and Kyoto Encyclopedia of Genes and Genomes Enrichment Analysis

Based on the KEGG database, 1,139 metabolites were annotated and classified into 13 KEGG pathways (Supplementary Table 2). All metabolites annotated in the positive and negative ion modes were used for multivariate analyses. No apparent separation was observed between the elicitation treatment and control groups in the PCA score plot (Supplementary Figures 1A,C). However, the PLS-DA score plot showed better discrimination between groups (Supplementary Figures 1B,D). This indicated that the application of AgNO_3_, MeJa, and PEG could effectively elicit alterations in the metabolite profiles of *C. acuminata* plantlets. A comparative analysis between the AgNO_3_ and CK groups showed that 117 metabolites were significantly enriched, whereas 44 metabolites decreased in the positive mode (VIP > 1, *P* < 0.05). The relative contents of 40 metabolites increased, and those of 69 metabolites decreased in the negative mode (VIP > 1, *P* < 0.05). KEGG enrichment analyses indicated that metabolites classified into cyanoamino acid metabolism were significantly accumulated (N_MPDM_/N_DM_ > N_MPBM_/N_BM_, *P* < 0.05) after treatment with AgNO_3_. In the MeJa treatment group, the relative levels of 154 metabolites increased significantly, whereas those of 83 metabolites decreased in the positive mode (VIP > 1, *P* < 0.05). In the negative mode, the relative levels of 66 metabolites increased, whereas those of 102 metabolites decreased (VIP > 1, *P* < 0.05). KEGG enrichment analysis indicated that metabolites classified into plant hormone signal transduction and pyrimidine metabolism (N_MPDM_/N_DM_ > N_MPBM_/N_BM_, *P* < 0.05) were enriched after treatment with MeJa. In the PEG treatment group, the relative levels of 165 metabolites increased significantly, whereas those of 137 metabolites decreased in the positive mode (VIP > 1, *P* < 0.05). In the negative mode, the relative levels of 114 metabolites increased, and those of 154 metabolites decreased after treatment with PEG (VIP > 1, *P* < 0.05). KEGG enrichment analysis indicated that metabolites classified into cyanoamino acid metabolism, inositol phosphate metabolism, and amino acid biosynthesis metabolism were enriched (N_MPDM_/N_DM_ > N_MPBM_/N_BM_, *P* < 0.05) after treatment with PEG. Furthermore, treatment with AgNO_3_, MeJa, and PEG elicited the accumulation of dozens of secondary metabolites. The discriminating metabolites (fold change ≥ 2 or ≤0.5, VIP > 2, *P* < 0.05) for the elicitation and CK groups were screened and are illustrated in Supplementary Figure 2. The annotation results for the discriminating metabolites are provided in Supplementary Table 3.

### Gene Annotation, Gene Expression Level, and Kyoto Encyclopedia of Genes and Genomes Enrichment Analysis

A total of 633,833,052 raw Illumina paired-end reads were obtained and deposited at NCBI (BioProject No. PRJNA704189) and the Sequence Read Archive (Accession Nos. SRR13822238, SRR13822241, and SRR13822242 for the AgNO_3_ treatment group; Accession Nos. SRR13822232, SRR13822233, and SRR13822234 for the MeJa treatment group, Accession Nos. SRR13822231, SRR13822239, and SRR13822240 for the PEG treatment group, Accession Nos. SRR13822235, SRR13822236, and SRR13822237 for the control group). In total, 606,492,252 clean reads (6.80–8.65 G/sample) were generated from the raw sequence data. The data quality parameters for each sample were calculated and are summarized in Table 2. Moreover, 31,825 genes were annotated through alignment against the reference genome, and 1,303 novel transcripts were predicted using StringTie and annotated (Supplementary Table 4).

**TABLE 2:** Summary of the Illumina sequencing results.

Sample	Raw reads	Clean reads	Clean bases	Error rate	Q20	Q30	GC (%)
CKT1	46906796	45363096	6.80G	0.03	97.89	93.89	43.71
CKT2	52676774	51123802	7.67G	0.03	97.98	94.08	43.73
CKT3	51666540	50341840	7.55G	0.02	98.07	94.21	44.36
AgNO_3_T1	60257722	57676142	8.65G	0.02	98.05	94.23	43.69
AgNO_3_T2	54849808	52688278	7.90G	0.03	98.06	94.19	43.47
AgNO_3_T3	54019096	52313516	7.85G	0.03	98.00	94.10	43.51
PEGT1	49965482	46796810	7.02G	0.03	97.95	94.01	43.62
PEGT2	49655744	45362250	6.80G	0.03	97.79	93.65	43.24
PEGT3	56183338	53142054	7.97G	0.03	97.94	93.98	43.32
MeJaT1	48817576	46595518	6.99G	0.03	97.96	94.01	43.96
MeJaT2	55835600	54491424	8.17G	0.03	98.01	94.15	43.55
MeJaT3	52998576	50597522	7.59G	0.03	97.72	93.46	43.65

*MeJa, methyl jasmonate; AgNO_3_, silver nitrate; PEG, polyethylene glycol-20000; CK, control group.*

**FIGURE 3:**
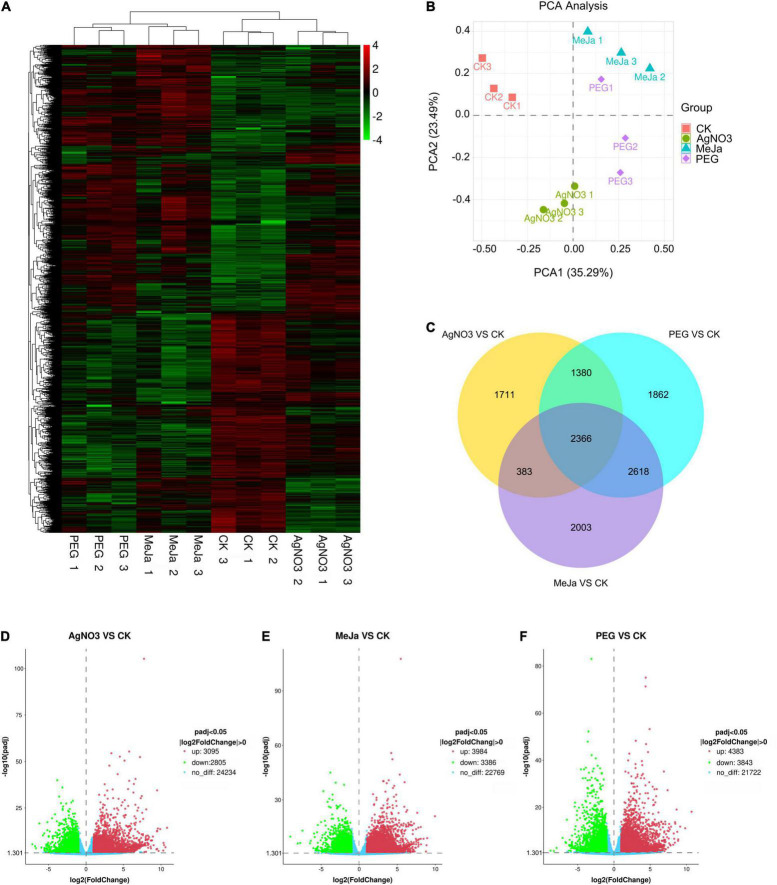
Comparative analysis at the transcriptomic level for the elicitation and control groups. **(A)** Cluster analyses for gene expression. **(B)** PCA score plot. **(C)** Venn diagram. Differential expression analyses: **(D)** AgNO_3_ vs. CK; **(E)** MeJa vs. CK; **(F)** PEG vs. CK. MeJa, methyl jasmonate; AgNO_3_, silver nitrate; PEG, polyethylene glycol-20000; CK, control group.

To evaluate the metabolic disturbance in *C. acuminata* plantlets at the transcriptomic level after treatment with PEG, AgNO_3_, and MeJa, whole transcripts were selected for gene expression cluster analyses and PCA analyses based on gene expression levels (Supplementary Table 5). No significant differences in expression level were observed for β-actin between the groups. The cluster analyses (Figure 3A) and PCA score plots (Figure 3B) indicated that the gene expression patterns of the PEG, AgNO_3_ and MeJa treatment groups were significantly different from those of the CK group, suggesting that the elicitation treatments resulted in a significant disturbance of transcriptional activity. Differential expression analysis between the four groups indicated that treatment with AgNO_3_ upregulated and downregulated the expression of 3,095 and 2,805 genes, respectively (*P*_adj_ < 0.05, | log_2_ fold change| > 0.0) (Figure 3D); treatment with MeJa upregulated and downregulated the expression of 3,984 and 3,386 genes, respectively (*P*_adj_ < 0.05, | log_2_ fold change| > 0.0) (Figure 3E); and treatment with PEG upregulated and downregulated the expression of 4,383 and 3,843 genes, respectively (*P*_adj_ < 0.05, | log_2_ fold change| > 0.0) (Figure 3F). A total of 2,366 differentially expressed genes were common between the groups and are depicted in Figure 3C.

Kyoto Encyclopedia of Genes and Genomes pathway enrichment analyses (Table 3) indicated that 27 genes involved in the photosynthetic pathway were downregulated (*P* < 0.05) and that 37 genes involved in carbon metabolism were upregulated in the AgNO_3_ treatment group (*P* < 0.05). This indicated that treatment with AgNO_3_ significantly inhibited the photosynthetic pathway (ath00195) and promoted carbon metabolism (ath01200). In the MeJa treatment group, 25 genes involved in photosynthesis were downregulated (*P* < 0.05), whereas 49 genes involved in amino acid biosynthesis and 46 genes involved in carbon metabolism were upregulated (*P* < 0.05). This indicated that treatment with MeJa significantly inhibited the photosynthetic pathway and promoted carbon metabolism and amino acid biosynthesis (ath01230). In the PEG treatment group, 25 genes involved in the photosynthetic pathway were downregulated (*P* < 0.05), whereas 74 genes involved in the ribosome pathway, 65 genes involved in carbon metabolism, and 58 genes involved in amino acid biosynthesis were upregulated (*P* < 0.05). This indicated that treatment with PEG significantly inhibited the photosynthetic pathway and promoted the ribosome pathway (ath03010), carbon metabolism, and amino acid biosynthesis. The changes in expression levels of 11 known genes were verified through qRT-PCR, and the results are depicted in Supplementary Figure 3. A total of 16 downregulated genes involved in the formation of photosystem (I and II) and 4 downregulated genes involved in the photosynthetic electron transport process were common between the groups. This indicated that treatment with these elicitors inhibited the photosynthetic pathway through the same mechanism. A total of 41 upregulated genes involved in carbon metabolism were shared between the MeJa and PEG treatment groups. A total of 28 upregulated genes involved in carbon metabolism were common between the AgNO_3_ and PEG treatment groups. These genes are mainly responsible for the glycolysis, citric acid cycle, and pentose phosphate pathway. It indicated that PEG is the most effective elicitor to promote carbon metabolism.

**TABLE 3:** KEGG pathway enrichment analysis for differentially expressed genes (DEGs).

KEGG ID	Description	DEGs			*P*-value
		Up	Down	Total	
**AgNO_3_ vs. CK**					
ath00195	Photosynthesis	0	27	27	6.64E-11
ath00630	Glyoxylate and dicarboxylate metabolism	13	17	30	3.13E-08
ath00710	Carbon fixation in photosynthetic organisms	12	17	29	8.33E-08
ath01200	Carbon metabolism	37	34	71	1.93E-07
ath00196	Photosynthesis-antenna proteins	0	11	11	1.25E-05
ath00860	Porphyrin and chlorophyll metabolism	3	17	20	1.09E-04
ath00910	Nitrogen metabolism	2	9	11	1.50E-03
**MeJa vs. CK**					
ath00195	Photosynthesis	0	25	25	2.72E-07
ath00710	Carbon fixation in photosynthetic organisms	13	18	31	3.64E-07
ath01200	Carbon metabolism	46	32	78	1.58E-06
ath04626	Plant-pathogen interaction	33	10	43	2.02E-04
ath01230	Biosynthesis of amino acids	49	16	65	5.97E-04
ath00630	Glyoxylate and dicarboxylate metabolism	9	16	25	8.86E-04
ath00260	Glycine, serine, and threonine metabolism	16	8	24	2.27E-03
ath00030	Pentose phosphate pathway	13	7	20	2.30E-03
**PEG vs. CK**					
ath03010	Ribosome	74	22	96	1.11E-09
ath01200	Carbon metabolism	65	26	91	3.47E-08
ath00195	Photosynthesis	1	25	26	9.81E-07
ath00020	Citrate cycle (TCA cycle)	24	1	25	6.90E-05
ath00710	Carbon fixation in photosynthetic organisms	15	14	29	1.11E-04
ath01230	Biosynthesis of amino acids	58	17	75	1.69E-04
ath00630	Glyoxylate and dicarboxylate metabolism	13	16	29	1.77E-04
ath00196	Photosynthesis-antenna proteins	0	10	10	2.23E-03
ath03050	Proteasome	22	1	23	3.85E-03

*MeJa, methyl jasmonate; AgNO_3_, silver nitrate; PEG, polyethylene glycol-20000; CK, control group.*

### Mining and Characterization of Camptothecin Analogs and Downstream Biosynthetic Precursors

To elucidate the metabolic mechanisms underlying the disruption of CPT biosynthesis in *C. acuminata* plantlets, we mined and characterized the alkaloids according to their fragmentation spectra in the positive mode. Ten CPT analogs (CG1–CG5, CG7–CG9, and CG11–CG12), four pumiloside analogs (PG2–PG5), three strictosidinic acid analogs (SG1–SG3), six vincosamide analogs (VG1–VG2 and VG4–VG7), and one vincosamide-camptothecin hybrid analog (VC5) were mined and identified according to their characteristic spectra (Pu et al., 2020). The [M + H]^+^ ion formula of CG14 (9.50 min, *m/z* 365.1137) was the same as that of CG5, and was determined to be C_20_H_17_N_2_O_5_. It was defined as a camptothecin analog based on the detection of the fragment (*m/z* 168.0690) and the characteristic cleavage of 44 Da (-CO_2_) and 56 Da (-C_3_H_4_O). However, compared with CG5, we observed a loss of 18 Da (-H_2_O) in the first step, except in the formation of the diagnostic ion. Therefore, CG14 was characterized as 5-hydroxycamptothecin.

We also identified 15 new alkaloids in the *C. acuminata* plantlets after elicitation treatment. The [M + H]^+^ ion formula of SG4 (7.91 min, *m/z* 371.1650) was annotated as C_20_H_23_N_2_O_5_, which was 16 Da higher than the fragment ion of SG3 (*m/z* 355.1641). SG4 was classified into the SG group based on the detection of a diagnostic ion at 144.0811, generated through the loss of 16 Da (-O), 70 Da (-C_4_H_6_O), 17 Da (-NH_3_), and 124 Da (-C_6_H_4_O_3_). A breakage of 16 Da was observed in the first cleavage step, except in the formation of the diagnostic ion. A characteristic loss of 17 Da was detected after the loss of 16 Da, except in the first step. Thus, the hydroxy group was determined on N4, except the benzene ring. SG4 was identified as a 4-hydroxystrictosidinic acid aglycone. The [M + H]^+^ ion formula of SG5 (8.19 min, *m/z* 535.2327) was defined as C_26_H_35_N_2_O_10_, which was 18 Da higher than that of SG3. Compared with the fragmentation spectrum of SG3, an extra loss of 18 Da (-H_2_O) was observed in the first step, and the fragmentation process of the intermediate fragment (*m/z* 517.2181) was the same as that of SG3. Thus, SG5 was determined to be 7-hydroxystrictosidinic acid. The [M + H]^+^ ion formula of VG8 (8.43 min, *m/z* 515.2002) was defined as C_26_H_31_N_2_O_9_, which was 18 Da higher than that of VG4. Two fragments at *m/z* 283.1076 and 265.0981 were generated through the characteristic loss of 70 Da from the intermediate fragments at *m/z* 353.1462 and 335.1406, respectively. The sequential breakup of 28 Da (-CO) and 28 Da (-CO)—apart from the specific cleavage of 96 Da—indicated a variation in ring D. Furthermore, we observed a diagnostic fragment at *m/z* 144.0816, and detected a loss of 18 Da (-H_2_O) in the initial step. VG8 was identified as 7-hydroxy-3,14-dehydrostrictosamide. The [M + H]^+^ ion formula of VG9 (9.13 min, *m/z* 353.1470) was determined to be C_20_H_21_N_2_O_4_, which was 162 Da lower than that of VG8. The fragmentation spectrum of VG9 was similar to that of the intermediate fragment of VG8 at *m/z* 353.1462. VG9 was determined to be the aglycone of VG8. The [M+H]^+^ ion formula of VG10 (9.21 min, *m/z* 677.2607) was defined as C_32_H_41_N_2_O_14_, which was 162 Da higher than those of VG2 and VG8. The sequential loss of 162 Da (-C_6_H_10_O_5_), 162 Da (-C_6_H_10_O_5_), -70 Da (-C_4_H_6_O), and -96 (-C_5_H_4_O_2_) indicated that it was a glucosyl derivative of strictosamide. Furthermore, the diagnostic fragment at *m/z* 144.0800 was generated by the breakage of 16 Da (-O). VG10 was determined to be 10-hydroxystrictosamide 6′-*O*-β-D-glucopyranoside. The [M + H]^+^ ion formula of VG11 (9.83 min, *m/z* 373.1847) was defined as C_20_H_25_N_2_O_5_. A diagnostic fragment at *m/z* 144.0819 and the specific cleavage of 70 Da from the ions at *m/z* 355.1292 and 337.1136 indicated that it belonged to the VG group. The existence of one hydroxy group on ring B and another one on ring E was verified according to the breakage of 18 Da (-H_2_O) and 18 Da (-H_2_O) in the first and second steps. VG11 was characterized as an aglycone of 7,21-dihydroxystrictosamide. The [M + H]^+^ ion formula of VG12 (10.02 min, *m/z* 515.2041) was determined to be C_26_H_31_N_2_O_9_, which was the same as that of VG8. It was classified as a strictosamide analog based on the diagnostic fragment at *m/z* 144.0801 and a specific breakup of 70 Da (-C_4_H_6_O). The cleavage of 162 Da (-C_6_H_10_O_5_) and 18 Da (-H_2_O) in the initial cleavage steps indicated the existence of glucosyl and hydroxy groups. In addition, the detection of a fragment at *m/z* 160.0760—which was 16 Da higher than the diagnostic ion—indicated that a hydroxy group was conserved in the diagnostic fragment. This hydroxy group was cleaved from the fragments at *m/z* 353.1483 and 283.1092 in the second or third cleavage step, respectively. Consequently, a hydroxy group was determined on the C2 of the B ring, except on the A ring. VG12 was characterized as an isomer of VG8 (2-hydroxy-6,7-dehydrostrictosamide). The [M + H]^+^ ion formula of VG13 (11.09 min, *m/z* 385.1762) was determined to be C_21_H_25_N_2_O_5_. It was classified as a strictosamide analog based on the detection of the diagnostic fragment at *m/z* 144.0815 and the cleavage of 70 Da (-C_4_H_6_O). The fragment at *m/z* 353.1472 was generated via the breakage of 32 Da (-CH_4_O) from the mother ion, and shared its cleavage pattern with the intermediate ion of VG3 at *m/z* 353.1517. Thus, VG13 was characterized as an aglycone of the 21-methoxy strictosamide epoxide.

**FIGURE 4:**
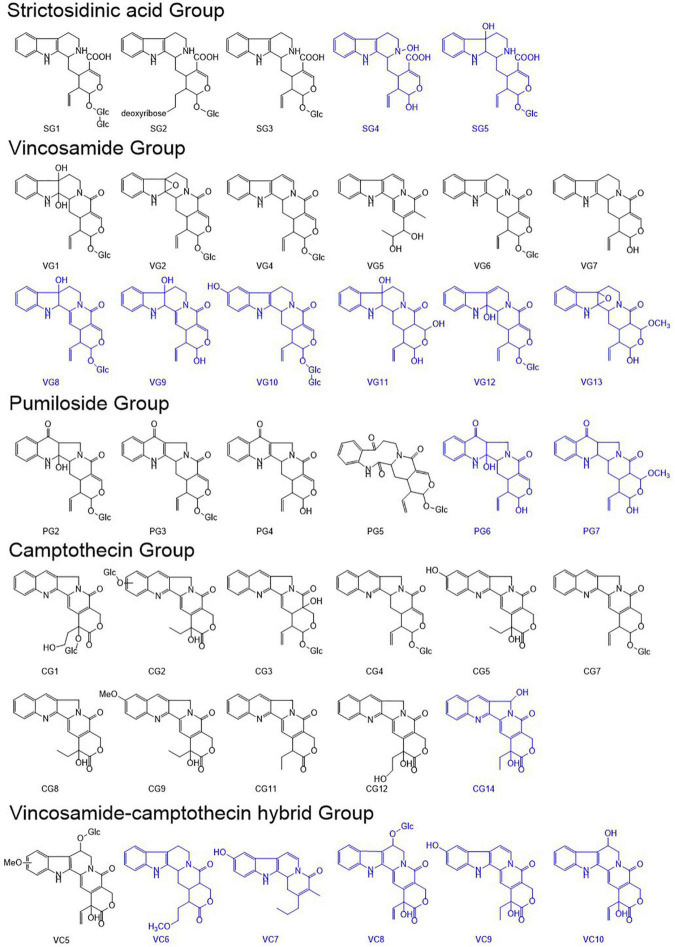
The structures of the identified camptothecin (CPT) analogs and the downstream biosynthetic precursors. New alkaloids are marked in blue.

The [M + H]^+^ ion formula of PG6 (10.98 min, *m/z* 369.2227) was determined to be C_20_H_21_N_2_O_5_, which is 162 Da lower than that of 2-hydroxypumiloside (PG2). The fragmentation process of PG6 was consistent with that of the intermediate fragment of PG2 at *m/z* 369.1434. Thus, PG6 was characterized as an aglycone of 2-hydroxypumiloside. The [M + H]^+^ ion formula of PG7 (10.04 min, *m/z* 383.2038) was determined to be C_21_H_23_N_2_O_5_. It was defined as a pumiloside analog based on the detection of the diagnostic fragment (*m/z* 158.0597) and the specific cleavage of 70 and 96 Da. The loss of 32 Da (-CH_4_O) in the first step or after the breakup of 18 Da (-H_2_O) indicated that a methoxy group was located on ring E. The fragment tree of the fragment at *m/z* 351.1341 was the same as that of PG4. Thus, PG7 was characterized as an aglycone of 21-methoxypumiloside. Interestingly, the [M + H]^+^ ion formula of VC6 (9.06 min, *m/z* 369.1922) was the same as that of PG6. However, this alkaloid was classified into the vincosamide-camptothecin hybrid group according to the existence of a diagnostic fragment at *m/z* 144.0818 and the breakage of 44 Da (-CO_2_) and 42 Da (-C_3_H_6_). The observed loss of methoxy (−32 Da) in the initial step and the cleavage of 42 Da—other than the loss of 56 Da in the third step—suggested the presence of a methoxy group on C18 and the absence of a hydroxy group on C20. Thus, VC6 was characterized as 18-methoxy-20-dehydroxystrictothecin. The [M + H]^+^ ion formula of VC7 (9.31 min, *m/z* 309.1581) was determined to be C_19_H_21_N_2_O_2_, which was 162 Da lower than that of VC4. Its fragmentation tree was the same as that of the intermediate ion (*m/z* 309.1606) of VC4. VC7 was characterized as an aglycone of VC4. The [M + H]^+^ ion formula of VC8 (9.44 min, *m/z* 527.1681) was determined to be C_26_H_27_N_2_O_10_, which was 30 Da lower than that of VC5. It also exhibited the same breakup pattern (162, 18, 44, and 28 Da) as that of VC5. Thus, VC8 was identified as 6-*O*-β-glucopyranosyl-18,19-dehydrostrictothecin. The [M + H]^+^ ion formula of VC9 (10.06 min, *m/z* 365.1137) was determined to be C_20_H_17_N_2_O_5_, and was the same as those of CG5 and CG14. It was defined as a VC analog and an isomer of CG5 according to the detection of the diagnostic fragment at *m/z* 144.0801 and the breakup of 44 and 56 Da. Specific cleavage of 16 Da (-O) was also detected in generating a fragment at *m/z* 221.0716. A hydroxy group on ring A was verified, and VC9 was identified as 10-hydroxystrictothecin. The [M + H]^+^ ion formula of VC10 (10.20 min, *m/z* 365.1134) was the same as that of VC9, and was determined to be C_20_H_17_N_2_O_5_. The same diagnostic ion and a typical cleavage pattern similar to that of VC9 were observed in this metabolite. However, a loss of 18 Da (-H_2_O) was observed in the first step, indicating the existence of a hydroxy group on ring C. VC10 was characterized as 6-hydroxy-18,19-dehydrostrictothecin. In summary, 40 alkaloids were identified in the *C. acuminata* plantlets after elicitation treatment, of which 15 were new alkaloids. The structures of these metabolites are illustrated in Figure 4, and their cleavage processes are shown in Supplementary Figure 4.

### Mining and Identifying the Biosynthetic Precursors Involved in the Iridoid Pathway

Nine iridoid biosynthetic precursors (IP1–IP9) were characterized based on their molecular weight and MS/MS fragmentation spectra. The [M + H]^+^ ion formula of IP1 (10.92 min, *m/z* 155.0856) was determined to be C_10_H_19_O. The formation of a series of intermediate ions at *m/z* 141.0140, 137.0957, 127.0344, 123.0028, and 109.1019 through the breakage of 14 Da (-CH_2_), 14 Da (-CH_2_), and 18 Da (-H_2_O) (in the same or a different order) indicated the existence of two methyl groups and one hydroxy group on the dialkene chain. Furthermore, a loss of 42 Da (-C_3_H_6_) or 56 Da (-C_3_H_4_O) was also detected before the loss of methyl or hydroxyl groups, indicating that the methyl or hydroxyl group was located at the end of the chain. Thus, IP1 was annotated as geraniol based on its fragmentation pattern (Tejedor et al., 2009). IP2 (7.71 min, *m/z* 171.0919) was identified as the hydroxy derivative of IP1 (10-hydroxygeraniol) based on the breakup of 14 Da (-CH_2_), 14 Da (-CH_2_), 18 Da (-H_2_O), and an additional loss of 18 Da (-H_2_O). The [M + H]^+^ ion formula of IP3 (7.12 min, *m/z* 167.0701) was determined to be C_10_H_15_O_2_, which was 4 Da lower than that of IP2. The generation of a product fragment at *m/z* 97.0653 via the continuous cleavage of 14 Da (-CH_2_), 28 Da (-CO), and 28 Da (-CO) suggested the existence of two aldehydes in IP3. The cleavage of methyl and hydroxyl groups was also detected in this metabolite. Therefore, IP3 was characterized as 10-oxogeranial. Interestingly, the [M-H]^–^ ion formula of IP4 (0.50 min, *m/z* 167.0337) was the same as that of IP3. A series of intermediate ions—the same as those obtained in IP3—were produced through the stepwise cleavage of 14 Da (-CH_2_), 14 Da (-CH_2_), and 18 Da (-H_2_O). Two specific intermediate fragments at *m/z* 151.0053 and 83.0500 were detected in IP4. The former was formed through the cleavage of 2 Da (-2H) from the fragment at *m/z* 153.0194, and the latter was formed through the stepwise loss of 54 Da (-C_3_H_2_O) via RDA and 14 Da (-CH_2_), which is the typical cleavage pattern for iridoids (Ling et al., 2014). Therefore, IP4 was identified as iridodial. The [M + H]^+^ ion formula of IP5 (6.71 min, *m/z* 183.1021) was determined to be C_10_H_15_O_3_. It was classified as an oxidation derivative of IP4 according to its molecular formula and cleavage pattern. In addition, a loss of 28 Da (-CO) was observed in the initial step and after the cleavage of methyl or hydroxy groups. IP5 was annotated as iridotrial, the oxidation derivative of iridodial. The [M-H]^–^ ion formula of IP6 (11.82 min, *m/z* 197.1185) was determined to be C_10_H_13_O_4_, which was 18 Da higher than that of IP5. A loss of 44 Da (-CO_2_) was detected in the first step or after the cleavage of 18 Da (-H_2_O), suggesting the existence of a carboxyl group on the hemiacetal ring. The specific cleavage of 2 Da (-2H) and 54 Da (-C_3_H_2_O) via RDA was also detected in forming a fragment at *m/z* 97.0653. Herein, IP6 was defined as 7-deoxyloganetic acid, a known biosynthetic precursor of iridoids. The [M + H]^+^ ion formula of IP7 (6.33 min, *m/z* 361.1391) was annotated as C_16_H_25_O_9_, which was 162 Da higher than that of IP6. IP7 was annotated as the glucosyl product of IP6 (7-deoxyloganic acid) based on the characteristic breakage of 162 Da (-C_6_H_10_O_5_) and 44 Da (-CO_2_). The [M-H]^–^ ion formula of IP8 (1.90 min, *m/z* 375.1273) was 16 Da higher than that of IP7 and was annotated as C_16_H_23_O_10_. Moreover, IP8 was classified as the hydroxy derivative of IP7. All the intermediate ions and the product fragment at *m/z* 113.0245 were formed through the stepwise cleavage of 162, 44, 2, and 54 Da. These fragments were 16 Da higher than that of IP7 in the positive mode, suggesting that a hydroxyl group was located on the cyclopentane ring. In this study, IP8 was annotated as loganic acid. The [M-H]^–^ ion formula of IP9 (2.96 min, *m/z* 373.1152) was 2 Da lower than that of IP8, and was annotated as C_16_H_21_O_10_. The loss of 162 Da (-C_6_H_10_O_5_), 44 Da (-CO_2_), and 18 Da (-H_2_O) suggested the existence of glucosyl, carboxyl, and hydroxy groups, which was the same as that in IP8. An additional loss of 28 Da (-CO) was detected in the formation of ions at *m/z* 183.0274, 139.0373, and 121.0653. Thus, the presence of an aldehyde group was confirmed. Hereafter, IP9 was characterized as secologanic acid, a critical precursor for CPT biosynthesis. In summary, we identified nine known precursors involved in the biosynthesis of the iridoid skeleton for CPT in *C. acuminata* plantlets after elicitation treatments. The structures of the nine iridoids and their detailed cleavage processes are shown in Supplementary Figure 4.

### Mining and Identifying the Biosynthetic Precursors Involved in the Tryptamine Pathway

We characterized 15 biosynthetic precursors (TP1–TP15) involved in the tryptamine pathway according to their molecular weight and typical fragmentation patterns. The [M-H]^–^ ion formula of TP1 (9.83 min, *m/z* 199.1334) was defined as C_4_H_8_O_7_P, which is the same as that of D-erythrose 4-phosphate, the initial biosynthetic precursor for tryptamine. The stepwise breakage of 28 Da (-CO), 18 Da (-H_2_O), and 18 Da (-H_2_O) verified the existence of a carbonyl and two hydroxy groups, which are the characteristic substituents of D-erythrose-4-phosphate (MassBank of north America (MoNA), 2022). A loss of 18 Da was also detected in the first step, and no other intermediate ions were observed. Therefore, TP1 was annotated as D-erythrose-4-phosphate. The [M-H]^–^ ion formula of TP2 (7.86 min, *m/z* 287.1046) was defined as C_7_H_12_O_10_P. The production of a fragment at *m/z* 207.1039 through the breakup of 80 Da (-HPO_3_) indicated the existence of a phosphate group. A sequential breakage of 44 Da (-CO_2_) and 18 Da (-H_2_O) (in the same or a different order) was also observed, which confirmed the presence of carboxyl and hydroxyl groups. The generation of a fragment at *m/z* 215.1195 through the breakage of 28 Da (-CO) after the loss of carboxyl verified the existence of a carbonyl group. The fragmentation pattern of TP2 was the same as that of 3-deoxy-D-arabinose-heptulosonate-7-phosphate, and TP2 was identified as such. The [M-H]^–^ ion formula of TP3 (3.23 min, *m/z* 189.0063) was defined as C_7_H_9_O_6_. We observed a stepwise cleavage of 44 Da (-CO_2_), 28 Da (-CO), and 18 Da (-H_2_O) (in the same or a different order) and a re-loss of 18 Da (-H_2_O) after the breakage of 44 and 18 Da. This suggested the existence of one carboxyl, one carbonyl, and two hydroxy groups. Therefore, TP3 was characterized as 3-dehydroquinic acid based on its cleavage pattern (MassBank of north America (MoNA), 2022). The [M-H]^–^ ion formula of TP4 (4.65 min, *m/z* 171.1019) was 18 Da lower than that of TP3, and was defined as C_7_H_7_O_5_. A similar stepwise cleavage of 44, 28, and 18 Da (in the same or a different order) was also observed. Thus, TP4 was characterized as a dehydration product of TP3 (3-dehydroshikimic acid). The [M-H]^–^ ion formula of TP5 (1.11 min, *m/z* 173.0086) was 2 Da higher than that of TP4, and was annotated as C_7_H_9_O_5_. The continuous breakage of 44 Da (-CO_2_), 18 Da (-H_2_O), and 18 Da (-H_2_O) indicated the presence of one carboxyl and two hydroxy groups. Compared with the MS/MS fragmentation spectrum of TP4, that of TP5 lacked the cleavage of 28 Da, and the TP5 had a molecular 2 Da higher than that of TP4. This indicated that TP5 was the hydrogenation derivative of IP4 (shikimate) (MassBank of north America (MoNA), 2022). The [M-H]^–^ ion formula of TP6 (10.01 min, *m/z* 253.1433) was 80 Da higher than that of TP5, and was annotated as C_7_H_10_O_8_P. The generation of a fragment at *m/z* 155.1077 via the breakage of 98 Da (-H_3_PO_4_) suggested the existence of an extra phosphate group compared with TP5. Breakages of 44 and 18 Da were also detected. Thus, TP6 was identified as 3-phosphonatoshikimate. The [M-H]^–^ ion formula of TP7 (12.96 min, *m/z* 323.1753) was designated as C_10_H_12_O_10_P. The existence of two carboxyl groups, one phosphate group, one hydroxy group, and an alkyl group was confirmed by the detection of the stepwise cleavage of 44 Da (-CO_2_), 44 Da (-CO_2_), 18 Da (-H_2_O), 80 Da (-HPO_3_), and 14 Da (-CH_2_). This is consistent with the fragmentation pattern of 5-O-(1-carboxy vinyl)-3-phosphoshikimate, and the structure of TP7 was determined accordingly. TP8 was identified as chorismic acid according to its [M-H]^–^ ion formula (9.14 min, *m/z* 225.1127), which was 98 Da lower than that of TP7. Similar to TP7, TP8 also exhibited two carboxyl groups, one hydroxy group, and an alkyl group. TP9 was annotated as anthranilate according to its [M + H]^+^ ion formula (8.75 min, *m/z* 138.0556) and fragmentation process (MassBank of north America (MoNA), 2022). The [M + H]^+^ ion formula of TP10 (14.76 min, *m/z* 350.2807) was determined to be C_12_H_17_NO_9_P. The breakage of 44 Da (-CO_2_) and 80 Da (-HPO_3_) suggested the existence of one carboxyl group and a phosphate group. The generation of a fragment at *m/z* 151.0962 via the loss of 90 Da (-C_3_H_6_O_3_) and 29 Da (-CHO) suggested the existence of a ribosyl group. Thus, TP10 was annotated as 5-phosphoribosyl anthranilate. Interestingly, a loss of 28 Da (-CO) was also observed in the first step. Thus, TP10 may also be defined as its isoform, TP11 (1-(2-carboxyphenylamino)-1-deoxy-D-ribulose 5-phosphate). The [M + H]^+^ ion formula of TP12 (12.43 min, *m/z* 288.1799) was the same as that of indole-3-glycerol phosphate, and was annotated as C_11_H_15_NO_6_P. The production of a fragment at *m/z* 116.0702 via the breakage of 142 Da (-C_2_H_7_O_5_P) and 30 Da (-CH_2_O) suggested the existence of an indole skeleton and a large side chain. Moreover, the formation of a fragment at *m/z* 130.0857 through the breakup of 98 Da (-H_3_PO_4_) suggested a phosphate group locates on the side chain of the indole skeleton. The loss of 18 Da (-H_2_O) and 28 Da (-CO) was also observed in the first and second steps, respectively. Thus, hydroxy and carbonyl groups may also locate on the side chain. The structure of the side chain was deduced according to the confirmed existence of phosphate, hydroxy, and carbonyl groups. Thus, TP12 was confirmed as indole-3-glycerol phosphate through minor revisions based on the total molecular weight (142 Da) of the side chain. TP13 was annotated as indole according to its [M + H]^+^ ion formula (MassBank of north America (MoNA), 2022). The [M + H]^+^ ion formula of TP14 (6.34 min, *m/z* 205.0119) was the same as that of tryptophan, and was determined to be C_11_H_13_N_2_O_2_. It was classified as an indole derivative based on detecting the characteristic fragment at *m/z* 118.0646. The cleavage of 44 Da (-CO_2_) and 17 Da (-NH_3_) was also observed, which suggested the existence of a carboxy and an amino group. Thus, TP14 was identified as a common biosynthetic precursor for the quinone alkaloid, tryptophan. The [M + H]^+^ ion formula of TP15 (6.54 min, *m/z* 161.1069) was 44 Da lower than that of tryptophan, and was determined to be C_10_H_13_N_2_. TP15 was determined to be tryptamine according to the detection of a characteristic loss of 17 Da (-NH_3_) and the diagnostic ion at *m/z* 117 (MassBank of north America (MoNA), 2022). The structures and fragmentation pathways of these precursors are shown in Supplementary Figure 4.

### Mapping of the Metabolic Pathways of Downstream Alkaloids and Mining of Previously Unidentified CYP450s

**FIGURE 5:**
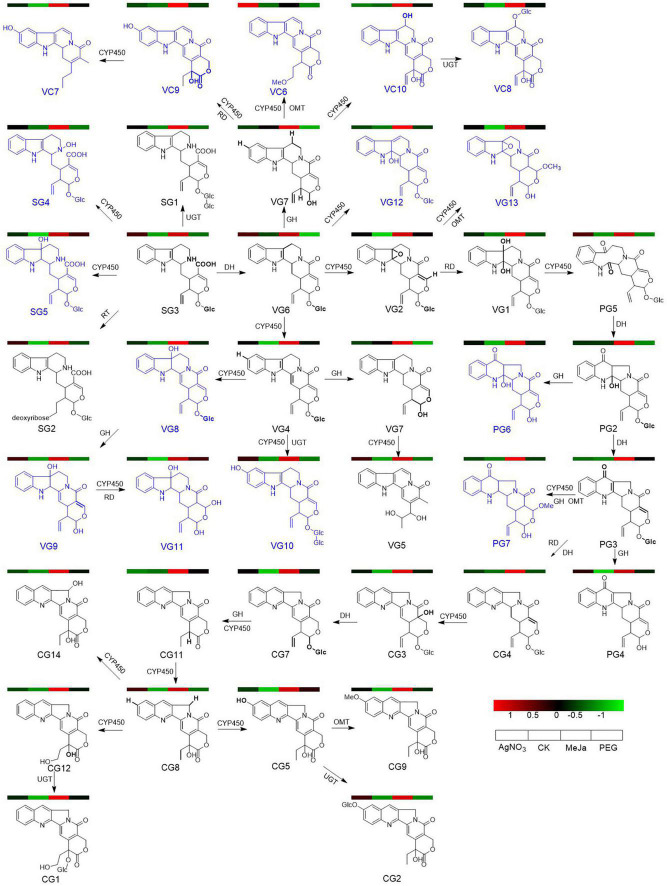
A detailed metabolic map of the 40 identified alkaloids in *C. acuminata* plantlets after elicitation treatment. New alkaloids are marked in blue, and their enrichment patterns are illustrated in the heat map. The catalytic site of each step is marked in bold. DH, dehydratase; CYP450, cytochrome P450; CPR, cytochrome P450 reductase; EH, epoxide hydrolase; RD, reductase; GH, glucose hydrolase; UGT, UDP-glucosyltransferase; OMT, O-methyltransferase.

**FIGURE 6:**
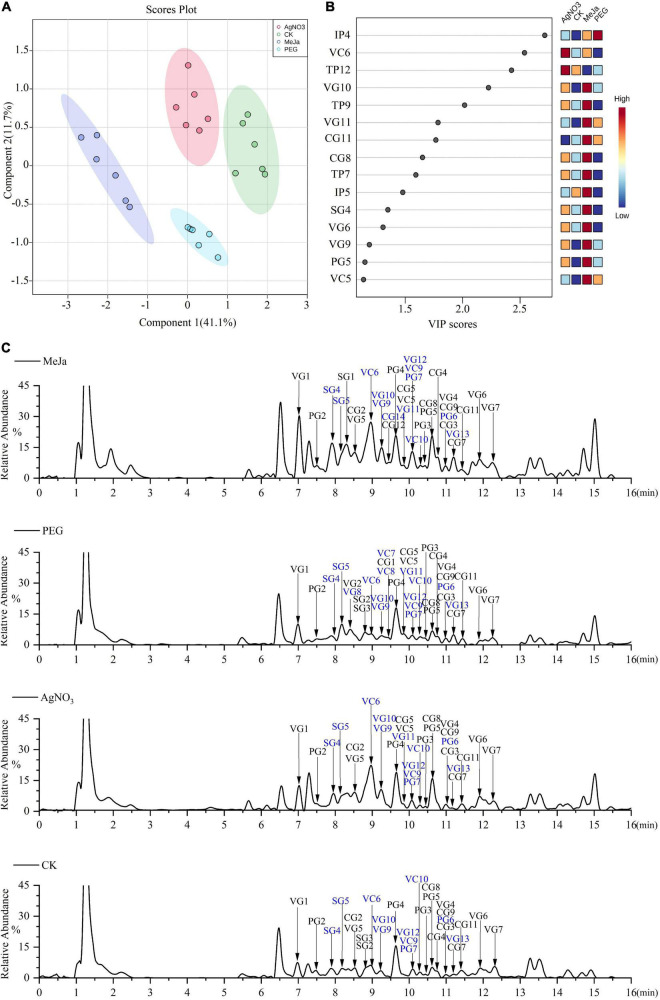
Comparative analysis at the metabonomic level for the elicitation and control groups. **(A)** PLS-DA score plot. **(B)** The discriminating metabolites. **(C)** Total ion chromatograms (TIC) in the positive mode for representative samples. New alkaloids are marked in blue. MeJa, methyl jasmonate; AgNO_3_, silver nitrate; PEG, polyethylene glycol-20000; CK, control group.

To elucidate the previously unexplored oxidation steps in the downstream pathways of CPT biosynthesis, we constructed a metabolic map for the 40 identified alkaloids (Figure 5) based on their biogenetic origin and chemical structures. The ion abundances of the 40 identified alkaloids in the elicitation and control groups were also extracted from the raw data and submitted to MetaboAnalyst 5.0 for multivariate analysis and enrichment analysis. Discrimination was obvious in the PLS-DA score plot (Figure 6A), indicating that the application of AgNO_3_, MeJa, and PEG elicited changes in the profiles of CPT analogs and bio-precursor in *C. acuminata* plantlets (Figure 6C). The discriminating metabolites (VIP > 2, *P* < 0.05) were screened (Figure 6B), and their enrichment patterns were visualized and integrated into the metabolic map for comparative analyses (Figure 5). Treatment with MeJa promoted alkaloid metabolism in *C. acuminata* plantlets. All the identified alkaloids, including the 12 critical downstream biosynthetic precursors (SG3–CG11), were significantly enriched, compared with the control group. In the AgNO_3_ and PEG treatment groups, most of the identified alkaloids and biosynthetic precursors were slightly upregulated. The upstream biosynthetic precursors of CPT—including IP1–IP5, IP8, IP9, TP1–TP9, TP12, TP14, and TP15—were enriched after treatment with AgNO_3_. In the MeJa treatment group, the relative levels of IP2–IP5, IP7–IP9, TP1–TP9, TP13, and TP15 were increased in this case. In the PEG treatment group, the relative levels of only IP2, IP4, TP1, and TP2 were enriched. In this study, MeJa and AgNO_3_ were identified as more effective elicitors for promoting CPT biosynthesis and diversification in *C. acuminata* plantlets. The expression levels of the unidentified genes involved in CPT biosynthesis and diversification would also likely be upregulated. These genes would likely co-express with the characterized genes in the elicitation groups.

Based on the current understanding of the CPT biogenetic pathway, 32 previously characterized genes involved in CPT biosynthesis were mined from the transcriptomic datasets according to the annotation results and their conserved domains. Their identities and coverages were calculated through sequence alignment with the characterized sequences, and the results are listed in Table 4. To further explore the genes responsible for the unexplored oxidation steps, we performed a co-expression analysis. In total, 33 modules were obtained for these genes, and the gene module dendrogram and network heatmap are displayed in Supplementary Figures 5, 6. Overall, the 32 aforementioned bait genes involved in CPT biosynthesis were clustered in only 12 modules (Table 4). Of these, 22 genes belong to the iridoid pathway, 5 to the tryptamine pathway, and 5 to the downstream pathways. Three modules, including the black, green, and turquoise modules, drew our attention and were further investigated. The genes adjacent to the unexplored pathway—including *Ca7-DLH/CaSLAS1*, *CaSTR2*, and *CaTDC2*—clustered only in black modules. *CaSTR1*, *CaSTR3*, *CaTSB*, *CaGES*, *CaIS*, and *CaIO*, clustered exclusively in green modules. Six genes involved in the upstream pathway—including *CaCMK*, *CaIPI2*, *CaG10H*, *Ca10HGO*, *CaCPR1*, and *CaCPR2*—clustered exclusively in the turquoise module. The clustering patterns of these bait genes indicated that the genes responsible for the unexplored pathway were most likely distributed in the green and black modules. Thus, the green and black modules could serve as gene reservoirs for fishing new genes responsible for the unidentified CYP450-mediated oxidation steps. All the CYP450 candidate genes correlated with these bait genes and distributed in the green and black modules were extracted from the co-expression matrix (Supplementary Table 6). As a result, 26 and 25 candidate genes annotated as CYP450 were obtained from the black and green modules, respectively. These were classified into 7 clans and 20 families according to their conserved domains.

**TABLE 4:** The characterized genes and the most probable CYP450 genes involved in camptothecin biosynthesis.

Description	Abbreviation	*C. acuminata* gene ID	GenBank number of the top-hit gene	Identity (%)	Coverage (%)	Module
Anthranilate synthase	ASA1	Cac_g008782	AAU84988.1	100.00	99	Tan
	ASA2	Cac_g018456	AAU84989.1	96.72	99	Tan
Tryptophan synthase	TSB	Cac_g001032	AAB97087.1	100.00	99	Green
Tryptophan decarboxylase	TDC1	Cac_g018974	AAB39708.1	97.41	99	Green-yellow
	TDC2	Cac_g023139	AAB39709.1	98.19	99	Black
1-deoxy-D-xylulose-5-phosphate synthase	DXS	Cac_g024944	DQ848672.1	77.38	91	Tan
1-deoxy-D-xylulose-5-phosphate reductoisomerase	DXR	Cac_g016318	ABC86579.1	88.16	99	Magenta
4-diphosphocytidyl-methylerythritol 2-phosphate synthase	CMS	Cac_g018722	FJ177510.1	77.15	81	Brown
4-diphosphocytidyl-2-C-methyl-D-erythritol kinase	CMK	Cac_g021688	DQ848671.1	76.90	98	Turquoise
2C-methyl-D-erythritol 2,4-cyclodiphosphate synthase	MECS	Cac_g008169	AF250236.1	72.65	99	Green-yellow
1-hydroxy-2-methyl-2-(*E*)-butenyl-4-diphosphate synthase	HDS	Cac_g022763	AY184810.2	88.65	99	Red
1-hydroxy-2-methyl-2-(*E*)-butenyl 4-diphosphate reductase	HDR	Cac_g014659	DQ864495.1	99.35	99	Midnight-bule
Isopentenyl diphosphate isomerase	IPI1	Cac_g008847	AAB94132.1	99.15	88	Grey60
	IPI2	Cac_g027591	AAB94133.1	100.00	99	Turquoise
Geranyl diphosphate synthase	GPPS	Cac_g026508	ATZ76916.1	80.57	68	Grey60
Geraniol synthase	GES	Cac_g014037	ALL56347.1	100.00	99	Green
Hydroxygeraniol oxidoreductase	GOR	Cac_g027560	KF302069.1	72.18	97	Magenta
Geraniol-10-hydroxylase	G10H	Cac_g027852	JF508378.1	94.61	96	Turquoise
10-hydroxygeraniol oxidoreductase	10HGO	Cac_g005530	AY342355.1	100	99	Turquoise
Iridoid synthase	IS	Cac_g006027	AON76722.1	100.00	99	Green
Iridoid oxidase	IO	Cac_g032709	KF302066.1	78.44	96	Green
UDP-glucose iridoid glucosyltransferase	7-DLGT	Cac_g008744	AB733667.1	77.11	93	Yellow
Loganic acid methyltransferase	LAMT	Cac_g005179	KF415116.1	53.91	96	Magenta
CYP72A565	7-DLH/CaSLAS1	Cac_g017137	QDC27812.1	100.00	99	Black
CYP72A610	7-DLH/CaSLAS2	Cac_g012666	QDC27813.1	99.62	99	Tan
NADPH-cytochrome P450 reductase	CaCPR1	Cac_g008486	AJW67229.1	99.15	99	Turquoise
	CaCPR2	Cac_g008487	KP162177.1	98.31	99	Turquoise
Strictosidine beta-D-glucosidase	CaSGD	Cac_g024440	AES93119.1	68.36	95	Purple
10-Hydroxycamptothecin *O*-methyltransferase	Ca10OMT	Cac_g014785	MG996006.1	92.90	99	Yellow
Strictosidine synthase	CaSTR1	Cac_g030447	QWX38542.1	100.00	99	Green
	CaSTR2	Cac_g030435	QWX38543.1	100.00	99	Black
	CaSTR3	Cac_g030441	QWX38544.1	100.00	99	Green
Oxidase	CYP71	Cac_g012391	PWA73981.1	70.00	99	Black
Oxidase	CYP71	Cac_g012484	OVA18686.1	66.03	93	Black
Oxidase	CYP71	Cac_g015559	RVW70431.1	67.83	96	Green
Oxidase	CYP71	Cac_g015564	RVW70431.1	66.30	89	Green
Oxidase	CYP72	Cac_g023088	BAX04012.1	63.53	98	Black
Oxidase	CYP72	Cac_g031955	BAX04010.1	71.94	95	Black
Oxidase	CYP72	Cac_g031956	BAX04010.1	70.14	95	Black
Oxidase	CYP81	Cac_g006015	QNS29972.1	70.53	99	Black
Oxidase	CYP81	Cac_g032235	QNS29988.1	59.26	98	Green
Oxidase	CYP81	Cac_g032228	OMO70595.1	71.43	66	Green
Oxidase	CYP81	Cac_g032236	QNS29988.1	59.49	98	Black
Oxidase	CYP81	Cac_g032246	QNS29936.1	60.95	93	Green

**FIGURE 7:**
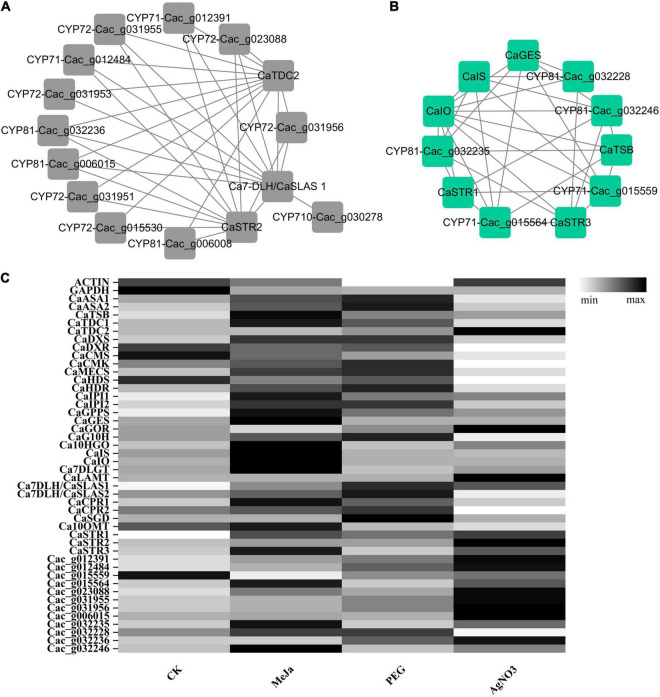
The co-expression network and expression patterns of the newly mined CYP450s and previously characterized genes in *C. acuminata*. **(A)** The co-expression network of targeted genes in the black module. **(B)** The co-expression network of targeted genes in the green module. **(C)** The expression patterns of genes in different treatment groups.

Of the aforementioned CYP450 candidates, 27 were classified into the CYP71 clan, which mostly contains hydroxylase or epoxidase. Among these, Cac_g012391, Cac_g012484, Cac_g015559, and Cac_g015564 belonged to the CYP71 family. Cac_g006008, Cac_g006015, Cac_g032236, Cac_g032228, Cac_g032235, and Cac_g032246 belonged to the CYP81 family. Most genes in the CYP71 and CYP81 families are usually involved in indole alkaloid, flavonoid, and terpenoid metabolism. This suggests that these genes probably function as epoxidase in the formation of VG2 from VG6, or as hydroxylase in the formation of CG5 from CG8 and VC9 from VG7. Seven CYP450 candidates—including Cac_023088, Cac_g031951, Cac_g031953, Cac_g031955, Cac_g031956, Cac_g023765, and Cac_g015530—were classified into the CYP72 clan, which is responsible for hydroxylation or oxidative C-C cleavage. These candidates are probably involved in modifying the iridoid part of the CPT precursor and its analogs during the formation of CG3, CG8, and VC9. Furthermore, Cac_g030278 was classified into the CYP710 family, which is normally involved in triterpenoid metabolism. It probably serves as an oxidative dehydrogenase during the formation of CG11. The remaining CYP450 candidates were classified into the CYP51, CYP74, CYP85, and CYP86 clans. These genes probably function as demethylase, epoxidase, successive oxidase, and hydroxylase, which are normally involved in sterol, fatty acid, diterpene, and triterpenoid metabolism in *C. acuminata*. These CYP450 candidates were further prioritized according to their co-expression patterns with the bait genes in the green and black modules. Interestingly, 12 of the 17 CYP71, CYP72, and CYP710 candidates obtained above were clustered in the black module. Only 7 of these—Cac_g012391, Cac_g012484, Cac_g031956, Cac_023088, Cac_g031955, Cac_g006015, and Cac_032236—showed good correlation with *Ca7-DLH/CaSLAS1, CaSTR2*, and *CaTDC2*. Four of these genes—Cac_g015559, Cac_g032246, Cac_g032235, and Cac_g015564—clustered in the green module and showed a strong correlation with *CaGES*, *CaIS*, *CaIO*, *CaSTR1*, *CaSTR3*, and *CaSGD*. Cac_g032228 showed a strong correlation with *CaGES*, *CaIS*, *CaIO*, *and CaSTR3*. The co-expression network for bait genes and the mined CYP450s in the black and green modules is illustrated in Figures 7A,B. We also extracted the full amino acid sequences of these CYP450 candidates from the genomic and transcriptomic datasets, and used these for sequence alignment analysis with the characterized genes. The identities and coverages of these newly mined CYP450s are listed in Table 4.

The expression levels of the newly mined CYP450 candidates and bait genes in the CK, PEG, AgNO_3_, and MeJa treatment groups were visualized (Figure 7C) according to their FPKM values. MeJa is a well-known plant hormone, and its application significantly enriched the expression of genes involved in the tryptamine and iridoid pathways. The expression levels of the characterized genes (*CaTDC1*, *CaIS*, *CaIO*, *Ca7DLGT*, *CaSTR1*, *CaSTR3*, and *Ca10OMT*) and the newly mined genes (Cac_g015564, Cac_g032228, Cac_g032235, and Cac_g032246) were significantly upregulated after treatment with MeJa. In contrast, in the AgNO_3_ treatment group, some of the genes involved in the tryptamine and iridoid pathways were downregulated compared with their levels in the CK group. However, the genes critical for CPT biosynthesis (*CaTDC2*, *CaSTR1*-*CaSTR3*, and 11 of the 12 newly mined CYP450 candidates, except for Cac_g032236) were noticeably enriched after treatment with AgNO_3_. In the PEG treatment group, most genes in the upstream pathway were upregulated. The expression levels of *CaSTR1*–*CaSTR3, CaTDC1*–*CaTDC2*, and the newly fished CYP450s were slightly influenced. Based on the transcriptomic and metabolic results described above, we can infer the reasons for their positive regulation of CPT levels in *C. acuminata*.

## Discussion

Following the release of the *C. acuminata* transcriptomic and genomic datasets, it has been theoretically possible to elucidate the entire biosynthetic pathway of CPT at the molecular level. Six genes involved in CPT biosynthesis have been found to belong to the iridoid pathway, and three *CaSTRs* have been mined from the genomic data and characterized recently (Qu et al., 2015; Chen et al., 2016; Sadre et al., 2016; Yang et al., 2017; Salim et al., 2018; Yang et al., 2019, 2021). However, the genes involved in the downstream pathway have remained unidentified, possibly due to two main reasons. First, the direct chemical evidence for the downstream pathway of CPT has been inadequate for a long time. Since 1967, at least four different hypotheses have been proposed to describe the downstream biogenetic steps for CPT biosynthesis in *C. acuminata*. Two early hypotheses for the biosynthesis of CPT were proposed based on the structures of corynanthidine and geissoschizine (Wenkert et al., 1967; Winterfeldt, 1971). The Gentianales-type biogenetic hypothesis was proposed based on labeled precursor feeding experiments in 1974 (Hutchinson et al., 1974, 1979). However, the genes responsible for the enzymatically catalyzed formation of strictosamide have remained undeciphered. Recently, a Cornales-type hypothesis was proposed by Sadre et al. (2016) and further replenished by Pu et al. (2020) based on their metabolomic analyses of mature *C. acuminata*. Based on these studies, a detailed downstream pathway for CPT biosynthesis was delineated, which could serve as a chemical guiding map for targeted gene mining. Second, screening and characterizing the genes in the downstream pathway for CPT biosynthesis from numerous homologous gene candidates is a challenging task. Dozens of homologous gene candidates might be annotated with the same function based on genomic or transcriptomic datasets, such as strictosidine synthase (Yang et al., 2021). Hundreds of CYP450 genes—which might be responsible for bio-catalyzing the unidentified steps, including epoxidation, dehydrogenation oxidation, and hydroxylation—have been annotated and are freely accessible. Therefore, strategically cloning and characterizing these candidate genes is in urgent need.

The accumulation patterns of CPT and its biosynthetic precursors should be highly correlated with the expression levels of genes responsible for their biosynthesis. Thus, the combined omics approach could serve as an efficient method for candidate gene screening. Taking the growth period, operability, and non-alkaloid constituent interference into consideration, we cultivated and utilized *C. acuminata* plantlets to construct groups with different elicitation treatments for comparative analyses. From ten known elicitors, we selected one phytohormone (MeJa), one metal ion (AgNO_3_), and one osmotic pressure regulator (PEG20000) to induce CPT biosynthesis in *C. acuminata* plantlets. Three types of treatments significantly inhibited the photosynthetic pathway (ath00195) and promoted carbon metabolism (ath01200) according to untargeted transcriptomic analyses. But the application of AgNO_3_, MeJa, and PEG significantly elicited the accumulation of CPT and dozens of secondary metabolites, as revealed by quantitative analyses and untargeted metabolomic studies. To focus on CPT biosynthesis, we thoroughly mined and identified CPT analogs and the biosynthetic precursors involved in the tryptamine, iridoid, and downstream pathways according to their characteristic cleavage patterns. Using *C. acuminata* plantlets after elicitation treatment, we mined and characterized 9 biosynthetic precursors (IP1–IP9) involved in the biosynthesis of secologanic acid, 15 precursors (TP1–TP15) involved in the biosynthesis of tryptamine, 15 new alkaloids, and 25 known CPT analogs and precursors. Full precursor coverage of the mainstream and branch metabolic pathway for CPT has achieved unprecedentedly in *C. acuminata* plantlets. These findings undoubtedly confirm the vigorous alkaloid metabolism of *C. acuminata* plantlets after elicitation. MeJa and AgNO_3_ were identified as the most effective elicitors for promoting CPT biosynthesis, as these elicitors enriched the relative levels of most of the downstream biosynthetic precursors.

The expression levels of the unidentified genes involved in CPT biosynthesis and diversification must also be upregulated after elicitation treatment. These genes are expected to co-express with the characterized genes. A total of 416 candidate genes were annotated as CYP450 in our transcriptomic dataset. Characterizing these CYP450 candidates one by one is time-consuming and unreasonable. Thus, using the characterized genes involved in CPT biosynthesis as bait, we performed co-expression analysis to screen CYP450 candidates. We found that the 32 bait genes were discretely distributed in 12 modules. Moreover, the genes adjacent to the unidentified steps—including *CaSTR1–CaSTR3*, *CaTDC2*, *Ca7-DLH/CaSLAS1*, *CaIS*, and *CaIO*—were clustered only in the black and green modules. Thus, 51 CYP450 candidate genes were extracted from the co-expression matrix of black and green modules and classified into 7 clans and 20 families based on their conserved domains and phylogeny. The diversification of alkaloid chemicals in CYP450 families has been intensively characterized in plants in recent years (Nguyen and Dang, 2021). Thus, the general function of each CYP450 candidate could be annotated according to its family category. Numerous studies have indicated that CYP71 and CYP81 usually function as hydroxylase or epoxidase and engage in modifying alkaloids or flavonoids. CYP72 is generally responsible for hydroxylation or C-C cleavage, and is involved in modifying iridoids. CYP710 has been described as an oxidative dehydrogenase. It is noteworthy that 10 CYP450 candidates were classified into the CYP71 and CYP81 families, 7 were classified into the CYP72 family, and one was classified into the CYP710 family. Only 12 of these CYP450 candidates showed a good correlation with the bait genes adjacent to the unexplored steps. Encouragingly, one of our gene candidates, Cac_032236, has been recently biochemically characterized as camptothecin 10-hydroxylase (Nguyen et al., 2021). This coincidentally verifies the accuracy of our mining strategy. The expression levels of these newly mined CYP450 candidates in the CK, PEG, AgNO_3_ and MeJa treatment groups were also visualized. The results indicated the underlying mechanism of elicitation of CPT enrichment after treatment with different elicitors. Treatment with MeJa triggers CPT production via upregulating most of the genes involved in CPT biosynthesis, while treatment with PEG and AgNO_3_ results in the upregulation of genes involved in the upstream and downstream pathway, respectively. Furthermore, our results demonstrate the technical feasibility of characterizing the CYP450 genes responsible for the epoxidation of VG6 to VG2, the dehydrogenation of CG7 to CG11, and the hydroxylation of CG8 to CG5, VG7 to VC9, CG4 to CG3, CG11 to CG8, and VG7 to VC3.

## Conclusion

The present work represents our second step in exploring the genes responsible for CPT biosynthesis. AgNO_3_, MeJa, and PEG were selected to induce CPT biosynthesis in *C. acuminata* plantlets for the first time. Untargeted analyses revealed that treatments with the three elicitors significantly inhibited the photosynthetic pathway and promoted carbon metabolism and secondary metabolic pathways. The CPT levels increased by 78.6, 73.3, and 50.0% in the MeJa, AgNO_3_, and PEG treatment groups, respectively. Moreover, we mined and characterized 15 new alkaloids and 25 known CPT analogs and downstream precursors according to their MS/MS fragmentation patterns. We also characterized 24 upstream biosynthetic precursors of CPT in *C. acuminata* plantlets. A transcriptomic approach was utilized to mine the CYP450 candidate genes potentially involved in the unexplored oxidation steps for CPT biosynthesis. We identified 416 CYP450 candidates in our transcriptomic dataset. Using 32 characterized genes involved in CPT biosynthesis as bait genes, we mined 12 prioritized CYP450 gene candidates through co-expression analysis, conserved domains analyses, and by examining their elicitation-associated upregulation patterns. These findings provide a comprehensive perspective of CPT biosynthesis in *C. acuminata* plantlets after abiotic elicitation and enable us to further elucidate the currently unknown CYP450-mediated oxidation steps for CPT biosynthesis.

## Data Availability Statement

The original contributions presented in the study are publicly available. This data can be found here: National Center for Biotechnology Information (NCBI) BioProject database under accession number PRJNA704189.

## Author Contributions

XP received grant support, designed the experiments, performed data analysis, and prepared and revised the manuscript. H-CG, J-HS, LZ, and M-HC prepared the RNA samples and performed the transcriptomic analysis. M-JW, H-CG, Y-GL, and XP mined and characterized the biosynthetic precursors. H-CG, J-HS, H-GW, A-XW, and Q-MH cultured the plantlets and performed elicitation treatments. All authors read and approved the final manuscript.

## Conflict of Interest

The authors declare that the research was conducted in the absence of any commercial or financial relationships that could be construed as a potential conflict of interest.

## Publisher’s Note

All claims expressed in this article are solely those of the authors and do not necessarily represent those of their affiliated organizations, or those of the publisher, the editors and the reviewers. Any product that may be evaluated in this article, or claim that may be made by its manufacturer, is not guaranteed or endorsed by the publisher.
