# Supplementary material for: Integrative Analysis of Elicitor-Induced Camptothecin Biosynthesis in Camptotheca acuminata Plantlets Through a Combined Omics Approach
Source: Front Plant Sci. 2022 Mar 24;13:851077. doi: 10.3389/fpls.2022.851077 (PMC8987726; doi:10.3389/fpls.2022.851077)
Supplement: Supplementary file 1 [file Data_Sheet_1.PDF]

Supplementary Material

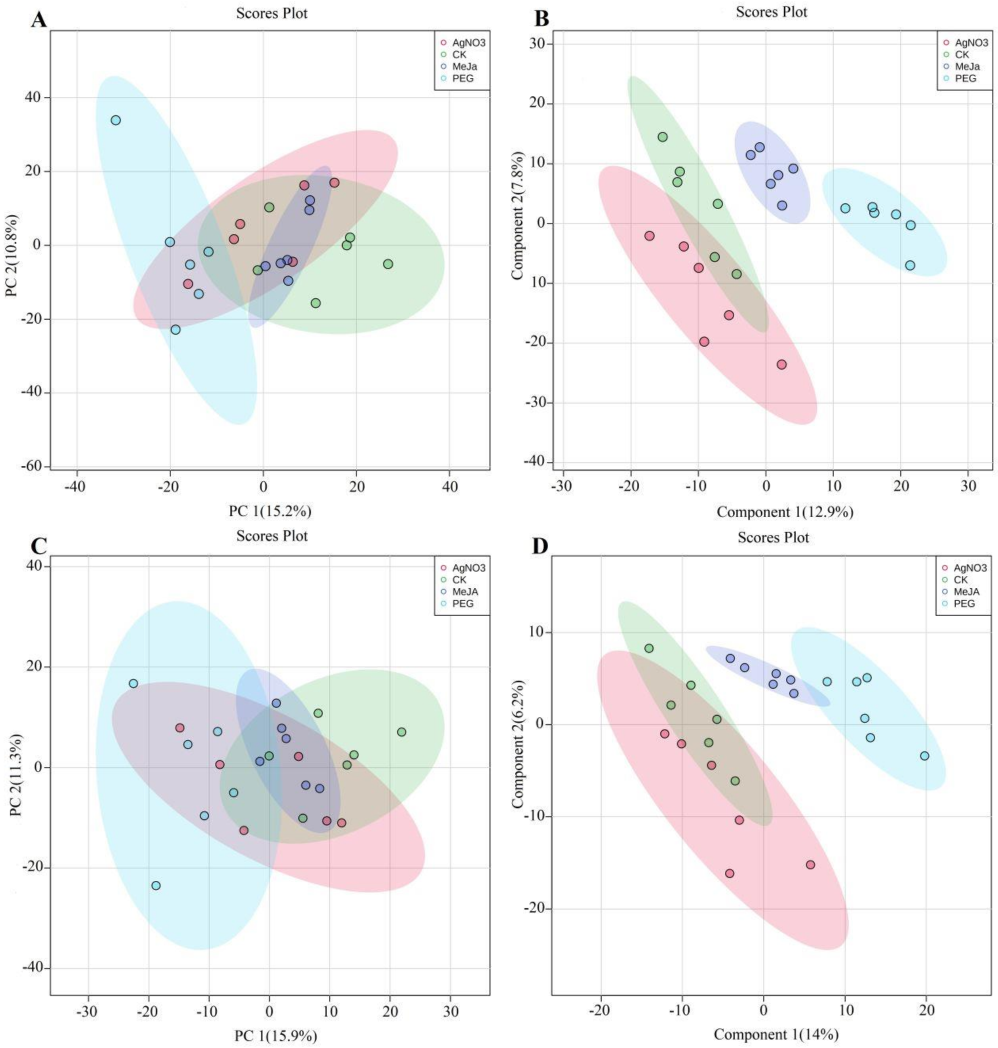

Supplementary Figure 1. Multivariate analysis for the elicitation and control groups. **(A)** PCA score plot for positive mode. **(B)** PLS-DA score plot for positive mode. **(C)** PCA score plot for negative mode. **(D)** PLS-DA score plot for negative mode. MeJa: methyl jasmonate; $AgNO_3$: silver nitrate; PEG: polyethylene glycol-20000; CK: control group.

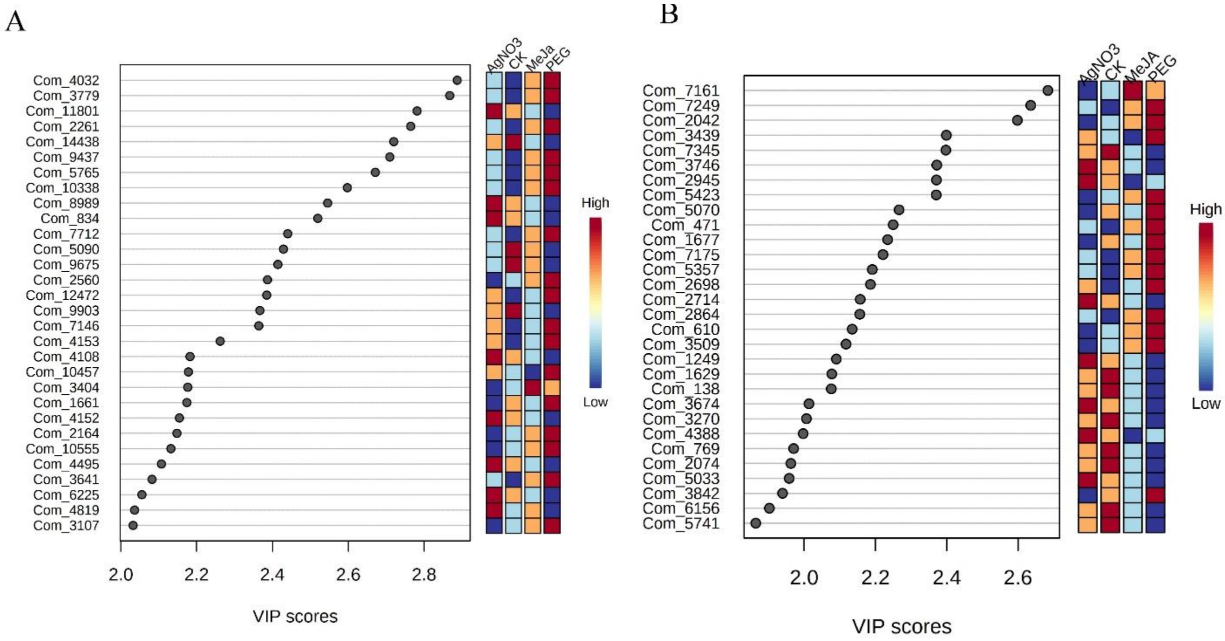

Supplementary Figure 2. Discriminating metabolites between the elicitation and control groups. **(A)** Discriminating metabolites for positive mode. **(B)** Discriminating metabolites for negative mode. MeJa: methyl jasmonate; $AgNO_3$: silver nitrate; PEG: polyethylene glycol-20000; CK: control group.

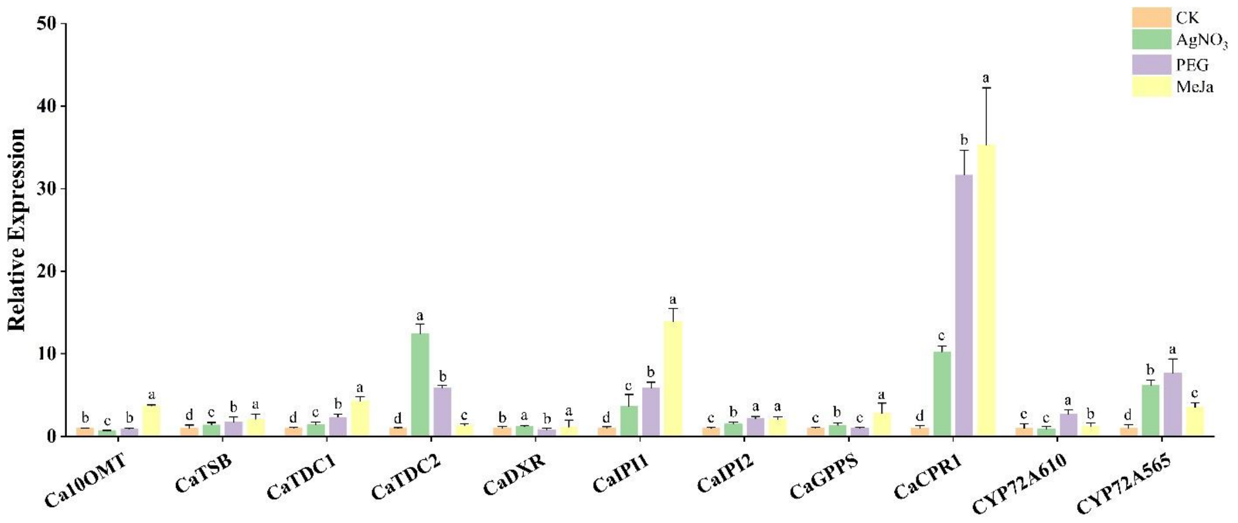

Supplementary Figure 3. qRT-PCR verification of differentially expressed genes.

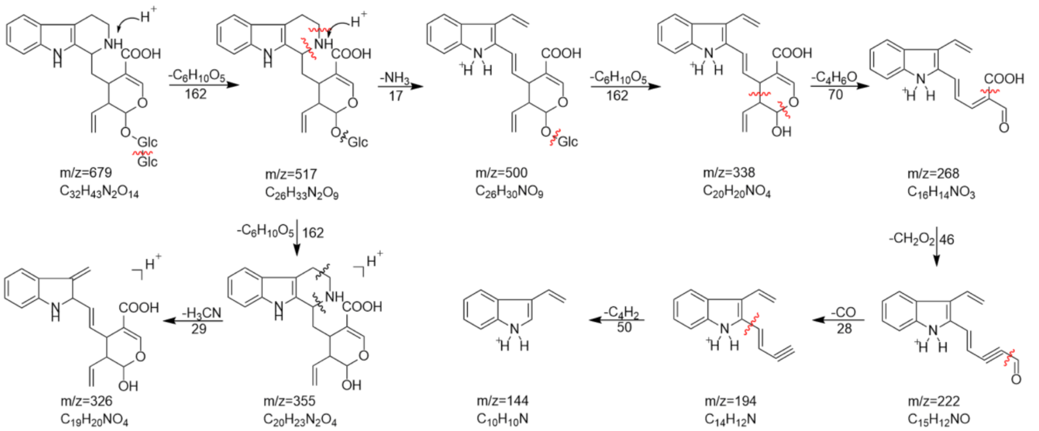

-C6H10O5 162
-NH3 17
-C6H10O5 162
-C4H6O 70
-CH2O2 46
-CO 28
-C4H2 50
-H3CN 29
m/z=679 C32H43N2O14
m/z=517 C26H33N2O9
m/z=500 C26H30NO9
m/z=338 C20H20NO4
m/z=268 C16H14NO3
m/z=326 C19H20NO4
m/z=355 C20H23N2O4
m/z=144 C10H10N
m/z=194 C14H12N
m/z=222 C15H12NO

SG1

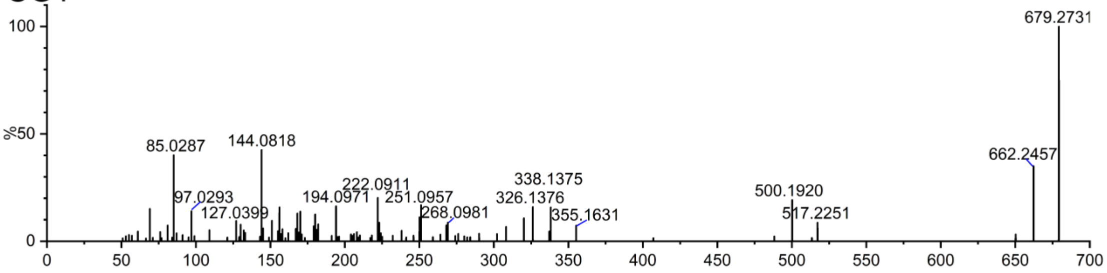

679.2731
662.2457
85.0287
144.0818
97.0293
127.0399
222.0911
194.0971
251.0957
268.0981
338.1375
326.1376
355.1631
500.1920
517.2251
%

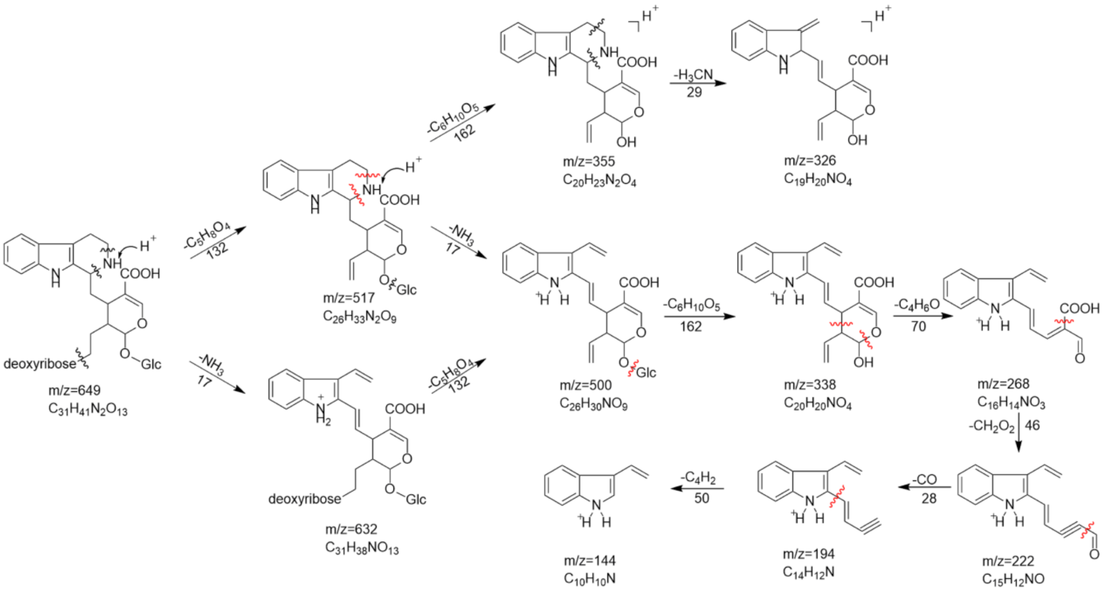

-C5H8O4 132
-NH3 17
-C6H10O5 162
-H3CN 29
-C4H6O 70
-CH2O2 46
-CO 28
-C4H2 50
deoxyribose
m/z=649 C31H41N2O13
m/z=517 C26H33N2O9
m/z=632 C31H38NO13
m/z=355 C20H23N2O4
m/z=326 C19H20NO4
m/z=500 C26H30NO9
m/z=338 C20H20NO4
m/z=268 C16H14NO3
m/z=222 C15H12NO
m/z=194 C14H12N
m/z=144 C10H10N

SG2

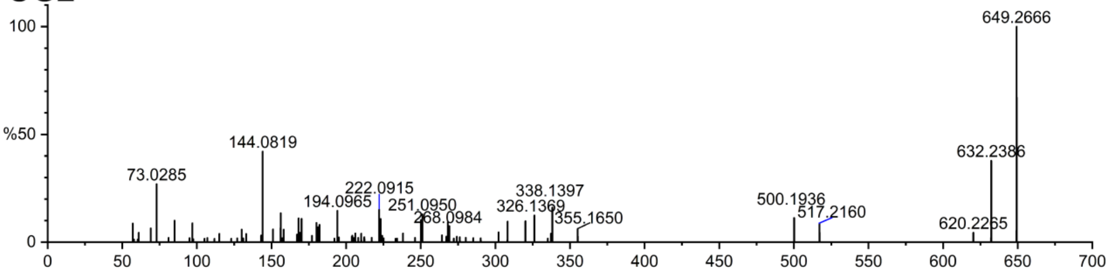

649.2666
632.2386
144.0819
73.0285
222.0915
194.0965
251.0950
268.0984
338.1397
326.1369
355.1650
500.1936
517.2160
620.2265
%

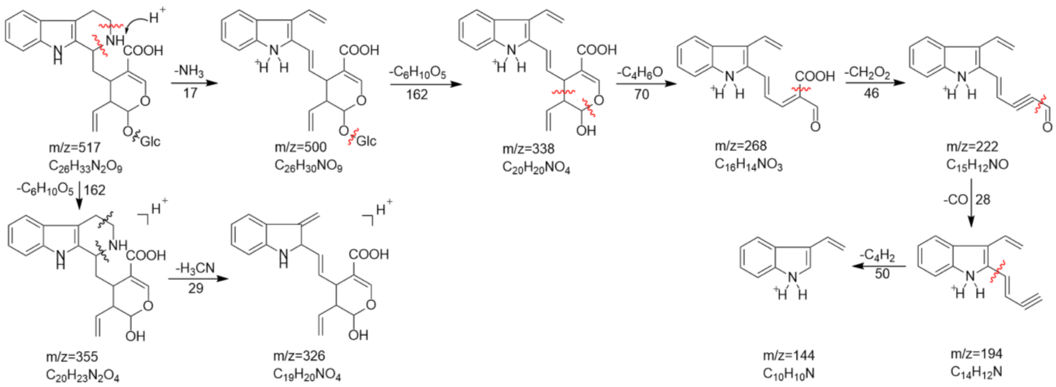
-NH3
17
-C6H10O5
162
-C4H6O
70
-CH2O2
46
m/z=517
C26H33N2O9
m/z=500
C26H30NO9
m/z=338
C20H20NO4
m/z=268
C16H14NO3
m/z=222
C15H12NO
-C6H10O5 162
-CO 28
-H3CN
29
-C4H2
50
m/z=355
C20H23N2O4
m/z=326
C19H20NO4
m/z=144
C10H10N
m/z=194
C14H12N

SG3

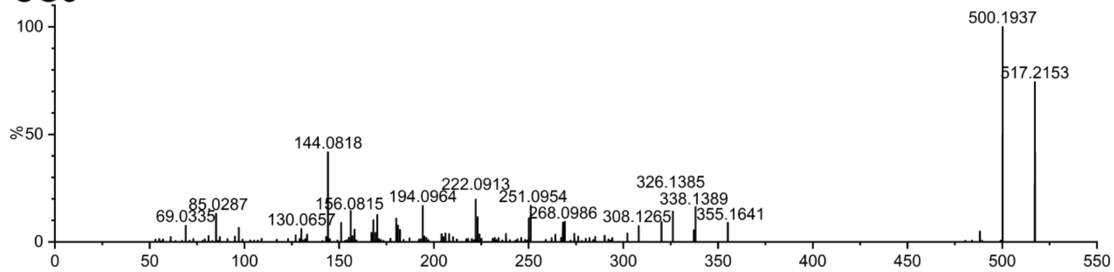
500.1937
517.2153
144.0818
222.0913
326.1385
194.0964
251.0954
338.1389
85.0287
156.0815
268.0986
308.1265
355.1641
69.0335
130.0657
%
m/z

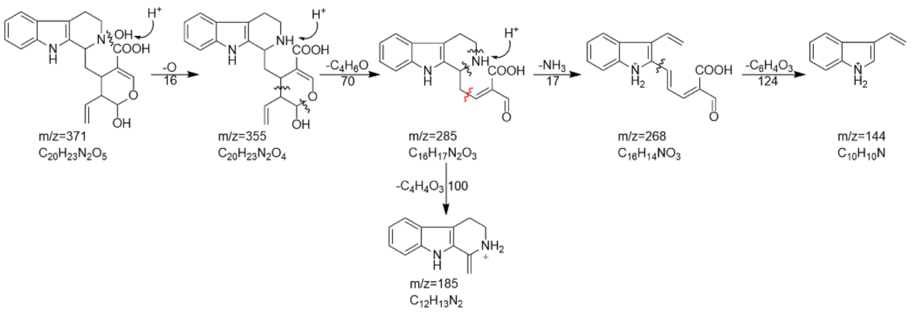
-O
16
-C4H6O
70
-NH3
17
-C6H4O3
124
m/z=371
C20H23N2O5
m/z=355
C20H23N2O4
m/z=285
C16H17N2O3
m/z=268
C16H14NO3
m/z=144
C10H10N
-C4H4O3 100
m/z=185
C12H13N2

SG4

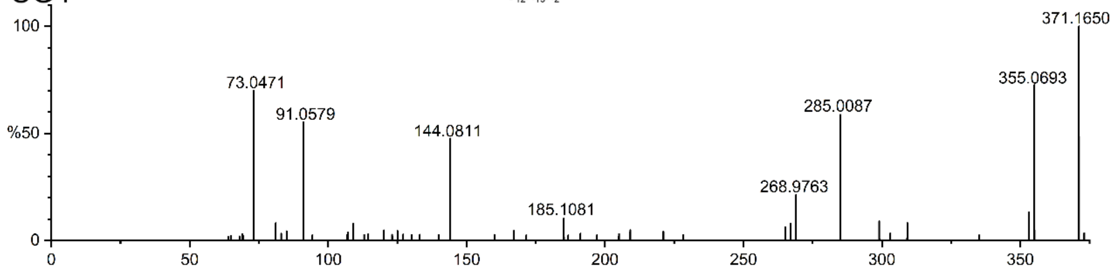
371.1650
73.0471
355.0693
285.0087
91.0579
144.0811
268.9763
185.1081
%

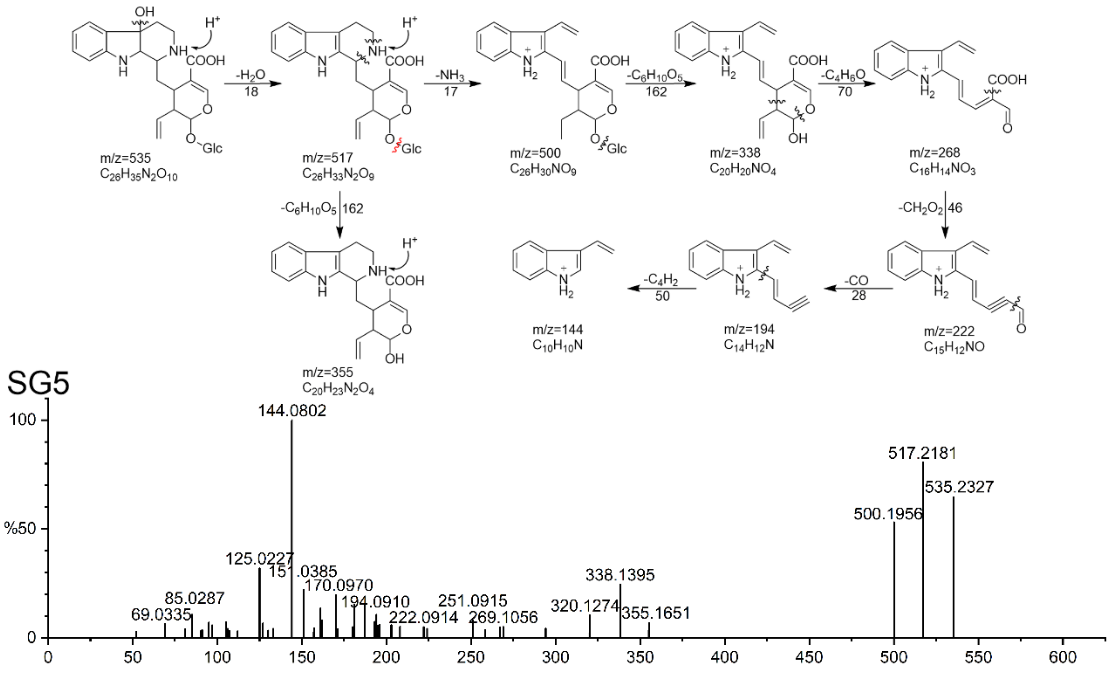

-H2O 18
-NH3 17
-C6H10O5 162
-C4H6O 70
-CH2O2 46
-CO 28
-C4H2 50
m/z=535 C26H35N2O10
m/z=517 C26H33N2O9
m/z=500 C26H30NO9
m/z=338 C20H20NO4
m/z=268 C16H14NO3
m/z=355 C20H23N2O4
m/z=222 C15H12NO
m/z=194 C14H12N
m/z=144 C10H10N
SG5
144.0802
517.2181
535.2327
500.1956
125.0227
151.0385
170.0970
194.0910
85.0287
69.0335
222.0914
251.0915
269.1056
320.1274
338.1395
355.1651
%50

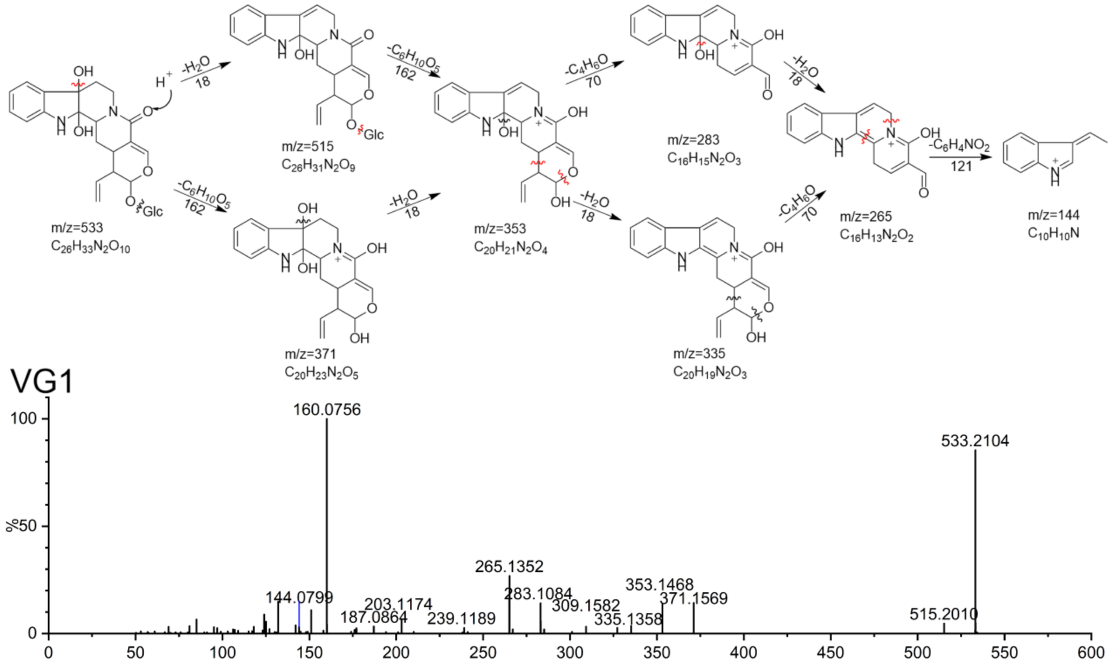

-H2O 18
-C6H10O5 162
-C4H6O 70
-C6H4NO2 121
m/z=533 C26H33N2O10
m/z=515 C26H31N2O9
m/z=371 C20H23N2O5
m/z=353 C20H21N2O4
m/z=283 C16H15N2O3
m/z=335 C20H19N2O3
m/z=265 C16H13N2O2
m/z=144 C10H10N
VG1
160.0756
533.2104
265.1352
283.1084
353.1468
371.1569
309.1582
335.1358
144.0799
203.1174
187.0864
239.1189
515.2010
%50

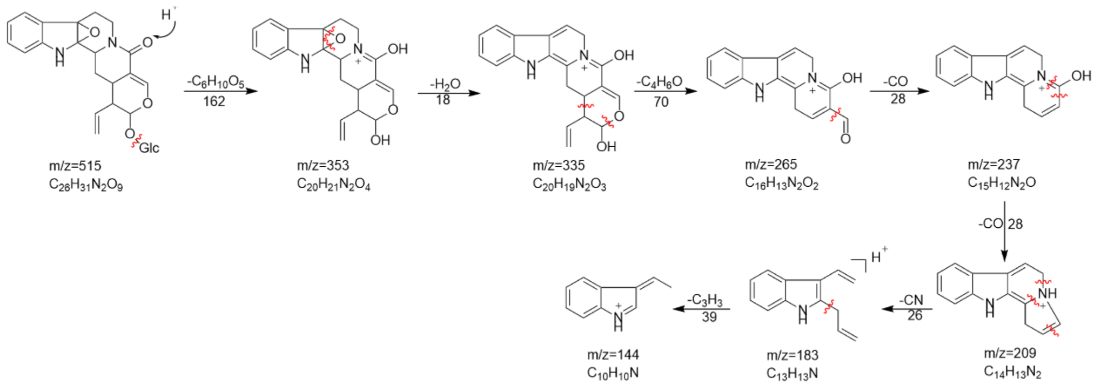
-$C_6H_{10}O_5$ 162
-H_2O 18
-C_4H_6O 70
-CO 28
-CO 28
-CN 26
-C_3H_3 39
m/z=515 $C_{26}H_{31}N_2O_9$
m/z=353 $C_{20}H_{21}N_2O_4$
m/z=335 $C_{20}H_{19}N_2O_3$
m/z=265 $C_{16}H_{13}N_2O_2$
m/z=237 $C_{15}H_{12}N_2O$
m/z=209 $C_{14}H_{13}N_2$
m/z=183 $C_{13}H_{13}N$
m/z=144 $C_{10}H_{10}N$

VG2

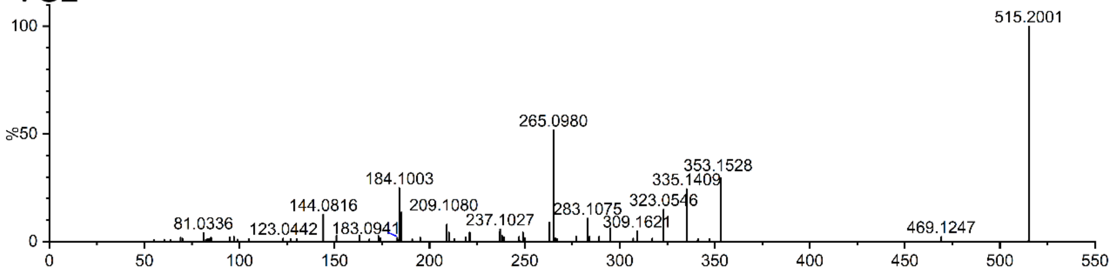
%
515.2001
265.0980
353.1528
335.1409
184.1003
323.0546
144.0816
209.1080
283.1075
81.0336
237.1027
309.1621
123.0442
183.0941
469.1247

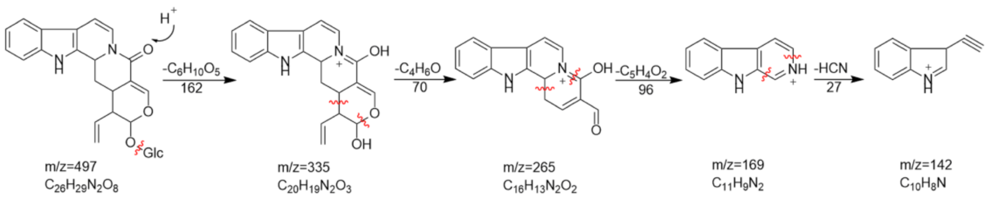
-$C_6H_{10}O_5$ 162
-C_4H_6O 70
-$C_5H_4O_2$ 96
-HCN 27
m/z=497 $C_{26}H_{29}N_2O_8$
m/z=335 $C_{20}H_{19}N_2O_3$
m/z=265 $C_{16}H_{13}N_2O_2$
m/z=169 $C_{11}H_9N_2$
m/z=142 $C_{10}H_8N$

VG4

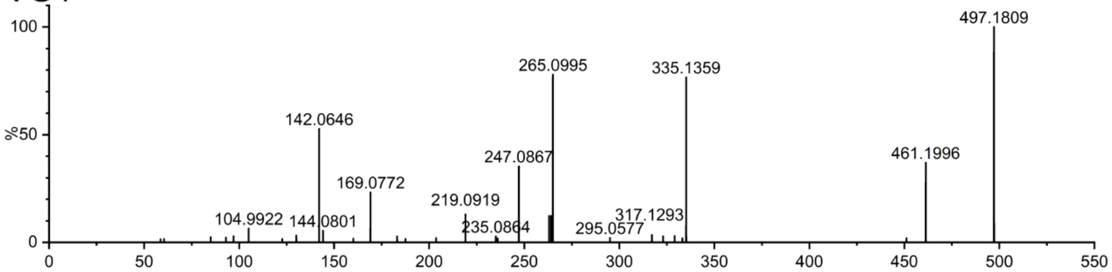
%
497.1809
265.0995
335.1359
142.0646
461.1996
247.0867
169.0772
219.0919
317.1293
104.9922
144.0801
235.0864
295.0577

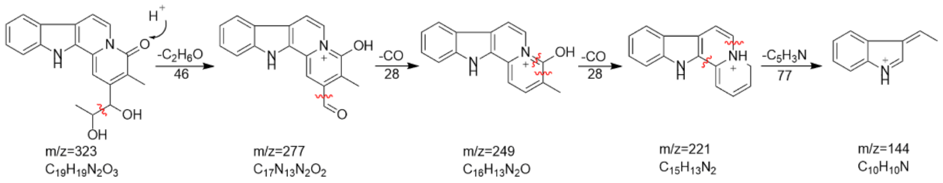
-C_2H_6O 46
-CO 28
-CO 28
-C_5H_3N 77
m/z=323 $C_{19}H_{19}N_2O_3$
m/z=277 $C_{17}N_{13}N_2O_2$
m/z=249 $C_{16}H_{13}N_2O$
m/z=221 $C_{15}H_{13}N_2$
m/z=144 $C_{10}H_{10}N$

VG5

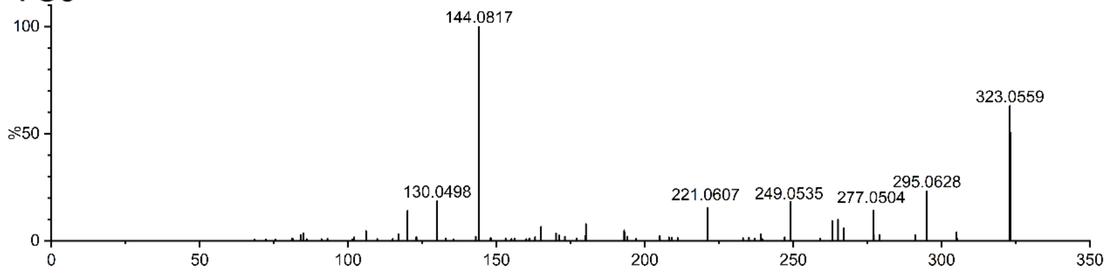
%
144.0817
323.0559
130.0498
221.0607
249.0535
295.0628
277.0504

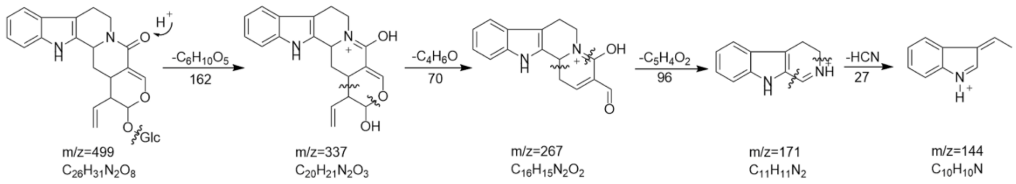

-C6H10O5
162
-C4H6O
70
-C5H4O2
96
-HCN
27
m/z=499
C26H31N2O8
m/z=337
C20H21N2O3
m/z=267
C16H15N2O2
m/z=171
C11H11N2
m/z=144
C10H10N

VG6

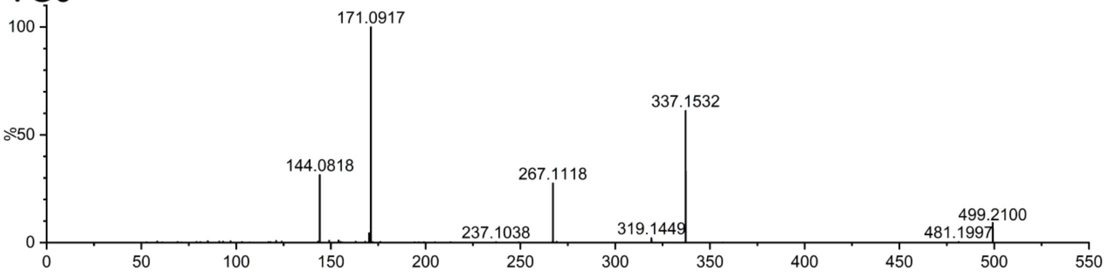

171.0917
337.1532
144.0818
267.1118
237.1038
319.1449
499.2100
481.1997
%
100
50
0
0
50
100
150
200
250
300
350
400
450
500
550

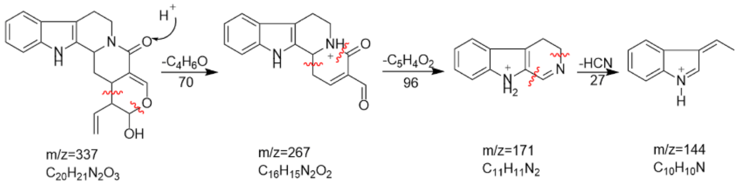

-C4H6O
70
-C5H4O2
96
-HCN
27
m/z=337
C20H21N2O3
m/z=267
C16H15N2O2
m/z=171
C11H11N2
m/z=144
C10H10N

VG7

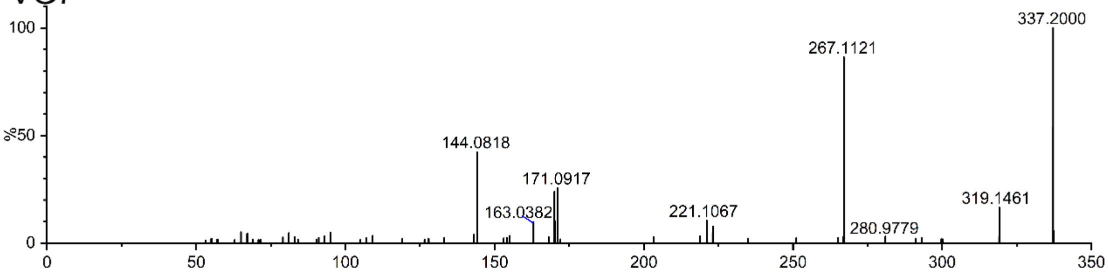

337.2000
267.1121
144.0818
171.0917
163.0382
221.1067
319.1461
280.9779
%
100
50
0
0
50
100
150
200
250
300
350

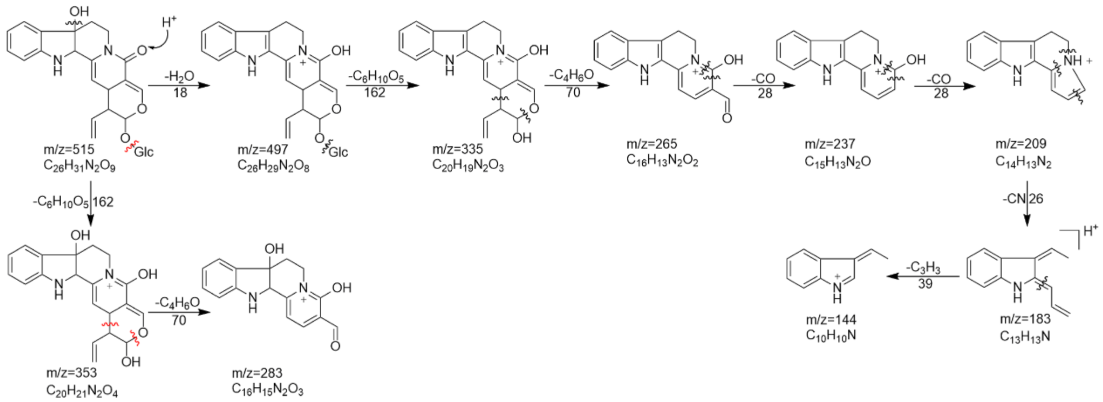

m/z=515
$C_{26}H_{31}N_2O_9$
-H_2O 18
m/z=497
$C_{26}H_{29}N_2O_8$
-$C_6H_{10}O_5$ 162
m/z=335
$C_{20}H_{19}N_2O_3$
-C_4H_6O 70
m/z=265
$C_{16}H_{13}N_2O_2$
-CO 28
m/z=237
$C_{15}H_{13}N_2O$
-CO 28
m/z=209
$C_{14}H_{13}N_2$
-CN 26
m/z=183
$C_{13}H_{13}N$
-C_3H_3 39
m/z=144
$C_{10}H_{10}N$
-$C_6H_{10}O_5$ 162
m/z=353
$C_{20}H_{21}N_2O_4$
-C_4H_6O 70
m/z=283
$C_{16}H_{15}N_2O_3$

VG8

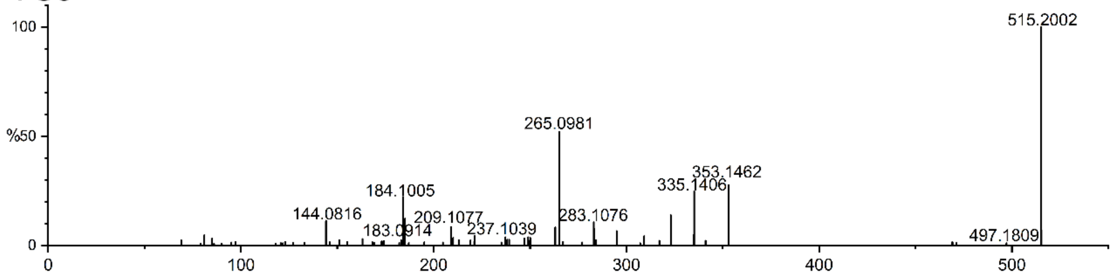

515.2002
265.0981
353.1462
335.1406
184.1005
144.0816
209.1077
183.0914
237.1039
283.1076
497.1809
%50

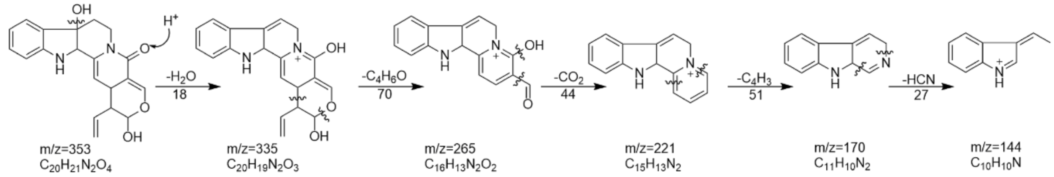

m/z=353
$C_{20}H_{21}N_2O_4$
-H_2O 18
m/z=335
$C_{20}H_{19}N_2O_3$
-C_4H_6O 70
m/z=265
$C_{16}H_{13}N_2O_2$
-CO_2 44
m/z=221
$C_{15}H_{13}N_2$
-C_4H_3 51
m/z=170
$C_{11}H_{10}N_2$
-HCN 27
m/z=144
$C_{10}H_{10}N$

VG9

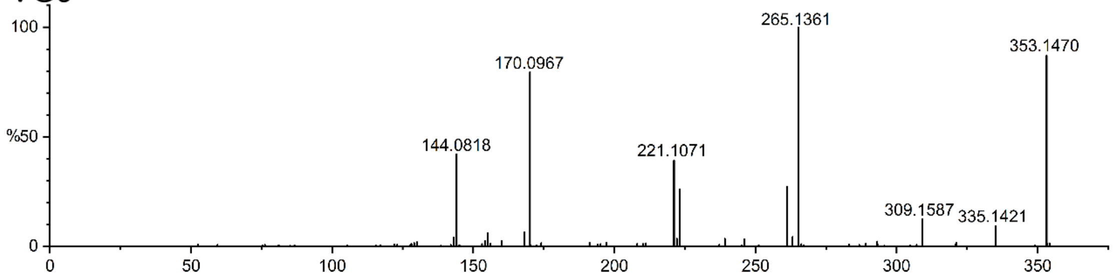

265.1361
353.1470
170.0967
144.0818
221.1071
309.1587
335.1421
%50

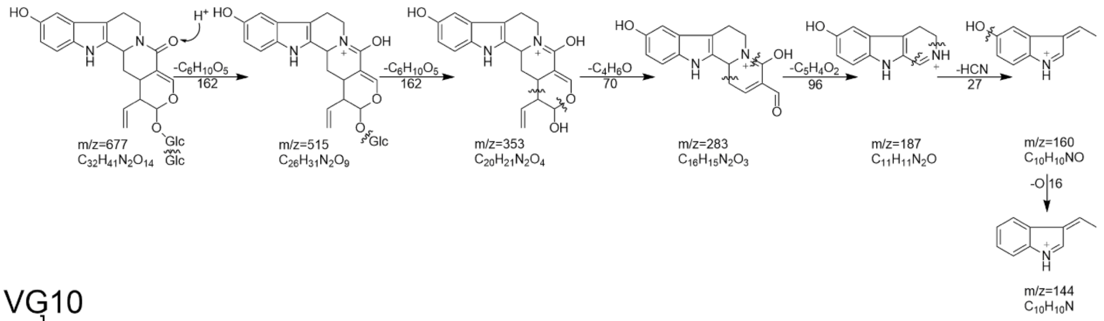
-C6H10O5
162
-C6H10O5
162
-C4H6O
70
-C5H4O2
96
-HCN
27
-O 16
m/z=677
C32H41N2O14
m/z=515
C26H31N2O9
m/z=353
C20H21N2O4
m/z=283
C16H15N2O3
m/z=187
C11H11N2O
m/z=160
C10H10NO
m/z=144
C10H10N

VG10

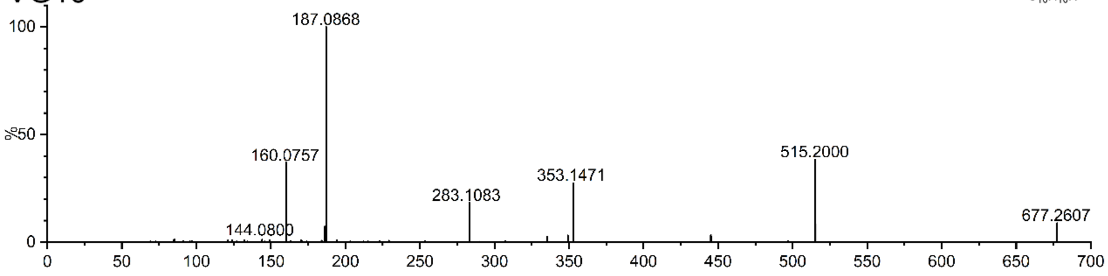
187.0868
160.0757
283.1083
353.1471
515.2000
677.2607
144.0800
%50

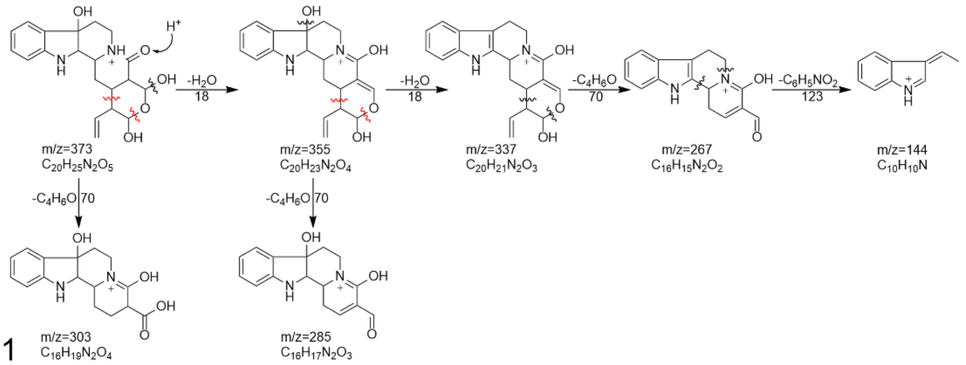
-H2O
18
-H2O
18
-C4H6O
70
-C6H5NO2
123
-C4H6O 70
-C4H6O 70
m/z=373
C20H25N2O5
m/z=355
C20H23N2O4
m/z=337
C20H21N2O3
m/z=267
C16H15N2O2
m/z=144
C10H10N
m/z=303
C16H19N2O4
m/z=285
C16H17N2O3

VG11

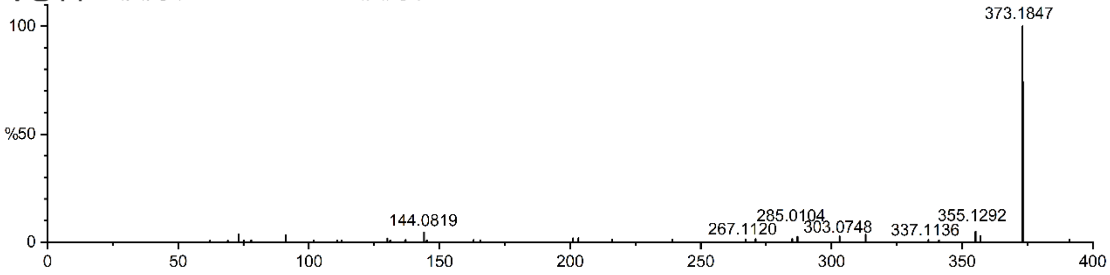
373.1847
144.0819
285.0104
267.1120
303.0748
337.1136
355.1292
%50

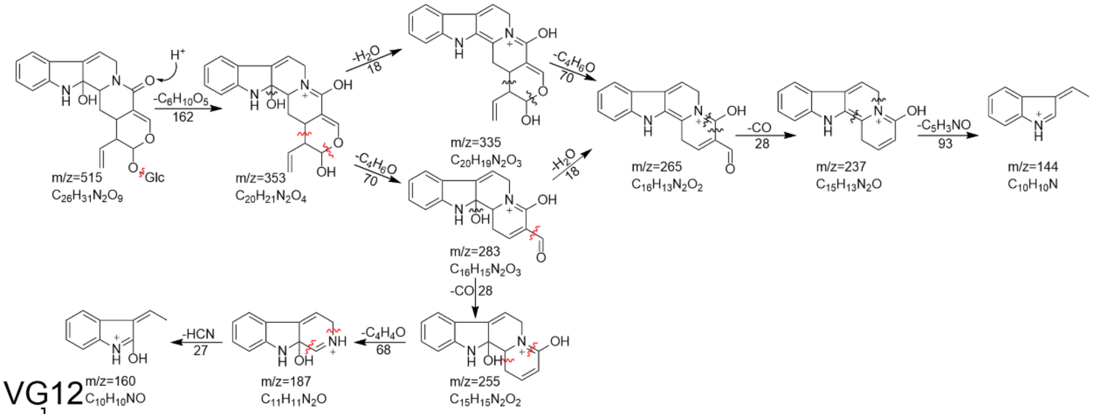
H+
$-C_6H_{10}O_5$
162
$-H_2O$
18
$-C_4H_6O$
70
-CO
28
$-C_5H_3NO$
93
-HCN
27
$-C_4H_4O$
68
-CO 28
m/z=515
$C_{26}H_{31}N_2O_9$
m/z=353
$C_{20}H_{21}N_2O_4$
m/z=335
$C_{20}H_{19}N_2O_3$
m/z=283
$C_{16}H_{15}N_2O_3$
m/z=265
$C_{16}H_{13}N_2O_2$
m/z=237
$C_{15}H_{13}N_2O$
m/z=144
$C_{10}H_{10}N$
m/z=255
$C_{15}H_{15}N_2O_2$
m/z=187
$C_{11}H_{11}N_2O$
m/z=160
$C_{10}H_{10}NO$

VG12

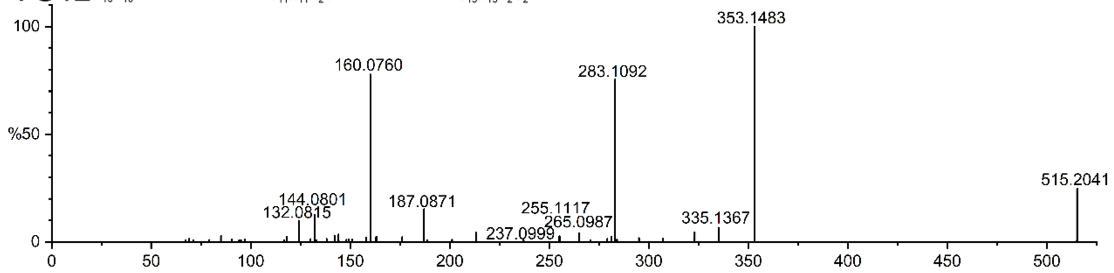
100
%50
0
353.1483
160.0760
283.1092
515.2041
144.0801
132.0815
187.0871
255.1117
265.0987
237.0999
335.1367
0
50
100
150
200
250
300
350
400
450
500

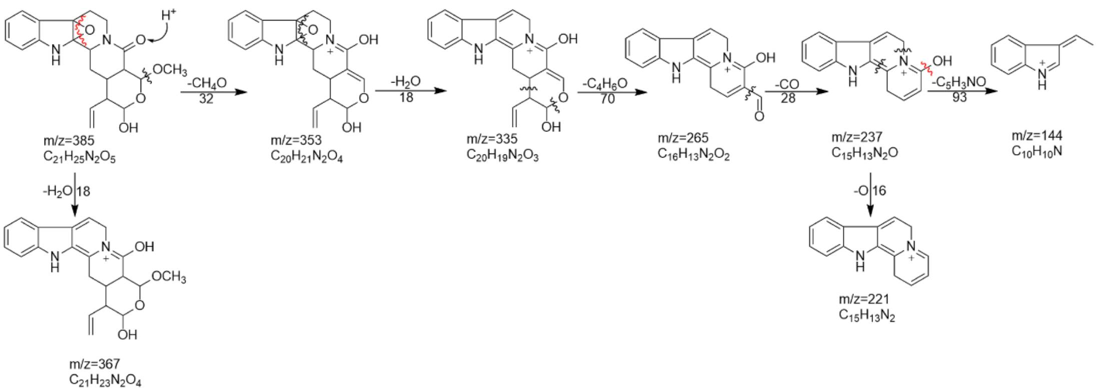
H+
$-CH_4O$
32
$-H_2O$
18
$-C_4H_6O$
70
-CO
28
$-C_5H_3NO$
93
$-H_2O$ 18
-O 16
m/z=385
$C_{21}H_{25}N_2O_5$
m/z=353
$C_{20}H_{21}N_2O_4$
m/z=335
$C_{20}H_{19}N_2O_3$
m/z=265
$C_{16}H_{13}N_2O_2$
m/z=237
$C_{15}H_{13}N_2O$
m/z=144
$C_{10}H_{10}N$
m/z=367
$C_{21}H_{23}N_2O_4$
m/z=221
$C_{15}H_{13}N_2$

VG13

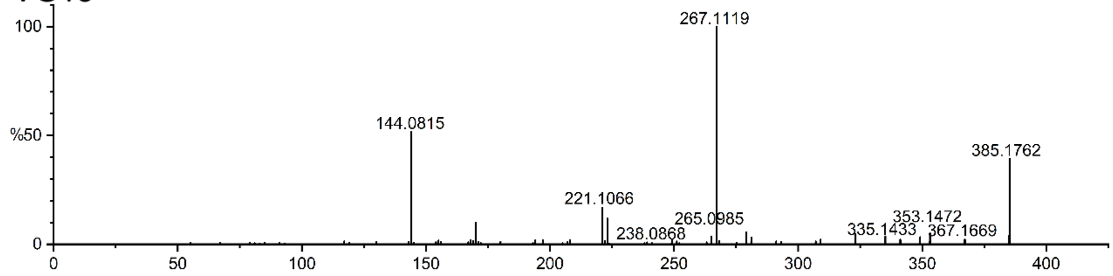
100
%50
0
267.1119
144.0815
385.1762
221.1066
265.0985
238.0868
353.1472
335.1433
367.1669
0
50
100
150
200
250
300
350
400

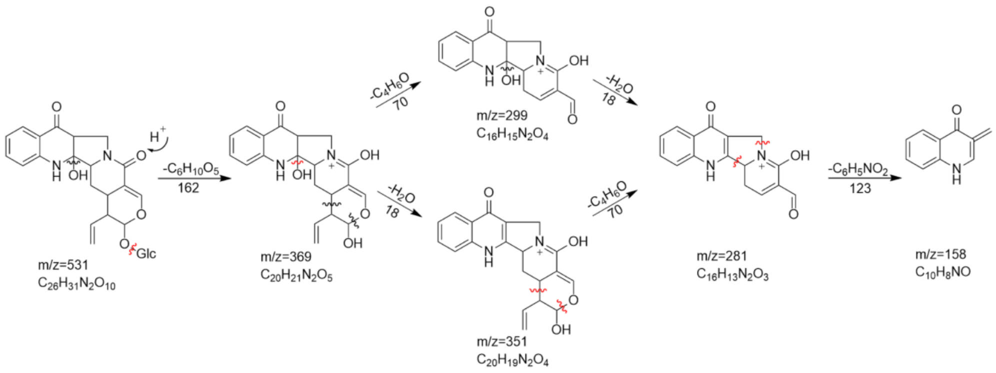
H+
-C6H10O5
162
-C4H6O
70
-H2O
18
-C4H6O
70
-H2O
18
-C6H5NO2
123
m/z=531
C26H31N2O10
m/z=369
C20H21N2O5
m/z=299
C16H15N2O4
m/z=351
C20H19N2O4
m/z=281
C16H13N2O3
m/z=158
C10H8NO
Glc

PG2

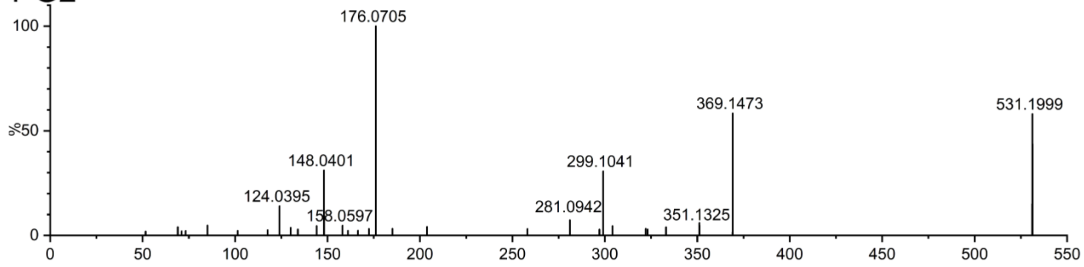
176.0705
369.1473
531.1999
148.0401
299.1041
124.0395
281.0942
158.0597
351.1325
%

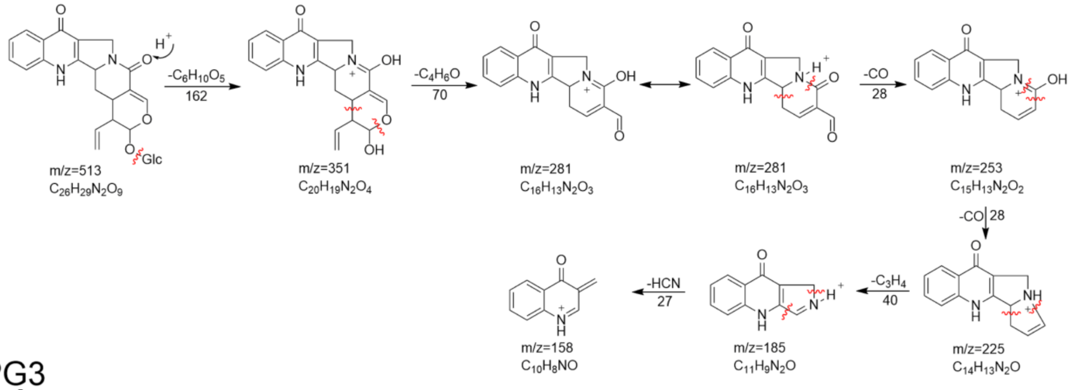
H+
-C6H10O5
162
-C4H6O
70
-CO
28
-CO 28
-C3H4
40
-HCN
27
m/z=513
C26H29N2O9
m/z=351
C20H19N2O4
m/z=281
C16H13N2O3
m/z=281
C16H13N2O3
m/z=253
C15H13N2O2
m/z=225
C14H13N2O
m/z=185
C11H9N2O
m/z=158
C10H8NO
Glc

PG3

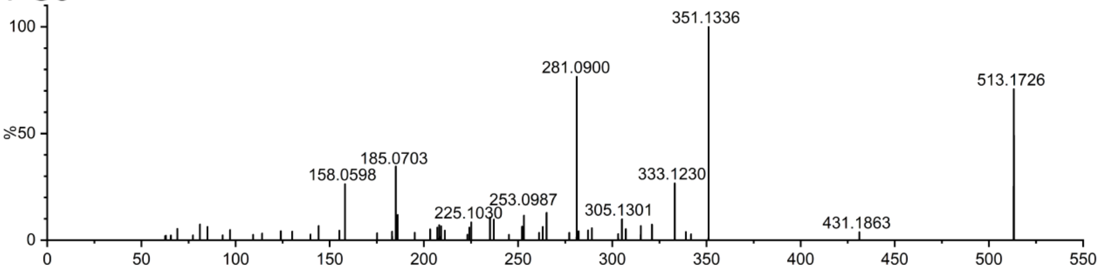
351.1336
281.0900
513.1726
185.0703
158.0598
333.1230
253.0987
225.1030
305.1301
431.1863
%

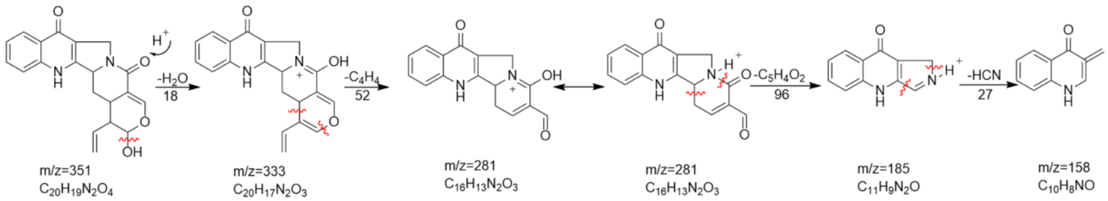
-H2O
18
-C4H4
52
-C5H4O2
96
-HCN
27
m/z=351
C20H19N2O4
m/z=333
C20H17N2O3
m/z=281
C16H13N2O3
m/z=281
C16H13N2O3
m/z=185
C11H9N2O
m/z=158
C10H8NO

PG4

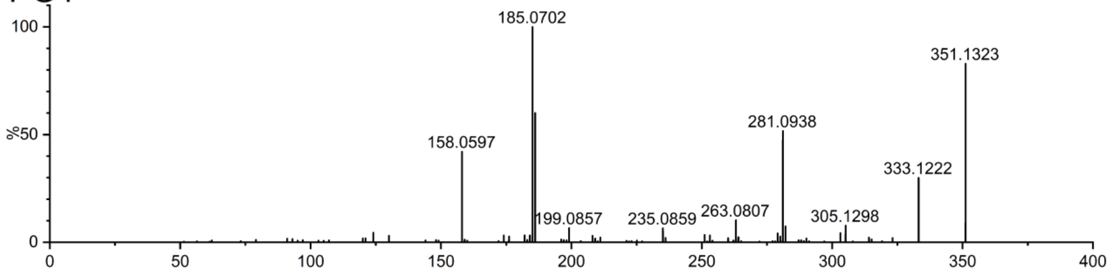
185.0702
351.1323
281.0938
158.0597
333.1222
199.0857
235.0859
263.0807
305.1298
%

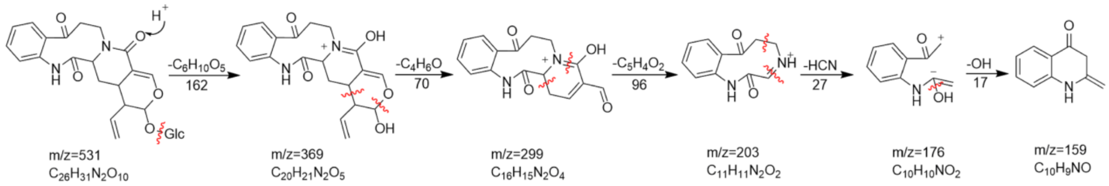
-C6H10O5
162
-C4H6O
70
-C5H4O2
96
-HCN
27
-OH
17
Glc
m/z=531
C26H31N2O10
m/z=369
C20H21N2O5
m/z=299
C16H15N2O4
m/z=203
C11H11N2O2
m/z=176
C10H10NO2
m/z=159
C10H9NO

PG5

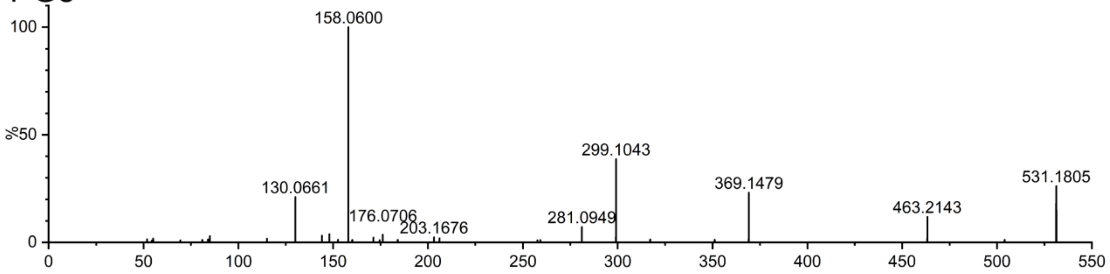
158.0600
299.1043
369.1479
531.1805
130.0661
463.2143
176.0706
203.1676
281.0949
%

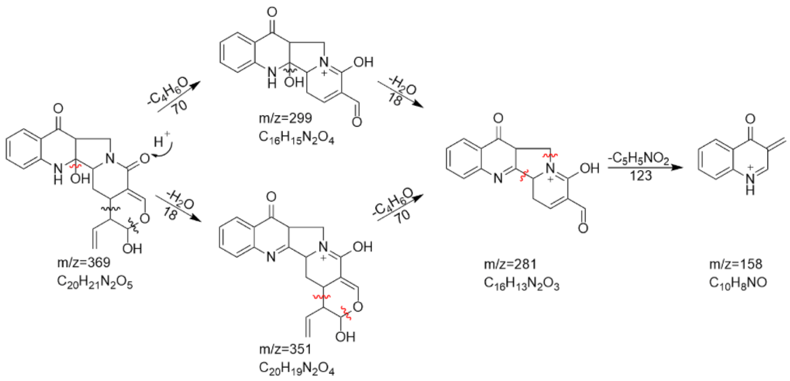
-C4H6O
70
-H2O
18
-H2O
18
-C4H6O
70
-C5H5NO2
123
m/z=369
C20H21N2O5
m/z=299
C16H15N2O4
m/z=351
C20H19N2O4
m/z=281
C16H13N2O3
m/z=158
C10H8NO

PG6

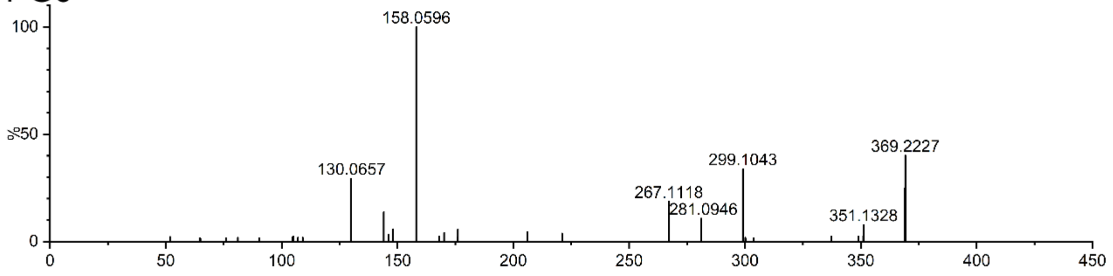
158.0596
369.2227
299.1043
130.0657
267.1118
281.0946
351.1328
%

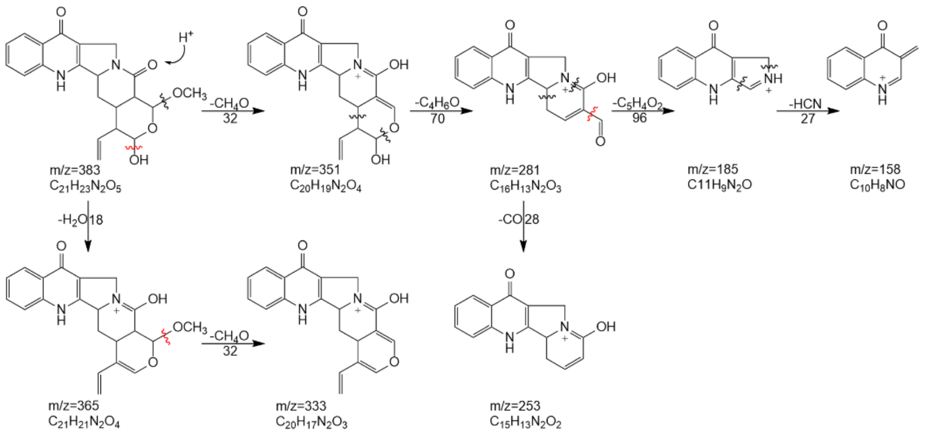
m/z=383
C21H23N2O5
-CH4O
32
m/z=351
C20H19N2O4
-C4H6O
70
m/z=281
C16H13N2O3
-C5H4O2
96
m/z=185
C11H9N2O
-HCN
27
m/z=158
C10H8NO
-H2O 18
-CO 28
-CH4O
32
m/z=365
C21H21N2O4
m/z=333
C20H17N2O3
m/z=253
C15H13N2O2

PG7

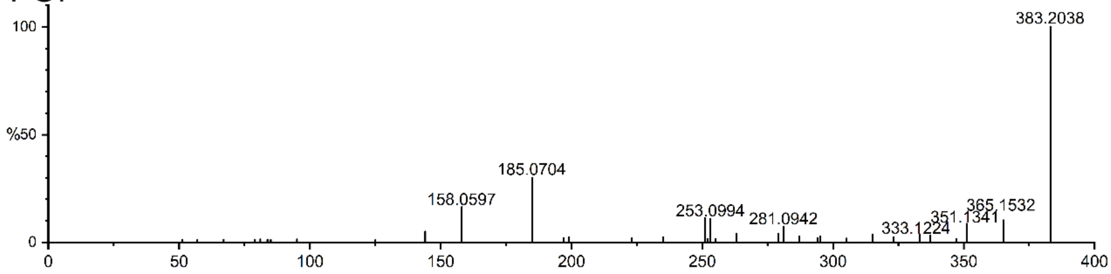
383.2038
185.0704
158.0597
253.0994
281.0942
333.1224
351.1341
365.1532
%50
100
0
0
50
100
150
200
250
300
350
400

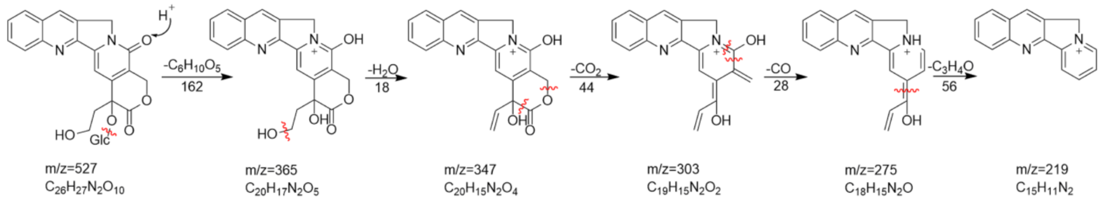
m/z=527
C26H27N2O10
-C6H10O5
162
m/z=365
C20H17N2O5
-H2O
18
m/z=347
C20H15N2O4
-CO2
44
m/z=303
C19H15N2O2
-CO
28
m/z=275
C18H15N2O
-C3H4O
56
m/z=219
C15H11N2

CG1

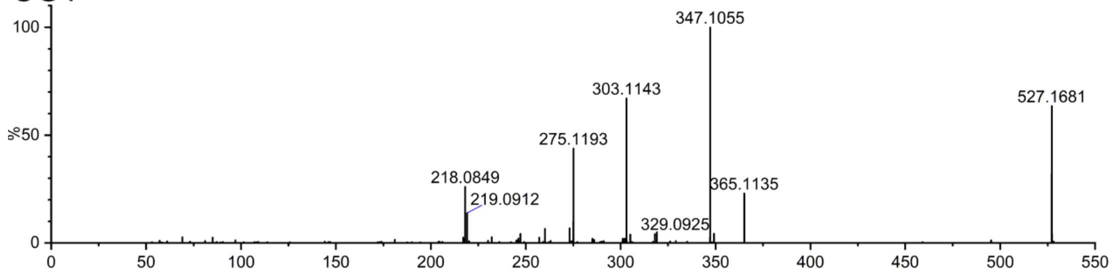
347.1055
303.1143
527.1681
275.1193
218.0849
219.0912
365.1135
329.0925
%50
100
0
0
50
100
150
200
250
300
350
400
450
500
550

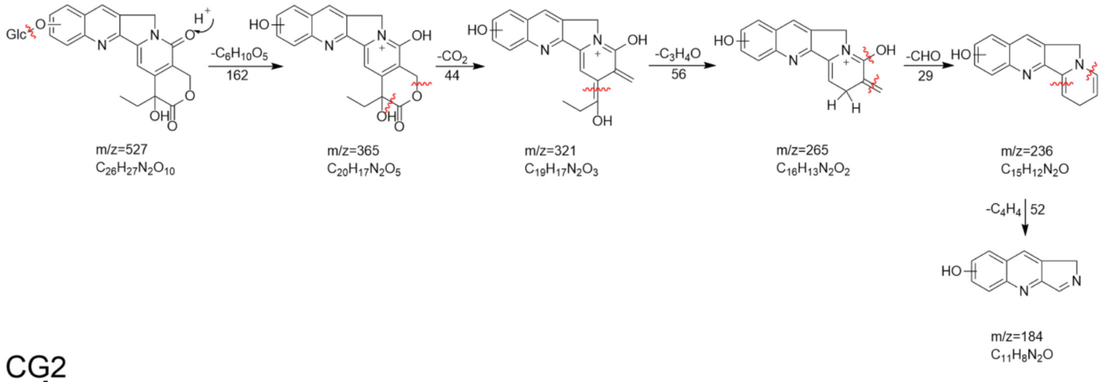
m/z=527
$C_{26}H_{27}N_2O_{10}$
$-C_6H_{10}O_5$ 162
m/z=365
$C_{20}H_{17}N_2O_5$
$-CO_2$ 44
m/z=321
$C_{19}H_{17}N_2O_3$
$-C_3H_4O$ 56
m/z=265
$C_{16}H_{13}N_2O_2$
-CHO 29
m/z=236
$C_{15}H_{12}N_2O$
$-C_4H_4$ 52
m/z=184
$C_{11}H_8N_2O$

CG2

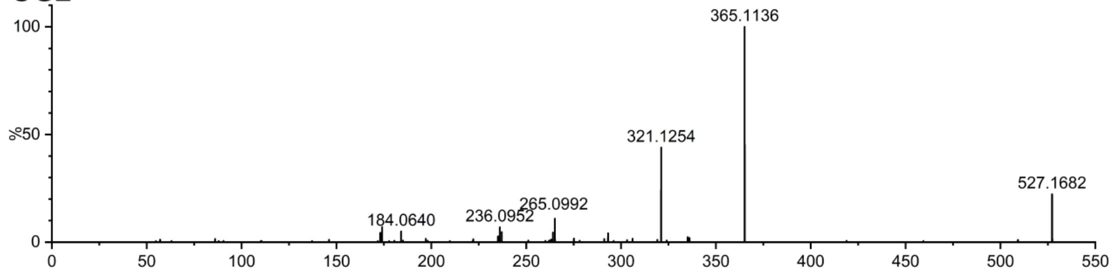
365.1136
321.1254
527.1682
265.0992
236.0952
184.0640
%50

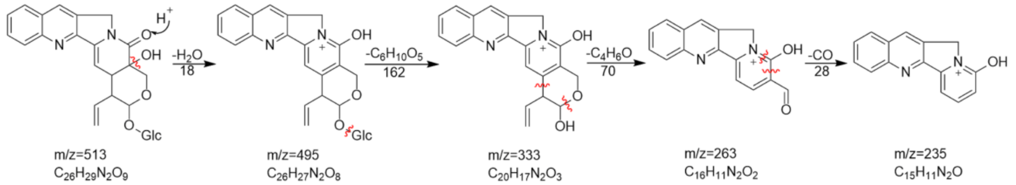
m/z=513
$C_{26}H_{29}N_2O_9$
$-H_2O$ 18
m/z=495
$C_{26}H_{27}N_2O_8$
$-C_6H_{10}O_5$ 162
m/z=333
$C_{20}H_{17}N_2O_3$
$-C_4H_6O$ 70
m/z=263
$C_{16}H_{11}N_2O_2$
-CO 28
m/z=235
$C_{15}H_{11}N_2O$

CG3

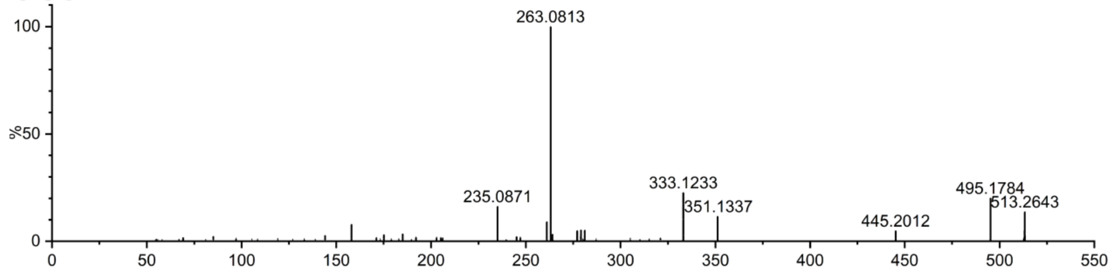
263.0813
235.0871
333.1233
351.1337
445.2012
495.1784
513.2643
%50

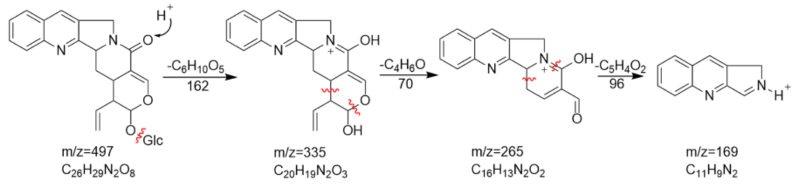
m/z=497
$C_{26}H_{29}N_2O_8$
$-C_6H_{10}O_5$ 162
m/z=335
$C_{20}H_{19}N_2O_3$
$-C_4H_6O$ 70
m/z=265
$C_{16}H_{13}N_2O_2$
$-C_5H_4O_2$ 96
m/z=169
$C_{11}H_9N_2$

CG4

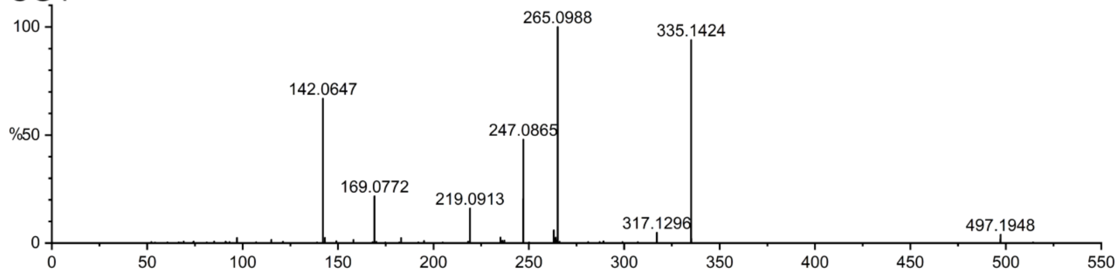
265.0988
335.1424
142.0647
247.0865
169.0772
219.0913
317.1296
497.1948
%50

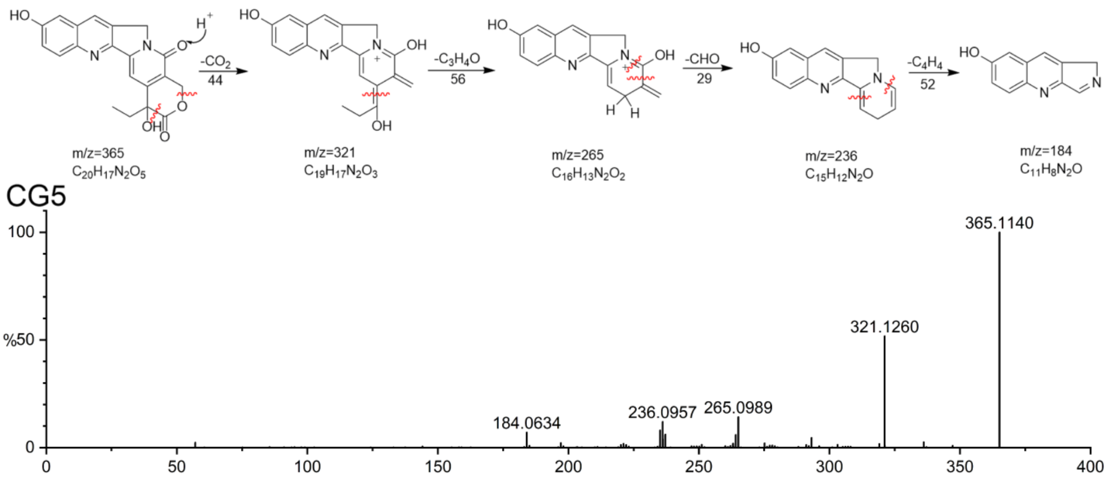

-CO2
44
-C3H4O
56
-CHO
29
-C4H4
52
m/z=365
C20H17N2O5
m/z=321
C19H17N2O3
m/z=265
C16H13N2O2
m/z=236
C15H12N2O
m/z=184
C11H8N2O
CG5
365.1140
321.1260
184.0634
236.0957
265.0989

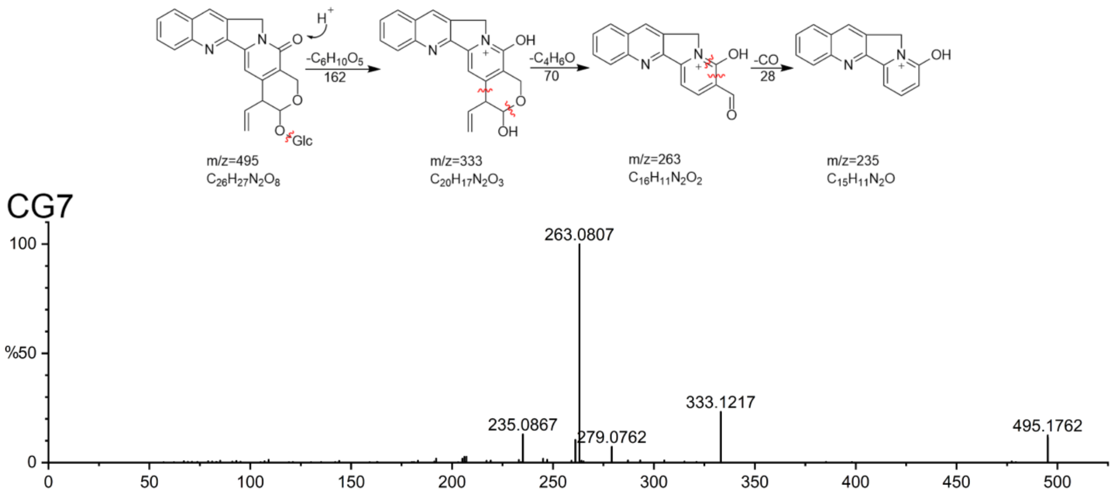

-C6H10O5
162
-C4H6O
70
-CO
28
Glc
m/z=495
C26H27N2O8
m/z=333
C20H17N2O3
m/z=263
C16H11N2O2
m/z=235
C15H11N2O
CG7
263.0807
333.1217
235.0867
279.0762
495.1762

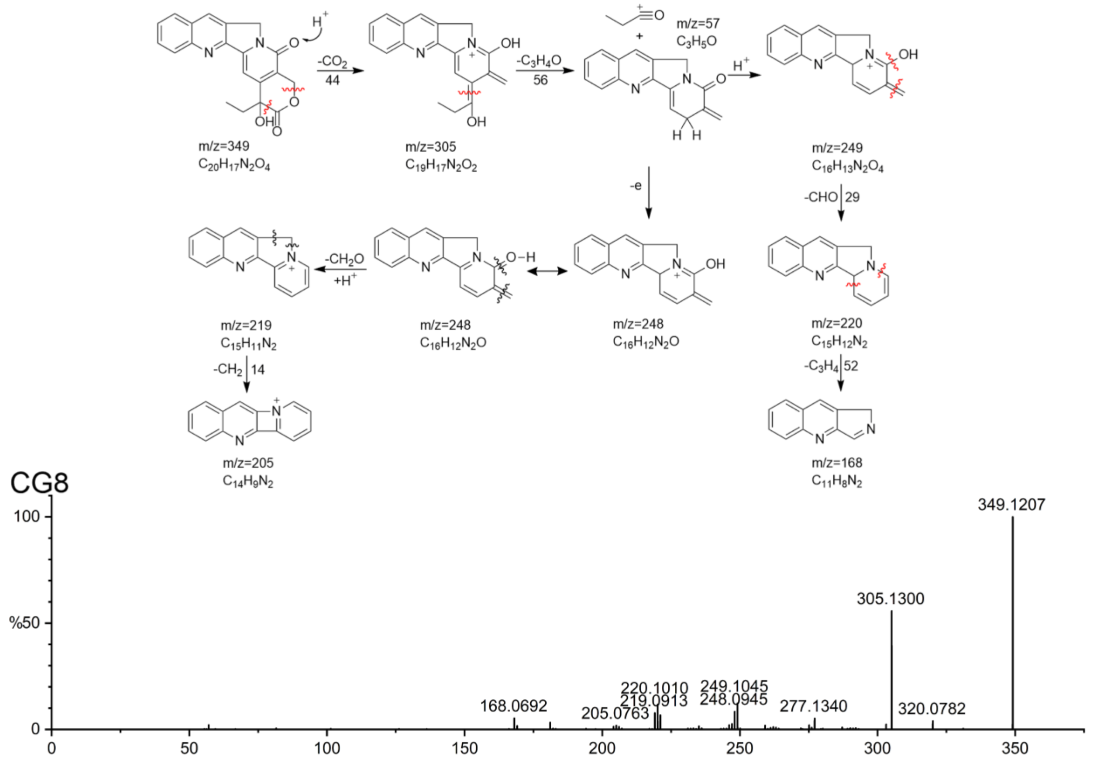
H+
-CO2
44
-C3H4O
56
m/z=57
C3H5O
H+
OH
m/z=349
C20H17N2O4
m/z=305
C19H17N2O2
m/z=249
C16H13N2O4
-e
-CHO 29
-CH2O
+H+
O–H
m/z=219
C15H11N2
m/z=248
C16H12N2O
m/z=248
C16H12N2O
m/z=220
C15H12N2
-CH2 14
-C3H4 52
m/z=205
C14H9N2
m/z=168
C11H8N2
CG8
100
%50
0
349.1207
305.1300
220.1010
219.0913
249.1045
248.0945
168.0692
205.0763
277.1340
320.0782
0 50 100 150 200 250 300 350

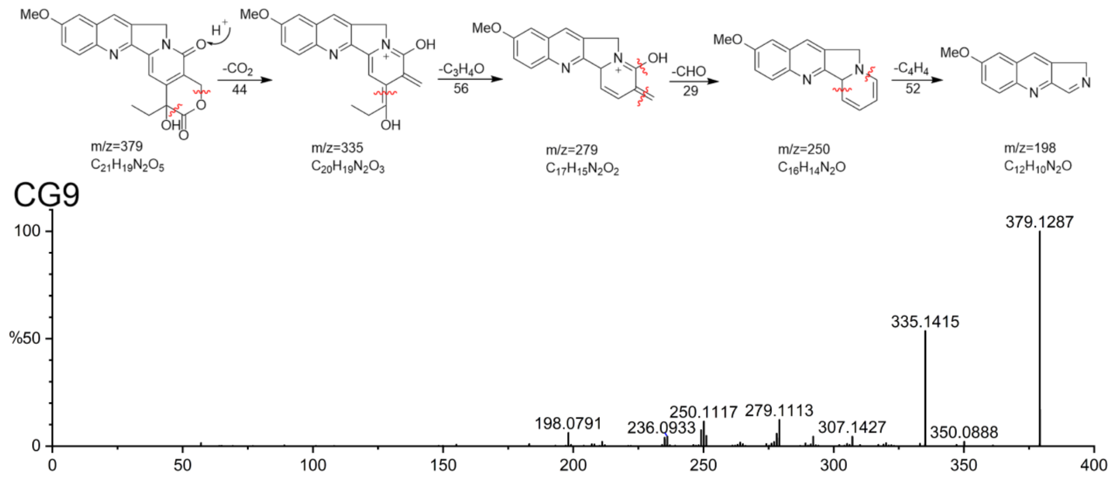
MeO
H+
-CO2
44
-C3H4O
56
-CHO
29
-C4H4
52
OH
m/z=379
C21H19N2O5
m/z=335
C20H19N2O3
m/z=279
C17H15N2O2
m/z=250
C16H14N2O
m/z=198
C12H10N2O
CG9
100
%50
0
379.1287
335.1415
198.0791
236.0933
250.1117
279.1113
307.1427
350.0888
0 50 100 150 200 250 300 350 400

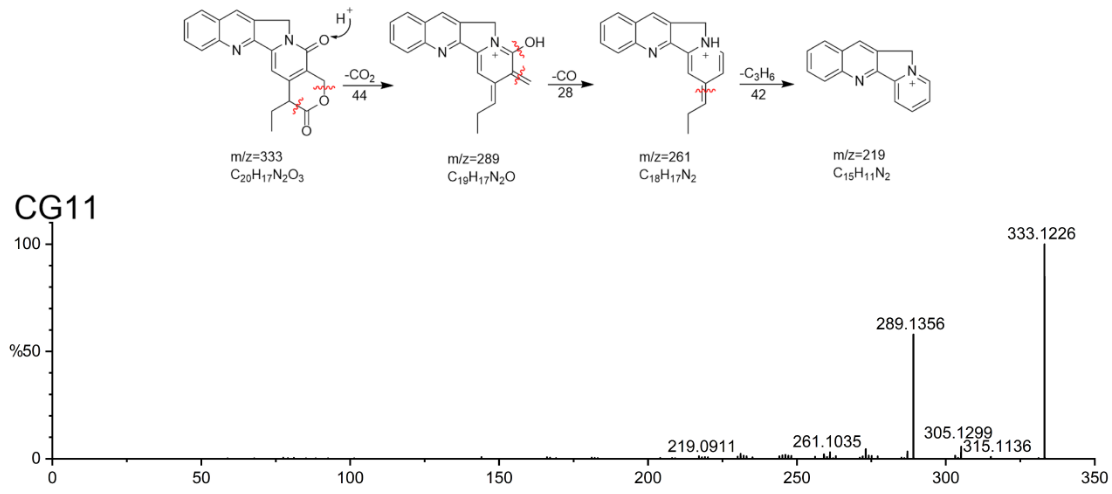
-CO2
44
-CO
28
-C3H6
42
m/z=333
C20H17N2O3
m/z=289
C19H17N2O
m/z=261
C18H17N2
m/z=219
C15H11N2
CG11
100
%50
0
333.1226
289.1356
305.1299
315.1136
261.1035
219.0911
0
50
100
150
200
250
300
350

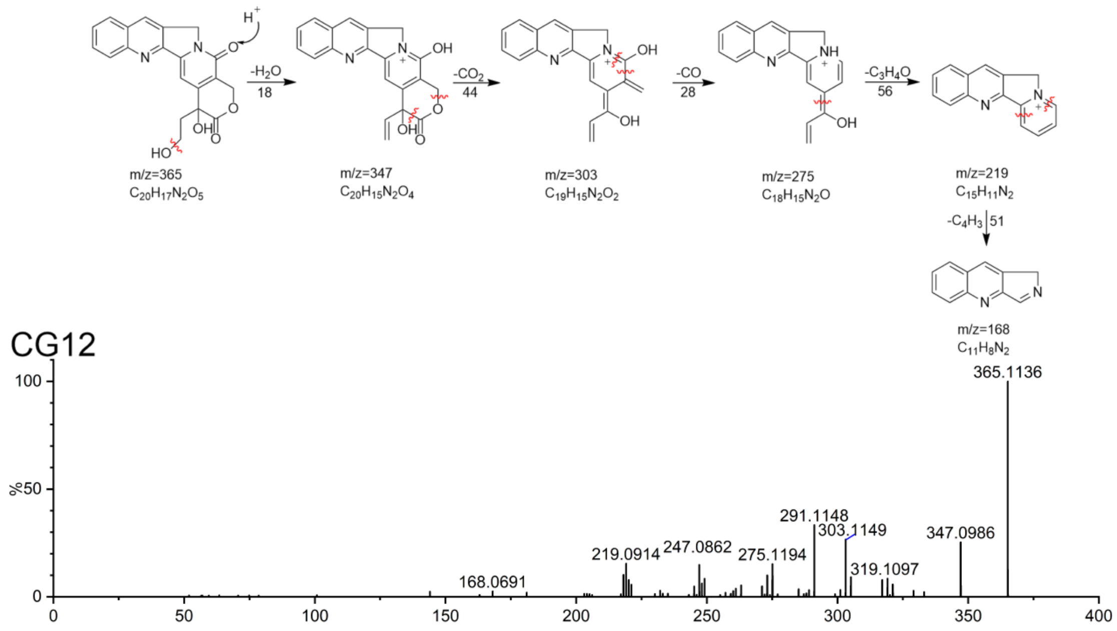
-H2O
18
-CO2
44
-CO
28
-C3H4O
56
-C4H3
51
m/z=365
C20H17N2O5
m/z=347
C20H15N2O4
m/z=303
C19H15N2O2
m/z=275
C18H15N2O
m/z=219
C15H11N2
m/z=168
C11H8N2
CG12
100
%50
0
365.1136
291.1148
303.1149
347.0986
219.0914
247.0862
275.1194
319.1097
168.0691
0
50
100
150
200
250
300
350
400

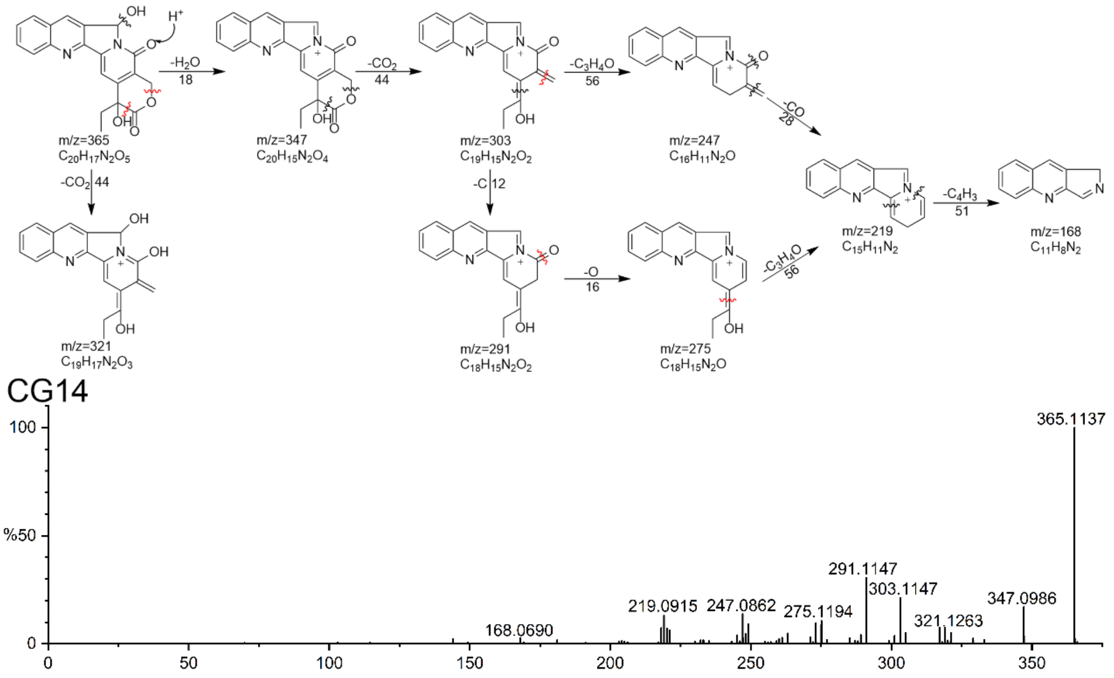

m/z=365
C20H17N2O5
-H2O
18
m/z=347
C20H15N2O4
-CO2
44
m/z=303
C19H15N2O2
-C3H4O
56
m/z=247
C16H11N2O
-CO
28
m/z=219
C15H11N2
-C4H3
51
m/z=168
C11H8N2
-CO2 44
m/z=321
C19H17N2O3
-C 12
m/z=291
C18H15N2O2
-O
16
m/z=275
C18H15N2O
-C3H4O
56
CG14
100
%50
0
365.1137
291.1147
303.1147
347.0986
219.0915
247.0862
275.1194
321.1263
168.0690
0
50
100
150
200
250
300
350

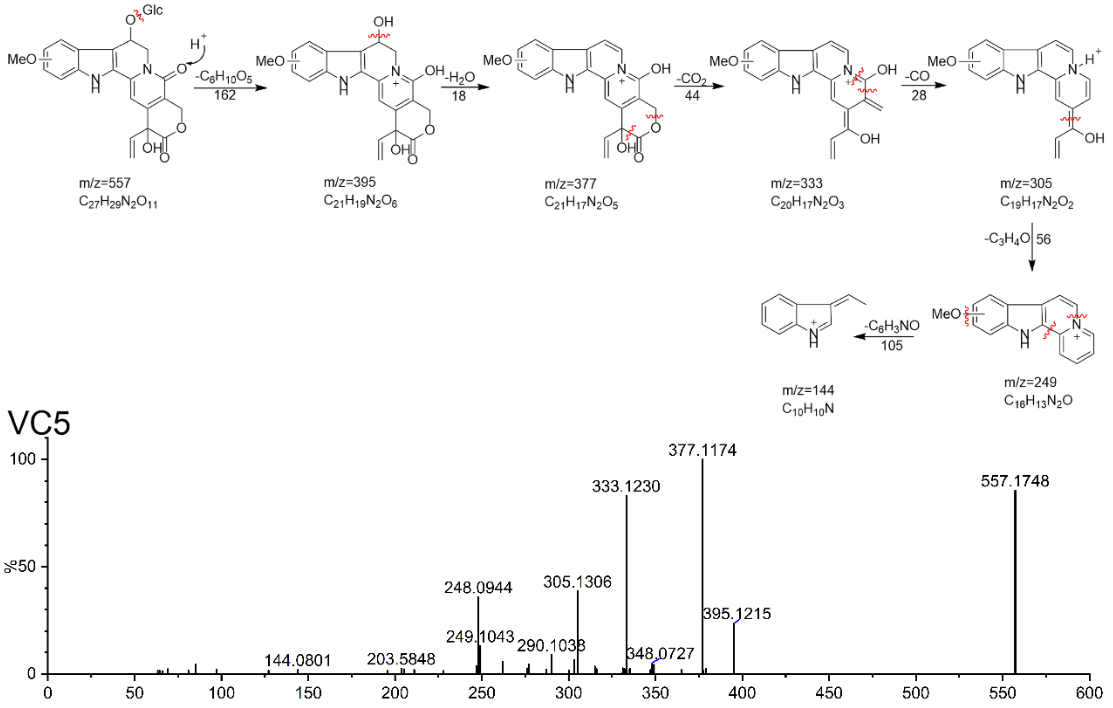

MeO
Glc
m/z=557
C27H29N2O11
-C6H10O5
162
m/z=395
C21H19N2O6
-H2O
18
m/z=377
C21H17N2O5
-CO2
44
m/z=333
C20H17N2O3
-CO
28
m/z=305
C19H17N2O2
-C3H4O 56
m/z=249
C16H13N2O
-C6H3NO
105
m/z=144
C10H10N
VC5
100
%50
0
377.1174
333.1230
557.1748
248.0944
305.1306
395.1215
249.1043
290.1038
348.0727
144.0801
203.5848
0
50
100
150
200
250
300
350
400
450
500
550
600

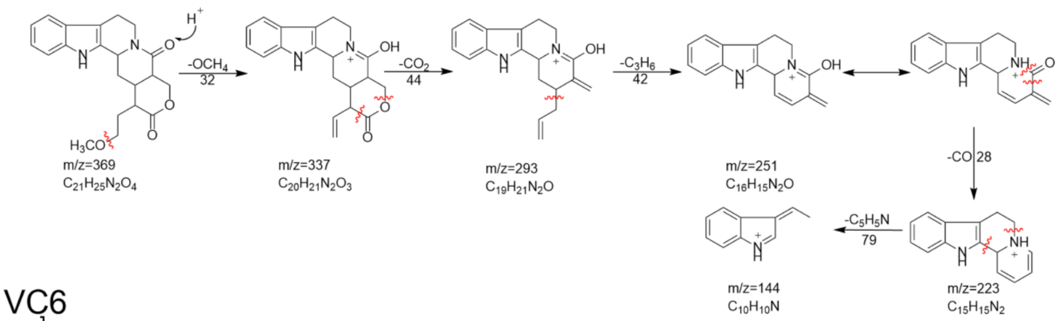
H+
-OCH4
32
-CO2
44
-C3H6
42
-CO 28
-C5H5N
79
m/z=369
C21H25N2O4
m/z=337
C20H21N2O3
m/z=293
C19H21N2O
m/z=251
C16H15N2O
m/z=223
C15H15N2
m/z=144
C10H10N

VC6

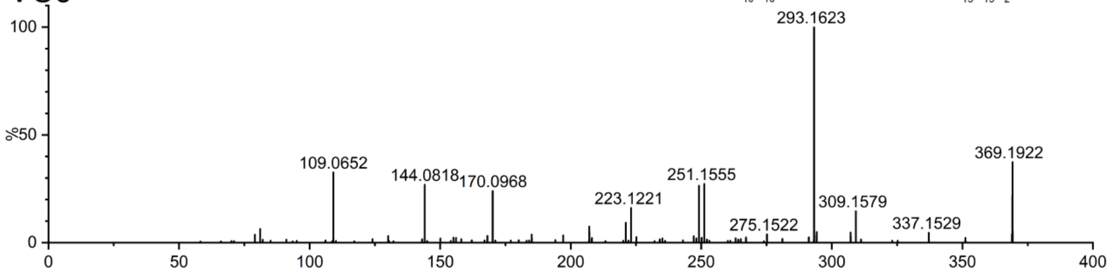
100
%50
0
293.1623
369.1922
109.0652
144.0818
170.0968
251.1555
223.1221
309.1579
337.1529
275.1522
0
50
100
150
200
250
300
350
400

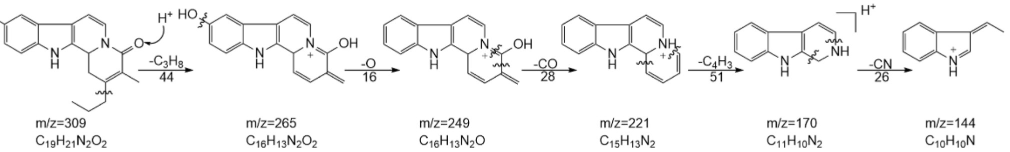
H+
-C3H8
44
-O
16
-CO
28
-C4H3
51
-CN
26
m/z=309
C19H21N2O2
m/z=265
C16H13N2O2
m/z=249
C16H13N2O
m/z=221
C15H13N2
m/z=170
C11H10N2
m/z=144
C10H10N

VC7

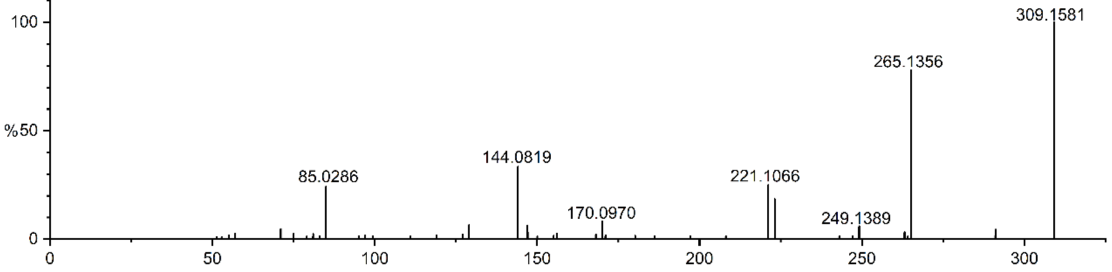
100
%50
0
309.1581
265.1356
144.0819
85.0286
221.1066
170.0970
249.1389
0
50
100
150
200
250
300

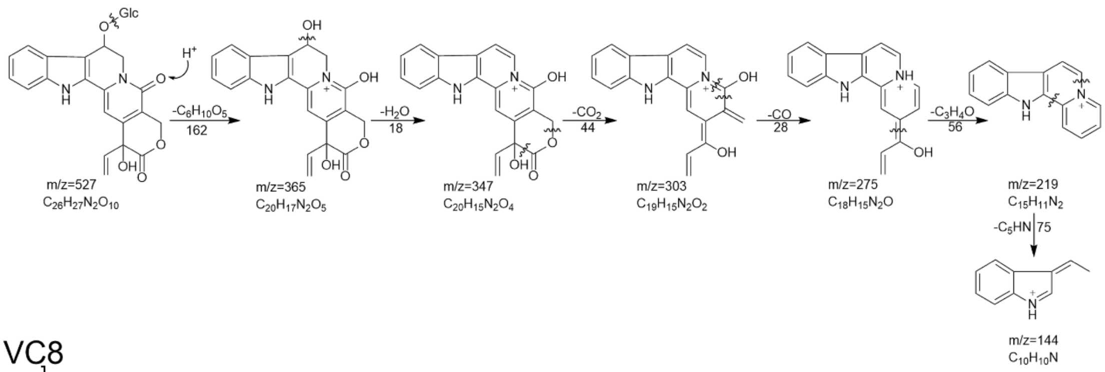
Glc
H+
-C6H10O5
162
-H2O
18
-CO2
44
-CO
28
-C3H4O
56
-C5HN 75
m/z=527
C26H27N2O10
m/z=365
C20H17N2O5
m/z=347
C20H15N2O4
m/z=303
C19H15N2O2
m/z=275
C18H15N2O
m/z=219
C15H11N2
m/z=144
C10H10N

VC8

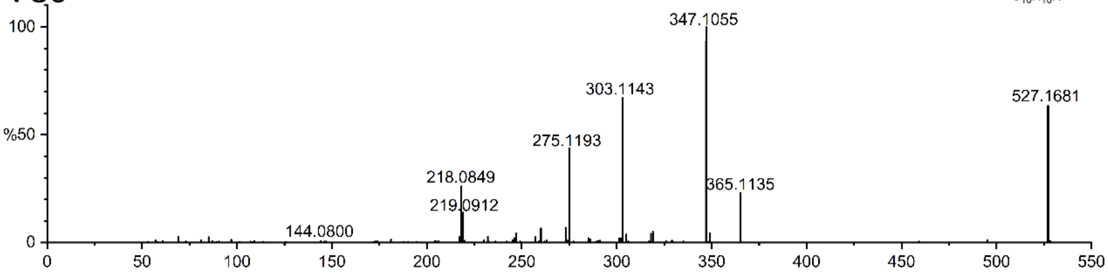
100
%50
0
347.1055
303.1143
527.1681
275.1193
218.0849
219.0912
365.1135
144.0800
0
50
100
150
200
250
300
350
400
450
500
550

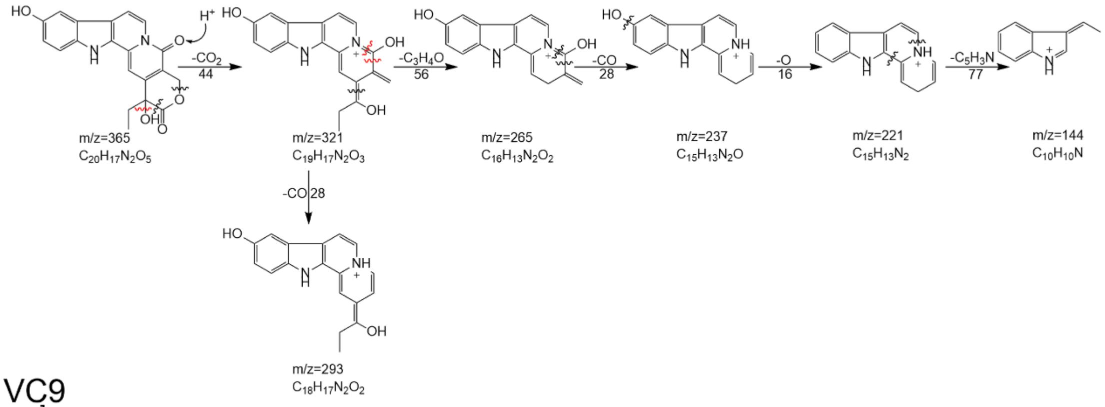
H+
-CO2 44
-C3H4O 56
-CO 28
-O 16
-C5H3N 77
-CO 28
m/z=365 C20H17N2O5
m/z=321 C19H17N2O3
m/z=265 C16H13N2O2
m/z=237 C15H13N2O
m/z=221 C15H13N2
m/z=144 C10H10N
m/z=293 C18H17N2O2

VC9

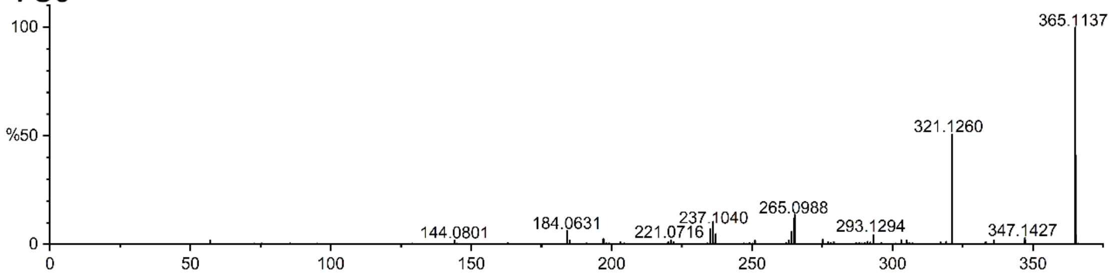
100
%50
0
365.1137
321.1260
265.0988
237.1040
293.1294
347.1427
184.0631
221.0716
144.0801
0
50
100
150
200
250
300
350

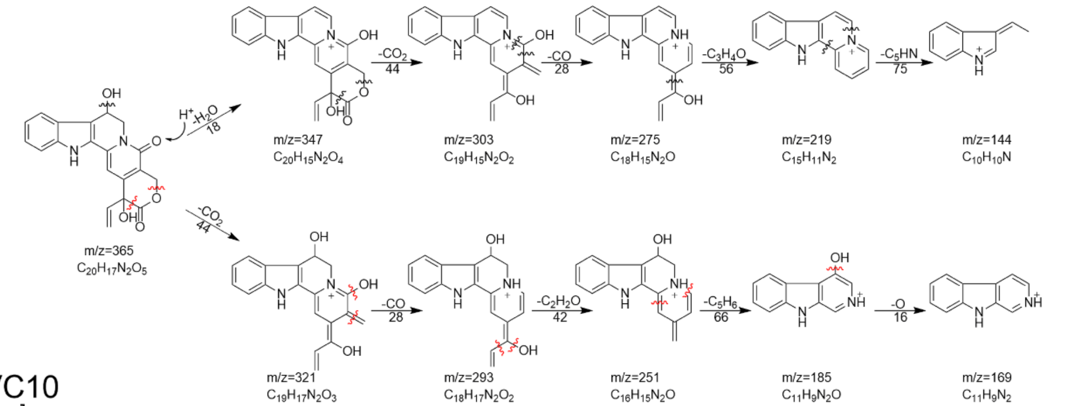
H+ -H2O 18
-CO2 44
-CO 28
-C3H4O 56
-C5HN 75
-C2H2O 42
-C5H6 66
-O 16
m/z=347 C20H15N2O4
m/z=303 C19H15N2O2
m/z=275 C18H15N2O
m/z=219 C15H11N2
m/z=144 C10H10N
m/z=365 C20H17N2O5
m/z=321 C19H17N2O3
m/z=293 C18H17N2O2
m/z=251 C16H15N2O
m/z=185 C11H9N2O
m/z=169 C11H9N2

VC10

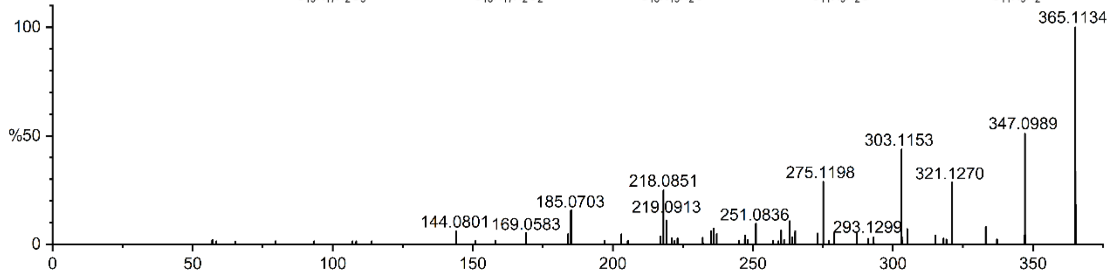
100
%50
0
365.1134
347.0989
303.1153
321.1270
275.1198
218.0851
219.0913
251.0836
293.1299
185.0703
144.0801
169.0583
0
50
100
150
200
250
300
350

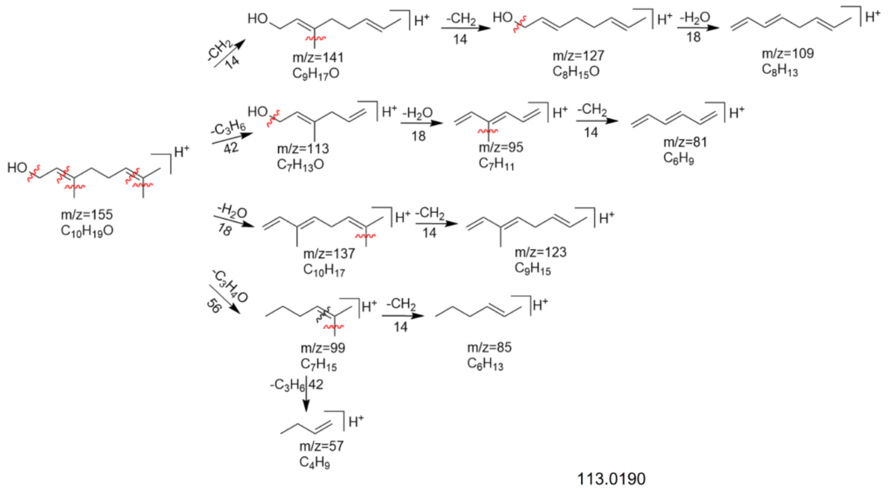
m/z=155
$C_{10}H_{19}O$
$-CH_2$ 14
m/z=141
$C_9H_{17}O$
$-CH_2$ 14
m/z=127
$C_8H_{15}O$
$-H_2O$ 18
m/z=109
C_8H_{13}
$-C_3H_6$ 42
m/z=113
$C_7H_{13}O$
$-H_2O$ 18
m/z=95
C_7H_{11}
$-CH_2$ 14
m/z=81
C_6H_9
$-H_2O$ 18
m/z=137
$C_{10}H_{17}$
$-CH_2$ 14
m/z=123
C_9H_{15}
$-C_3H_4O$ 56
m/z=99
C_7H_{15}
$-CH_2$ 14
m/z=85
C_6H_{13}
$-C_3H_6$ 42
m/z=57
C_4H_9

IP1

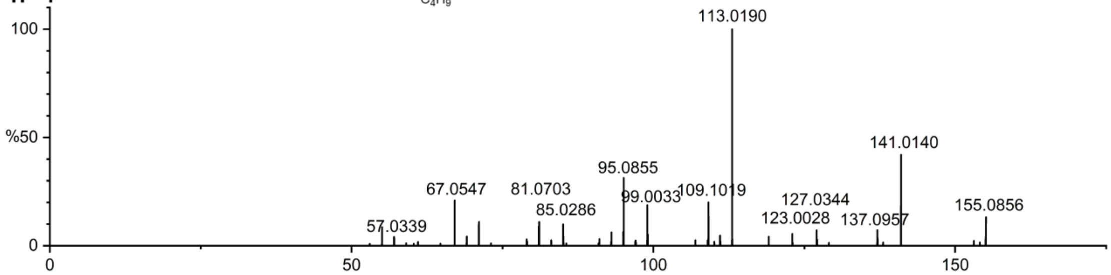
100
%50
0
113.0190
141.0140
95.0855
67.0547
81.0703
99.0033
109.1019
127.0344
155.0856
85.0286
123.0028
137.0957
57.0339
0
50
100
150

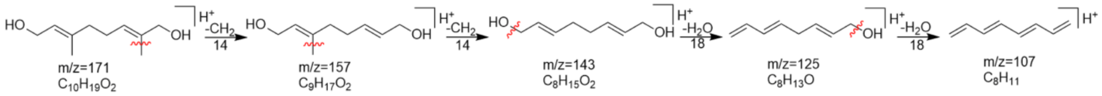
m/z=171
$C_{10}H_{19}O_2$
$-CH_2$ 14
m/z=157
$C_9H_{17}O_2$
$-CH_2$ 14
m/z=143
$C_8H_{15}O_2$
$-H_2O$ 18
m/z=125
$C_8H_{13}O$
$-H_2O$ 18
m/z=107
C_8H_{11}

IP2

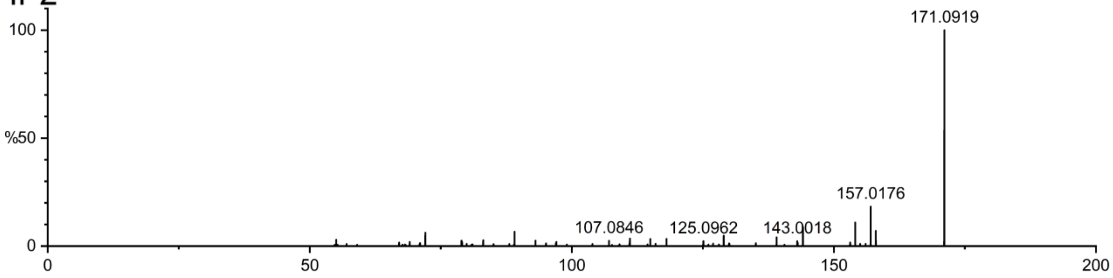
100
%50
0
171.0919
157.0176
107.0846
125.0962
143.0018
0
50
100
150
200

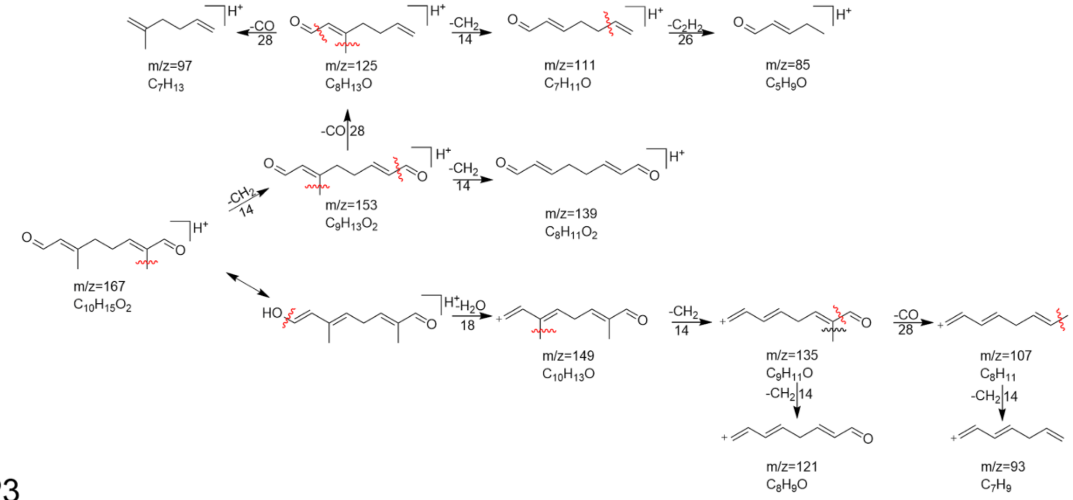
m/z=97
C7H13
-CO 28
m/z=125
C8H13O
-CH2 14
m/z=111
C7H11O
-C2H2 26
m/z=85
C5H9O
-CO 28
m/z=153
C9H13O2
-CH2 14
m/z=139
C8H11O2
m/z=167
C10H15O2
H2O 18
m/z=149
C10H13O
-CH2 14
m/z=135
C9H11O
-CO 28
m/z=107
C8H11
-CH2 14
m/z=121
C8H9O
m/z=93
C7H9

IP3

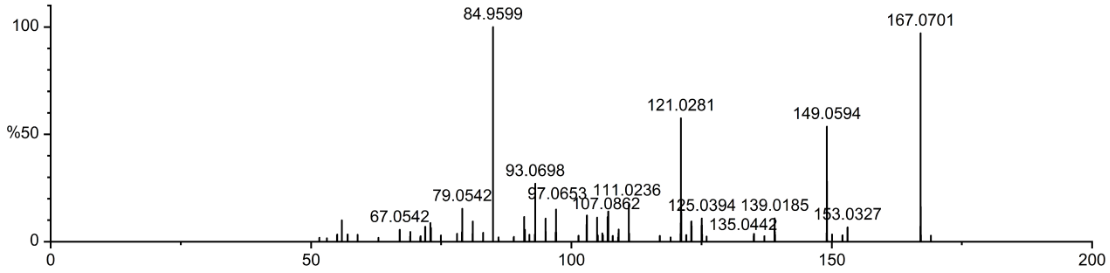
100
%50
0
84.9599
167.0701
121.0281
149.0594
93.0698
79.0542
97.0653
111.0236
107.0862
67.0542
125.0394
139.0185
135.0442
153.0327
0
50
100
150
200

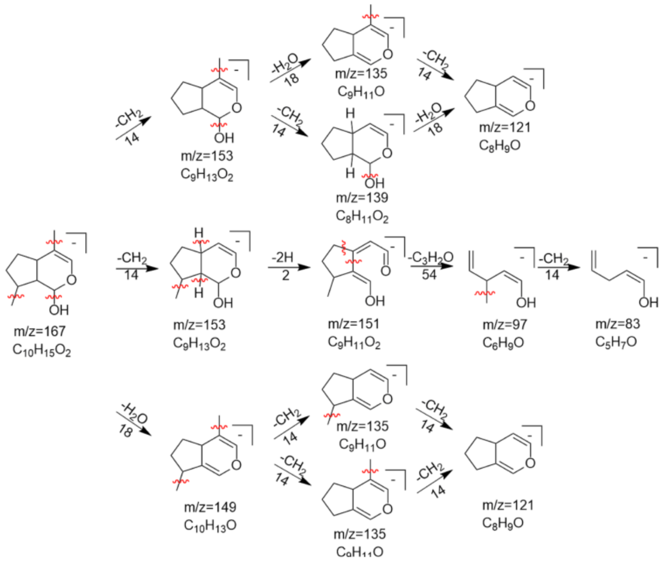
-H2O 18
m/z=135
C9H11O
-CH2 14
-CH2 14
m/z=153
C9H13O2
-CH2 14
-H2O 18
m/z=121
C8H9O
m/z=139
C8H11O2
m/z=167
C10H15O2
-CH2 14
m/z=153
C9H13O2
-2H 2
m/z=151
C9H11O2
-C3H2O 54
m/z=97
C6H9O
-CH2 14
m/z=83
C5H7O
-H2O 18
m/z=149
C10H13O
-CH2 14
m/z=135
C9H11O
-CH2 14
-CH2 14
m/z=135
-CH2 14
m/z=121
C8H9O

IP4

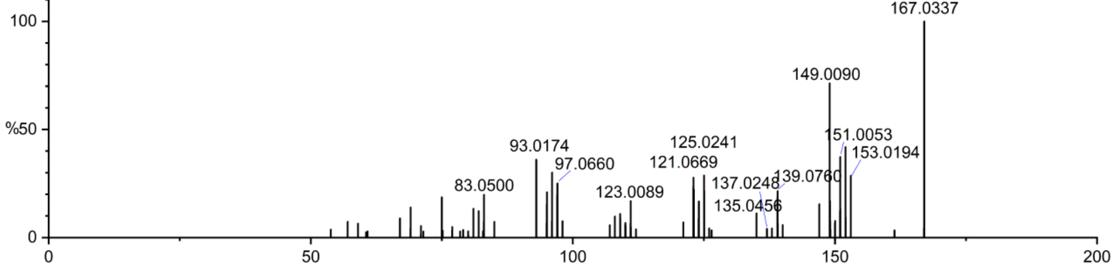
100
%50
0
167.0337
149.0090
151.0053
153.0194
93.0174
125.0241
97.0660
121.0669
83.0500
139.0760
137.0248
123.0089
135.0456
0
50
100
150
200

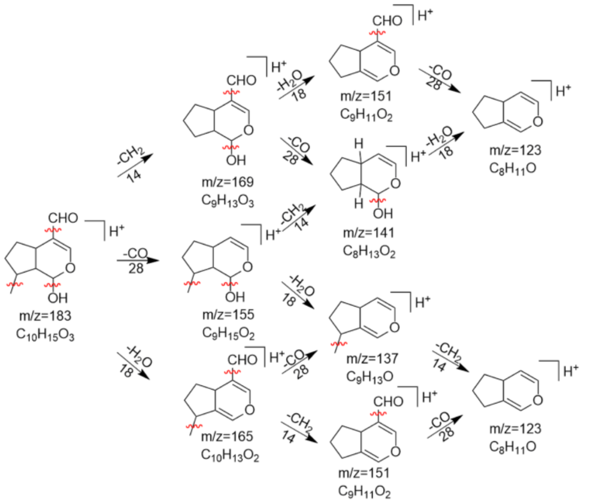
m/z=183
$C_{10}H_{15}O_3$
m/z=169
$C_9H_{13}O_3$
m/z=151
$C_9H_{11}O_2$
m/z=123
$C_8H_{11}O$
m/z=141
$C_8H_{13}O_2$
m/z=155
$C_9H_{15}O_2$
m/z=137
$C_9H_{13}O$
m/z=165
$C_{10}H_{13}O_2$
m/z=151
$C_9H_{11}O_2$
m/z=123
$C_8H_{11}O$

IP5

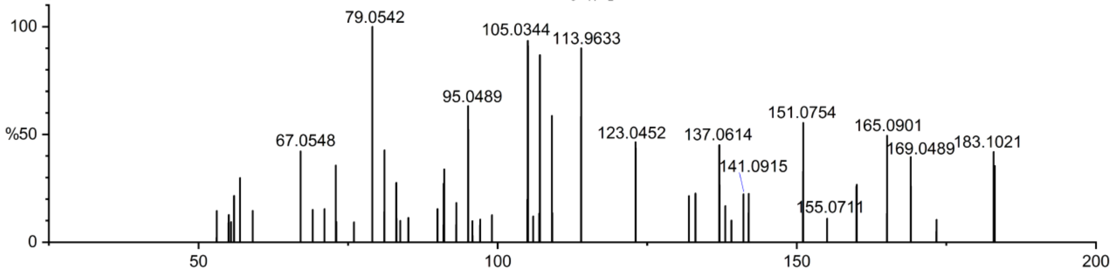
79.0542
105.0344
113.9633
95.0489
67.0548
123.0452
137.0614
151.0754
165.0901
169.0489
183.1021
141.0915
155.0711
%50

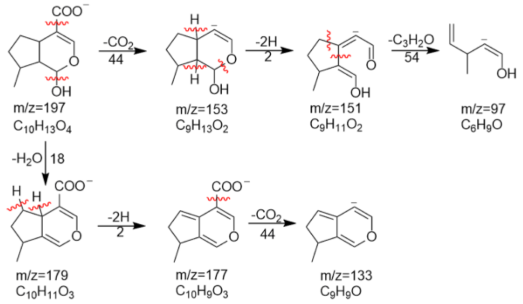
m/z=197
$C_{10}H_{13}O_4$
m/z=153
$C_9H_{13}O_2$
m/z=151
$C_9H_{11}O_2$
m/z=97
C_6H_9O
m/z=179
$C_{10}H_{11}O_3$
m/z=177
$C_{10}H_9O_3$
m/z=133
C_9H_9O

IP6

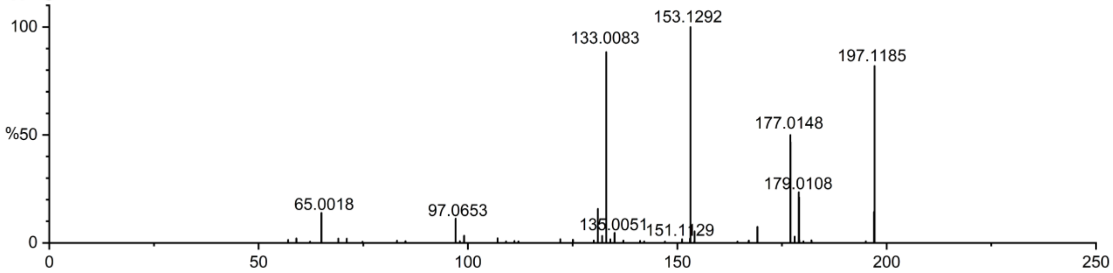
153.1292
133.0083
197.1185
177.0148
179.0108
65.0018
97.0653
135.0051
151.1129
%50

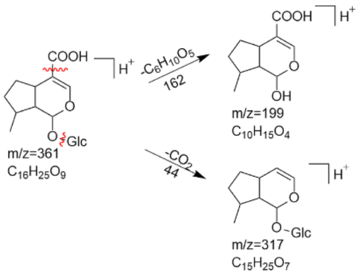
COOH
H+
$-C_6H_{10}O_5$
162
$-CO_2$
44
O Glc
m/z=361
$C_{16}H_{25}O_9$
OH
m/z=199
$C_{10}H_{15}O_4$
m/z=317
$C_{15}H_{25}O_7$

IP7

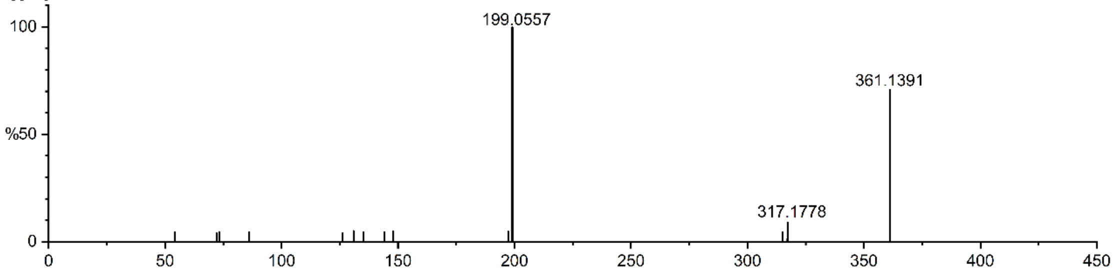
199.0557
361.1391
317.1778
100
%50
0
0
50
100
150
200
250
300
350
400
450

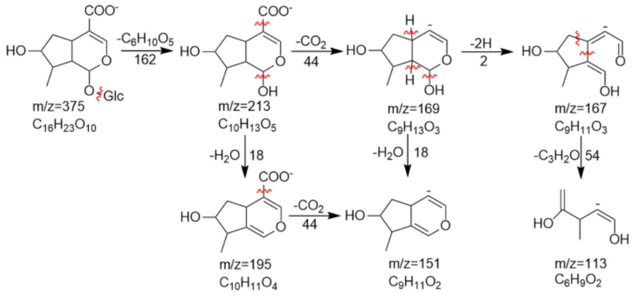
COO^-
HO
Glc
m/z=375
$C_{16}H_{23}O_{10}$
$-C_6H_{10}O_5$
162
m/z=213
$C_{10}H_{13}O_5$
$-CO_2$
44
m/z=169
$C_9H_{13}O_3$
-2H
2
m/z=167
$C_9H_{11}O_3$
$-H_2O$ 18
m/z=195
$C_{10}H_{11}O_4$
m/z=151
$C_9H_{11}O_2$
$-C_3H_2O$ 54
m/z=113
$C_6H_9O_2$

IP8

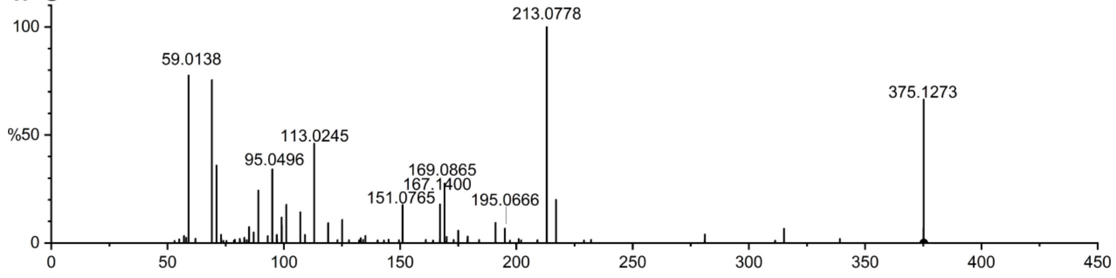
213.0778
59.0138
375.1273
113.0245
95.0496
169.0865
167.1400
151.0765
195.0666
100
%50
0
0
50
100
150
200
250
300
350
400
450

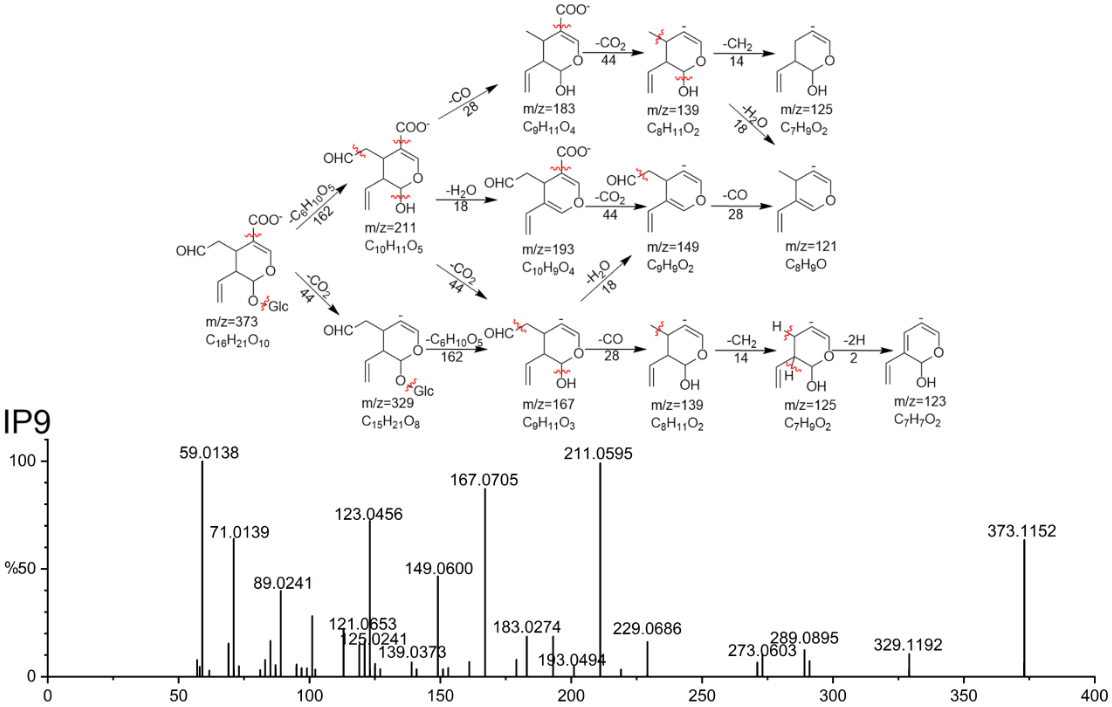
IP9
m/z=373
$C_{16}H_{21}O_{10}$
m/z=211
$C_{10}H_{11}O_5$
m/z=183
$C_9H_{11}O_4$
m/z=139
$C_8H_{11}O_2$
m/z=125
$C_7H_9O_2$
m/z=193
$C_{10}H_9O_4$
m/z=149
$C_9H_9O_2$
m/z=121
C_8H_9O
m/z=329
$C_{15}H_{21}O_8$
m/z=167
$C_9H_{11}O_3$
m/z=139
$C_8H_{11}O_2$
m/z=125
$C_7H_9O_2$
m/z=123
$C_7H_7O_2$
59.0138
71.0139
89.0241
121.0653
125.0241
123.0456
139.0373
149.0600
167.0705
183.0274
193.0494
211.0595
229.0686
273.0603
289.0895
329.1192
373.1152

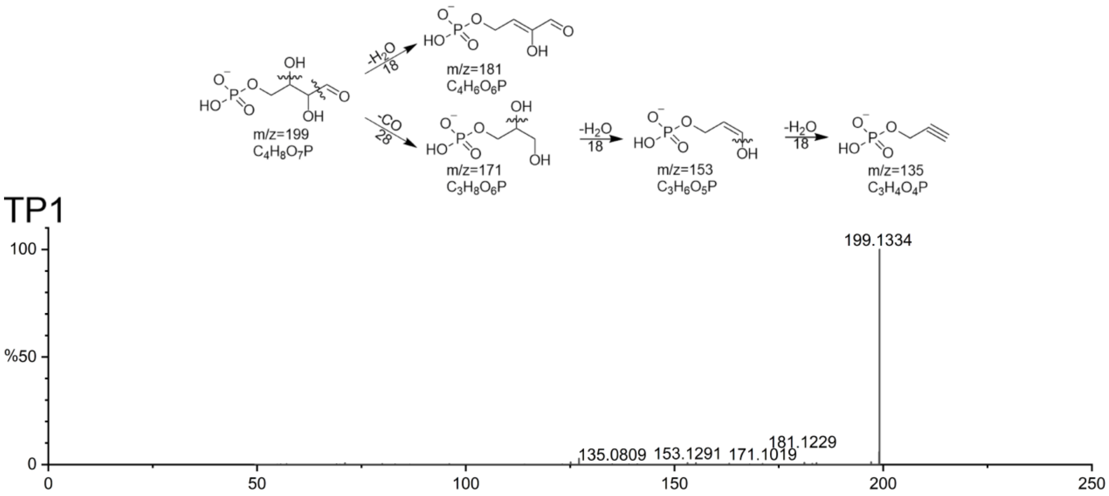
TP1
m/z=199
$C_4H_8O_7P$
m/z=181
$C_4H_6O_6P$
m/z=171
$C_3H_8O_6P$
m/z=153
$C_3H_6O_5P$
m/z=135
$C_3H_4O_4P$
199.1334
135.0809
153.1291
171.1019
181.1229

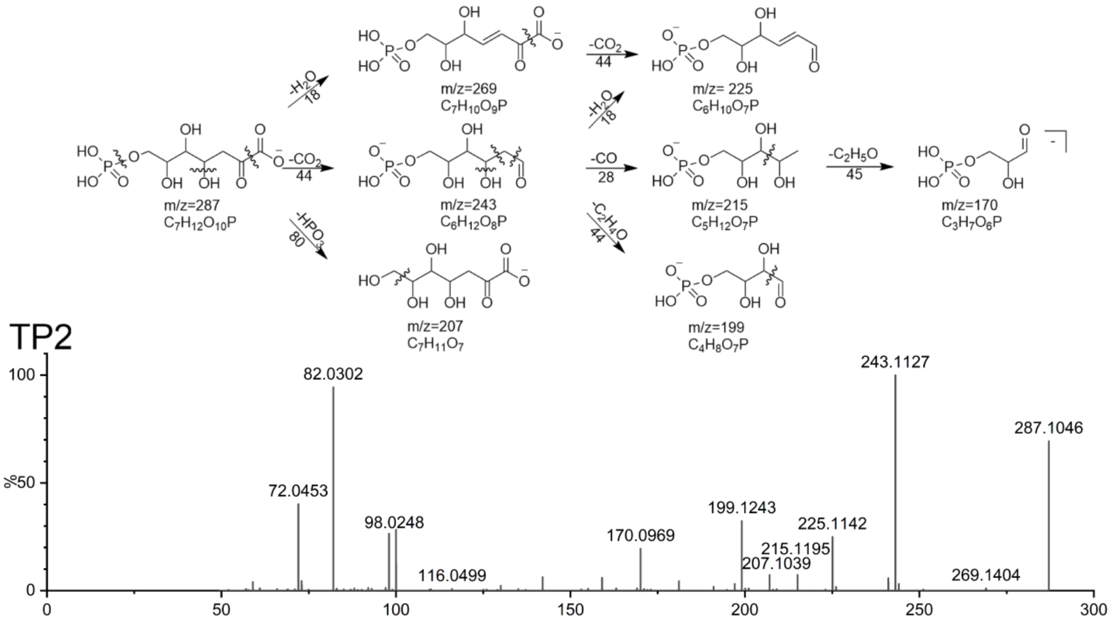

TP2
m/z=287
$C_7H_{12}O_{10}P$
$-CO_2$ 44
$-H_2O$ 18
$-HPO_3$ 80
m/z=269
$C_7H_{10}O_9P$
m/z=243
$C_6H_{12}O_8P$
m/z=207
$C_7H_{11}O_7$
-CO 28
$-C_2H_4O$ 44
m/z= 225
$C_6H_{10}O_7P$
m/z=215
$C_5H_{12}O_7P$
m/z=199
$C_4H_8O_7P$
$-C_2H_5O$ 45
m/z=170
$C_3H_7O_6P$
82.0302
72.0453
98.0248
116.0499
170.0969
199.1243
207.1039
215.1195
225.1142
243.1127
269.1404
287.1046
%

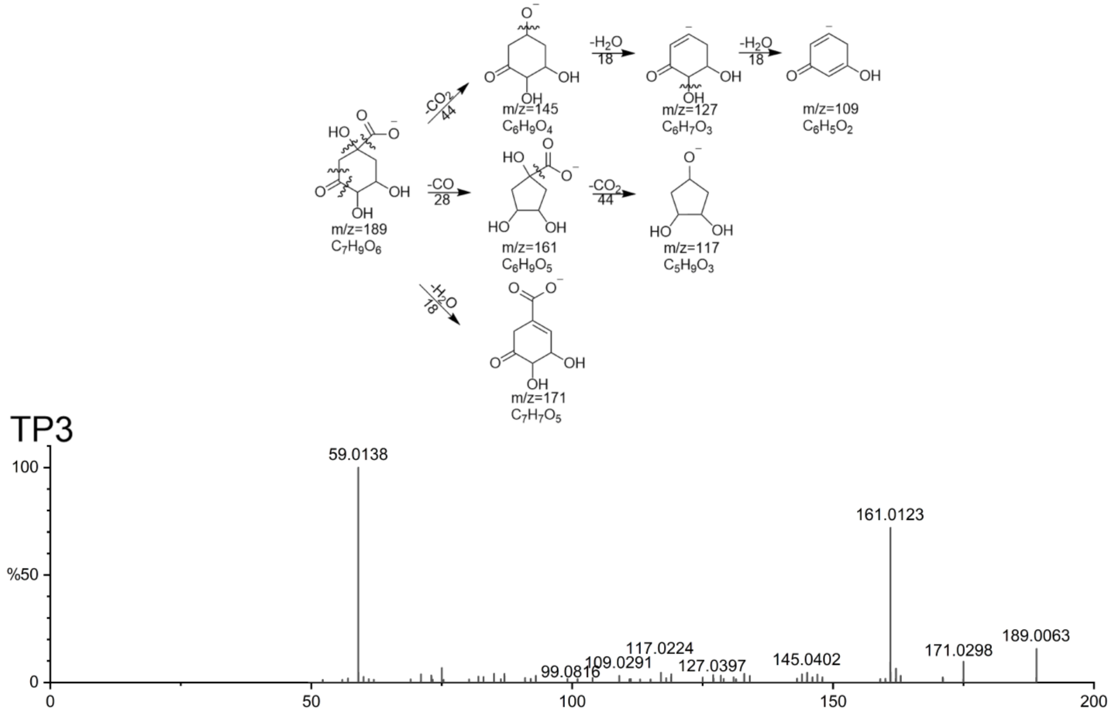

TP3
m/z=189
$C_7H_9O_6$
$-CO_2$ 44
-CO 28
$-H_2O$ 18
m/z=145
$C_6H_9O_4$
m/z=127
$C_6H_7O_3$
m/z=109
$C_6H_5O_2$
m/z=161
$C_6H_9O_5$
m/z=117
$C_5H_9O_3$
m/z=171
$C_7H_7O_5$
59.0138
99.0816
109.0291
117.0224
127.0397
145.0402
161.0123
171.0298
189.0063
%50

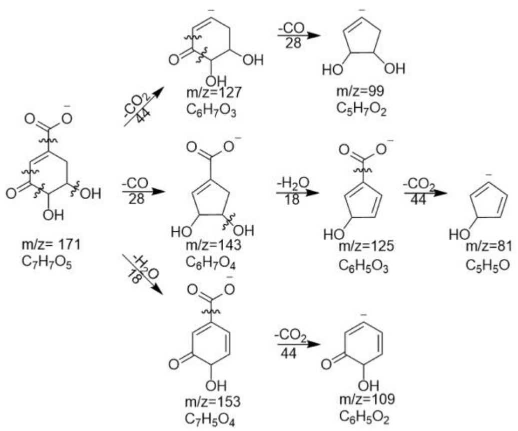
-CO
28
m/z=127
$C_6H_7O_3$
m/z=99
$C_5H_7O_2$
-CO₂ 44
m/z= 171
$C_7H_7O_5$
-CO
28
m/z=143
$C_6H_7O_4$
-H₂O
18
m/z=125
$C_6H_5O_3$
-CO₂
44
m/z=81
C_5H_5O
-H₂O
18
m/z=153
$C_7H_5O_4$
-CO₂
44
m/z=109
$C_6H_5O_2$

TP4

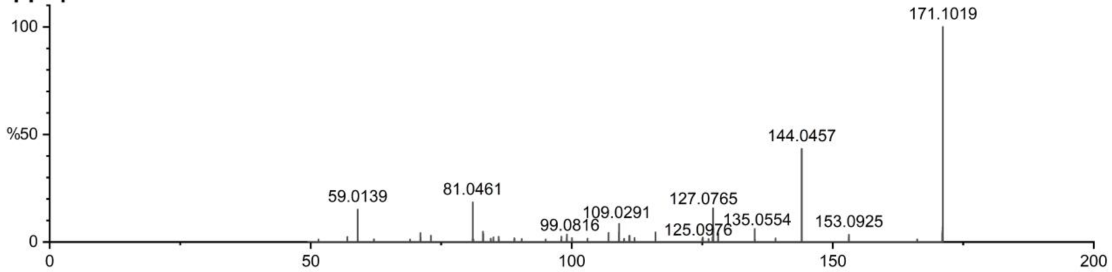
100
%50
0
171.1019
144.0457
59.0139
81.0461
127.0765
109.0291
99.0816
135.0554
153.0925
125.0976
0
50
100
150
200

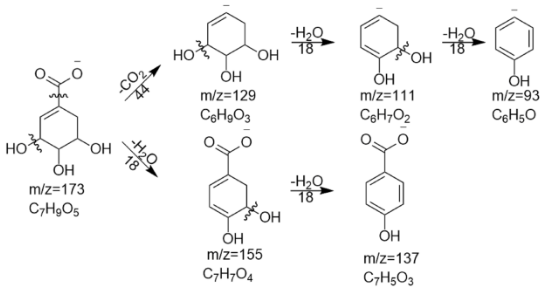
-CO₂ 44
m/z=129
$C_6H_9O_3$
-H₂O
18
m/z=111
$C_6H_7O_2$
-H₂O
18
m/z=93
C_6H_5O
m/z=173
$C_7H_9O_5$
-H₂O 18
m/z=155
$C_7H_7O_4$
-H₂O
18
m/z=137
$C_7H_5O_3$

TP5

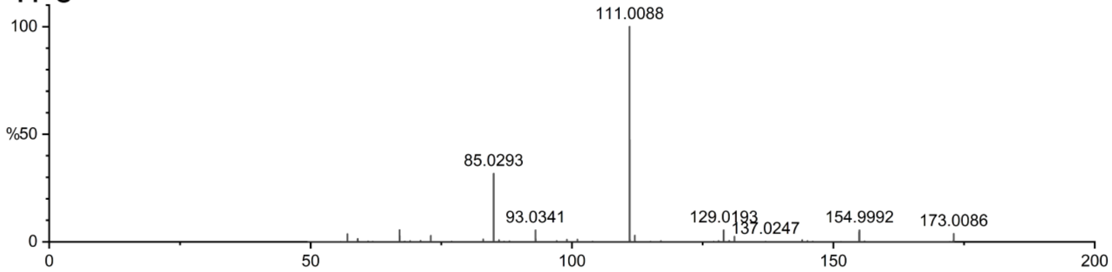
100
%50
0
111.0088
85.0293
93.0341
129.0193
137.0247
154.9992
173.0086
0
50
100
150
200

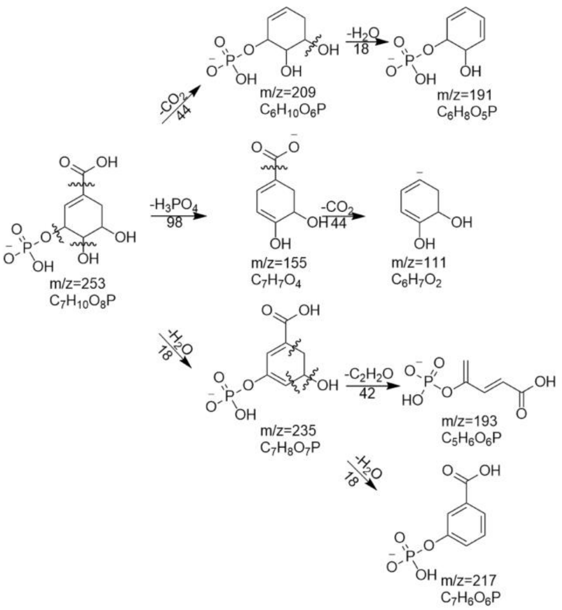
-CO2 44
m/z=209
$C_6H_{10}O_6P$
-H2O 18
m/z=191
$C_6H_8O_5P$
m/z=253
$C_7H_{10}O_8P$
-H3PO4 98
m/z=155
$C_7H_7O_4$
-CO2 44
m/z=111
$C_6H_7O_2$
-H2O 18
m/z=235
$C_7H_8O_7P$
-C2H2O 42
m/z=193
$C_5H_6O_6P$
-H2O 18
m/z=217
$C_7H_6O_6P$

TP6

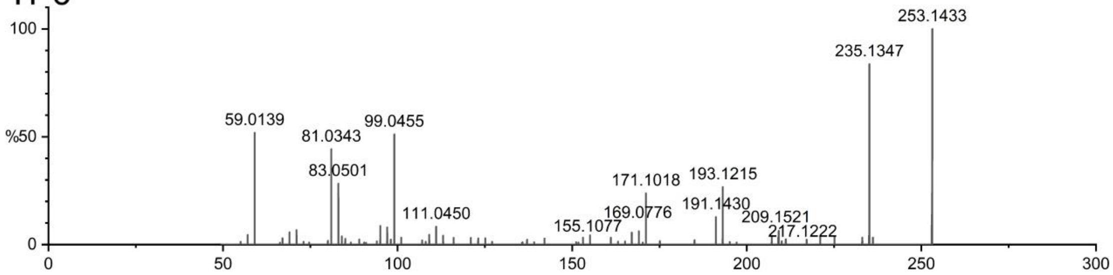
100
%50
0
253.1433
235.1347
59.0139
81.0343
99.0455
83.0501
111.0450
155.1077
169.0776
171.1018
191.1430
193.1215
209.1521
217.1222
0
50
100
150
200
250
300

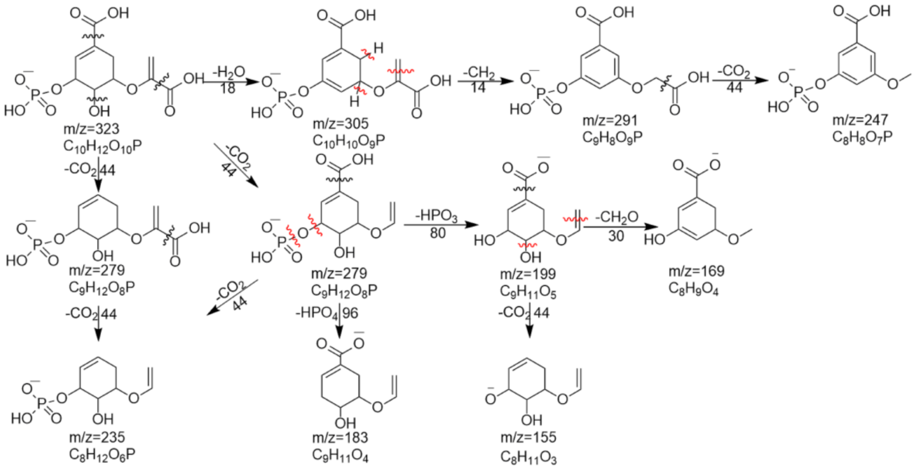
m/z=323
$C_{10}H_{12}O_{10}P$
-H2O 18
m/z=305
$C_{10}H_{10}O_9P$
-CH2 14
m/z=291
$C_9H_8O_9P$
-CO2 44
m/z=247
$C_8H_8O_7P$
-CO2 44
m/z=279
$C_9H_{12}O_8P$
-CO2 44
m/z=279
$C_9H_{12}O_8P$
-HPO3 80
m/z=199
$C_9H_{11}O_5$
-CH2O 30
m/z=169
$C_8H_9O_4$
-CO2 44
-CO2 44
-HPO4 96
-CO2 44
m/z=235
$C_8H_{12}O_6P$
m/z=183
$C_9H_{11}O_4$
m/z=155
$C_8H_{11}O_3$

TP7

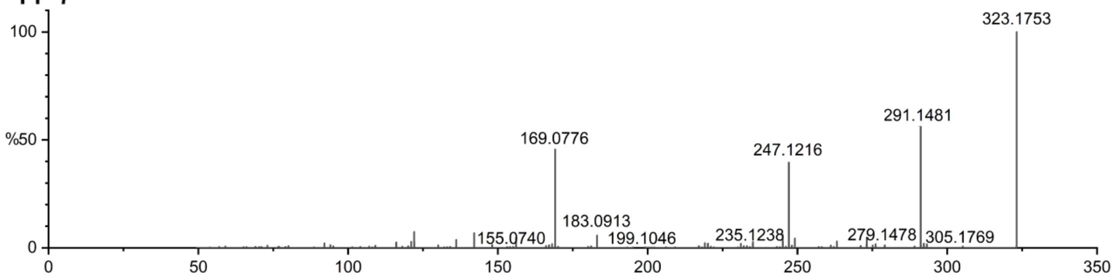
100
%50
0
323.1753
291.1481
169.0776
247.1216
183.0913
155.0740
199.1046
235.1238
279.1478
305.1769
0
50
100
150
200
250
300
350

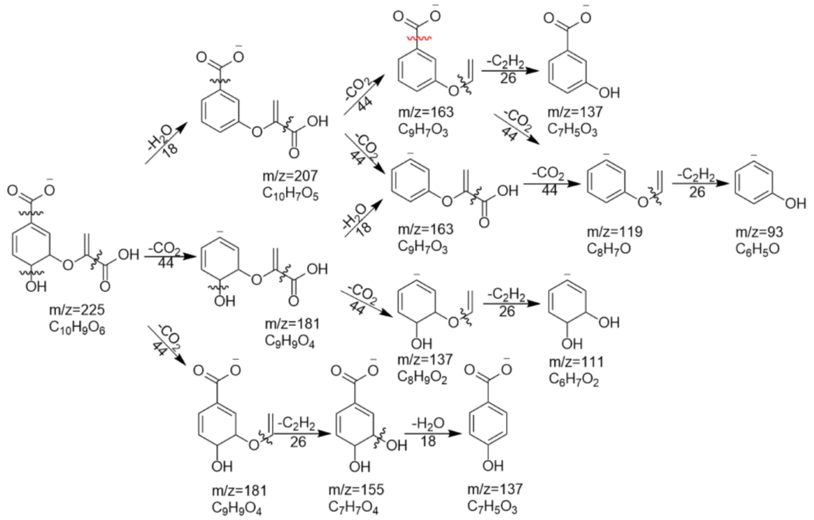

m/z=225
$C_{10}H_9O_6$
-CO_2 44
-H_2O 18
m/z=207
$C_{10}H_7O_5$
m/z=163
$C_9H_7O_3$
-C_2H_2 26
m/z=137
$C_7H_5O_3$
m/z=163
$C_9H_7O_3$
m/z=119
C_8H_7O
m/z=93
C_6H_5O
m/z=181
$C_9H_9O_4$
m/z=137
$C_8H_9O_2$
m/z=111
$C_6H_7O_2$
m/z=181
$C_9H_9O_4$
m/z=155
$C_7H_7O_4$
m/z=137
$C_7H_5O_3$

TP8

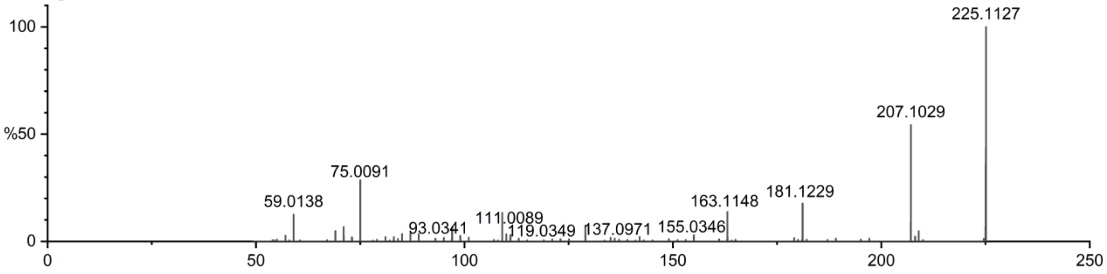

225.1127
207.1029
75.0091
59.0138
181.1229
163.1148
111.0089
93.0341
119.0349
137.0971
155.0346
100
%50
0
0
50
100
150
200
250

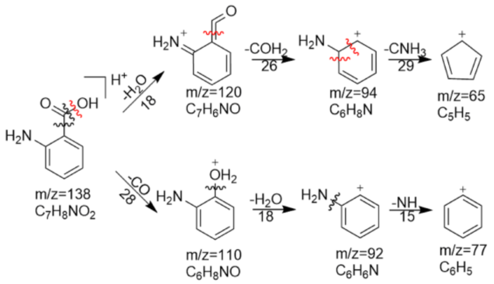

m/z=138
$C_7H_8NO_2$
-H_2O 18
m/z=120
C_7H_6NO
-COH_2 26
m/z=94
C_6H_8N
-CNH_3 29
m/z=65
C_5H_5
-CO 28
m/z=110
C_6H_8NO
-H_2O 18
m/z=92
C_6H_6N
-NH 15
m/z=77
C_6H_5

TP9

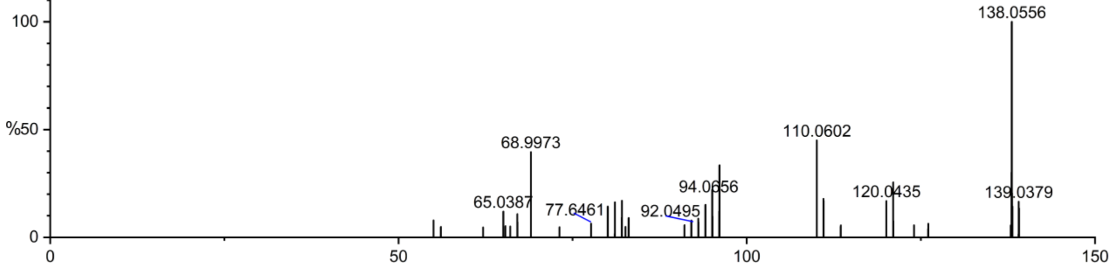

138.0556
110.0602
68.9973
94.0656
120.0435
139.0379
65.0387
77.6461
92.0495
100
%50
0
0
50
100
150

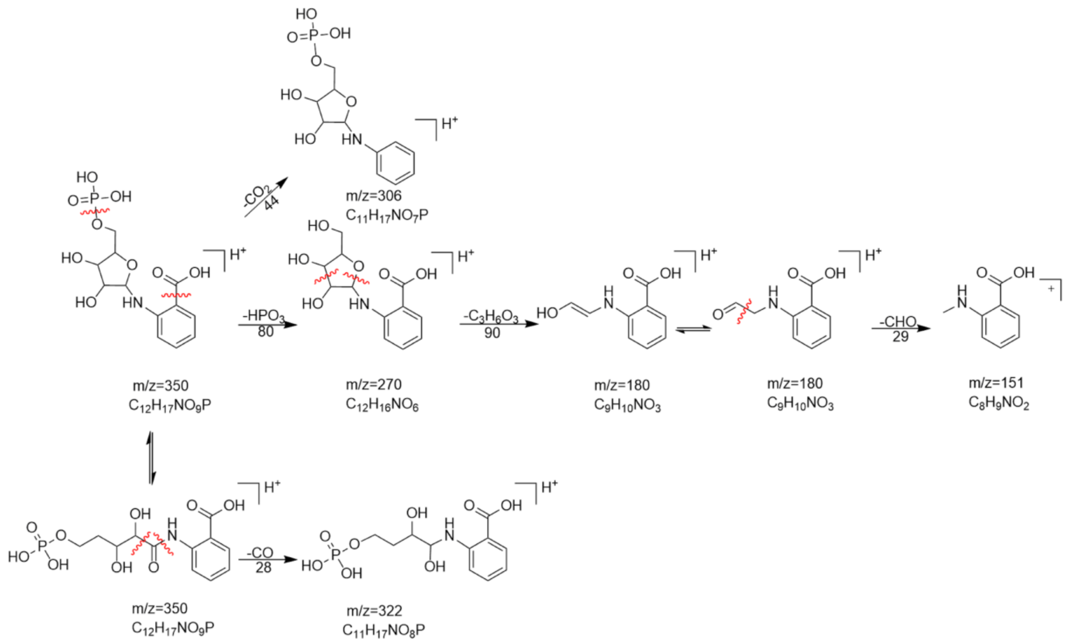
-CO2
44
-HPO3
80
-C3H6O3
90
-CHO
29
-CO
28
m/z=350
C12H17NO9P
m/z=306
C11H17NO7P
m/z=270
C12H16NO6
m/z=180
C9H10NO3
m/z=180
C9H10NO3
m/z=151
C8H9NO2
m/z=350
C12H17NO9P
m/z=322
C11H17NO8P

TP10/TP11

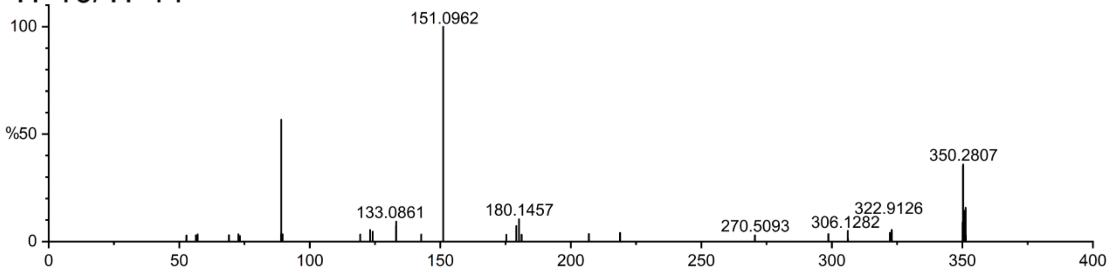
151.0962
350.2807
133.0861
180.1457
270.5093
306.1282
322.9126
%50
100
0
50
150
200
250
300
350
400

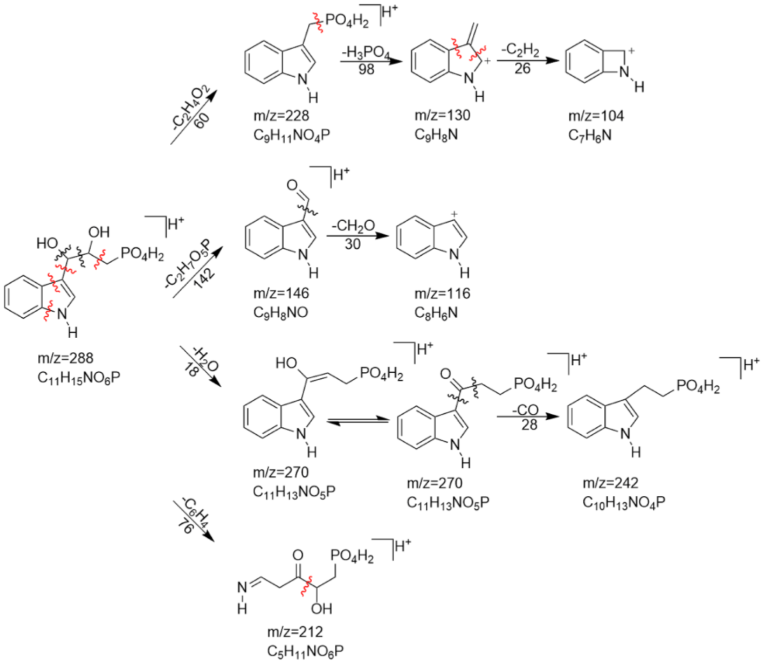
m/z=288
$C_{11}H_{15}NO_6P$
$-C_2H_4O_2$ 60
m/z=228
$C_9H_{11}NO_4P$
$-H_3PO_4$ 98
m/z=130
C_9H_8N
$-C_2H_2$ 26
m/z=104
C_7H_6N
$-C_2H_7O_5P$ 142
m/z=146
C_9H_8NO
$-CH_2O$ 30
m/z=116
C_8H_6N
$-H_2O$ 18
m/z=270
$C_{11}H_{13}NO_5P$
m/z=270
$C_{11}H_{13}NO_5P$
-CO 28
m/z=242
$C_{10}H_{13}NO_4P$
$-C_6H_4$ 76
m/z=212
$C_5H_{11}NO_6P$

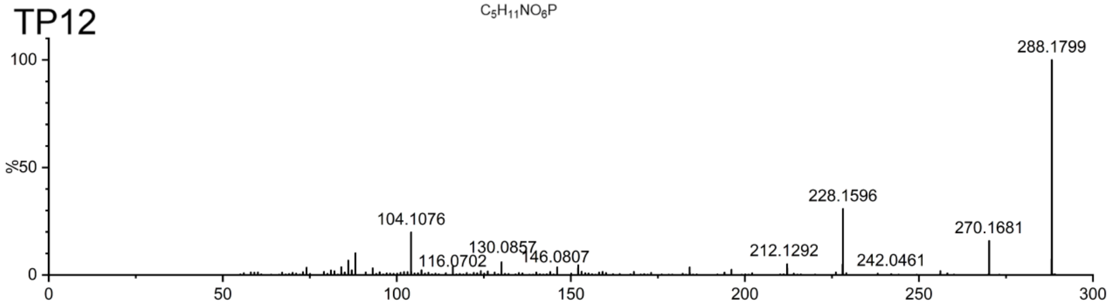
TP12
288.1799
228.1596
270.1681
104.1076
130.0857
116.0702
146.0807
212.1292
242.0461
%
100
50
0
0
50
100
150
200
250
300

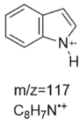
m/z=117
$C_8H_7N^{•+}$

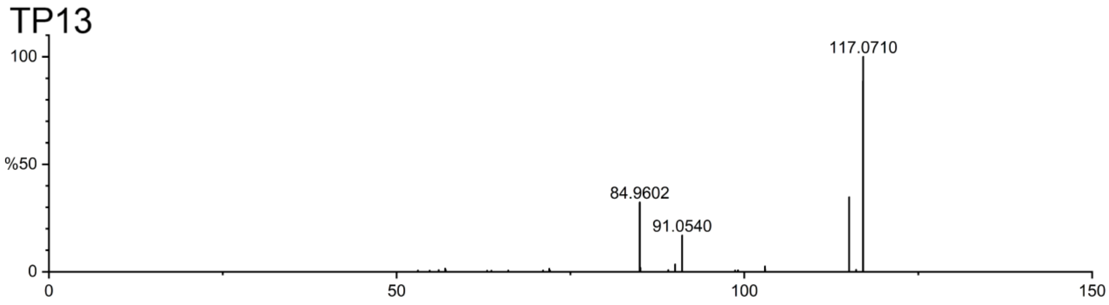
TP13
117.0710
84.9602
91.0540
%
100
50
0
0
50
100
150

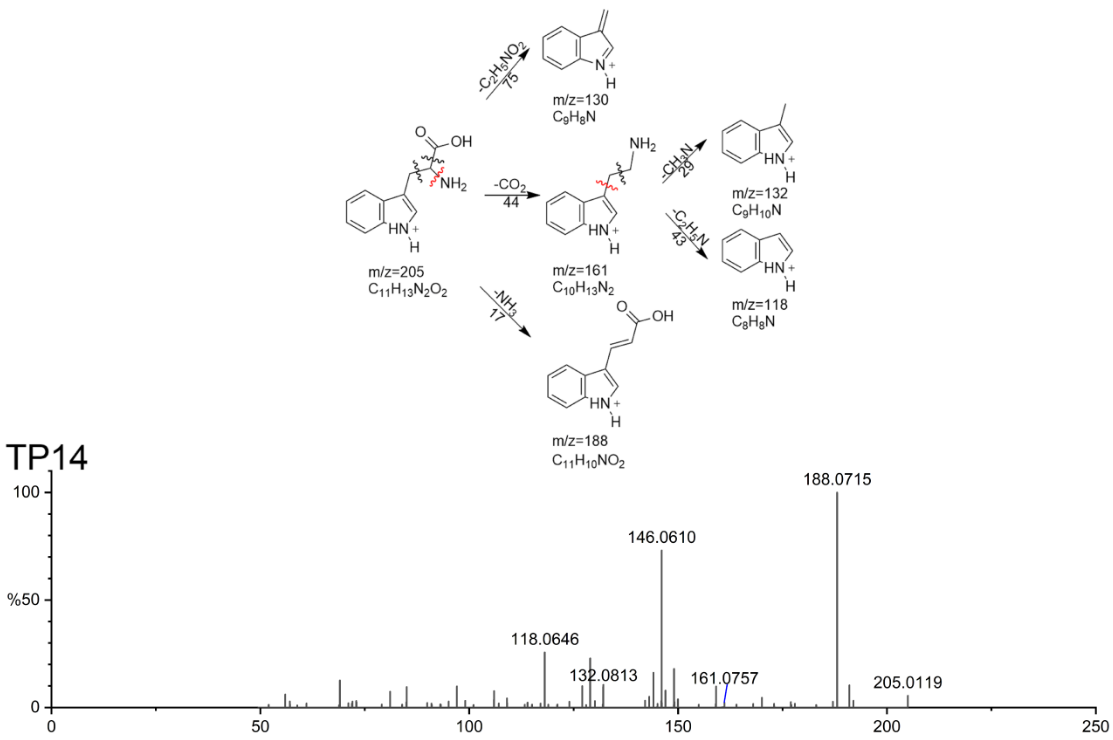

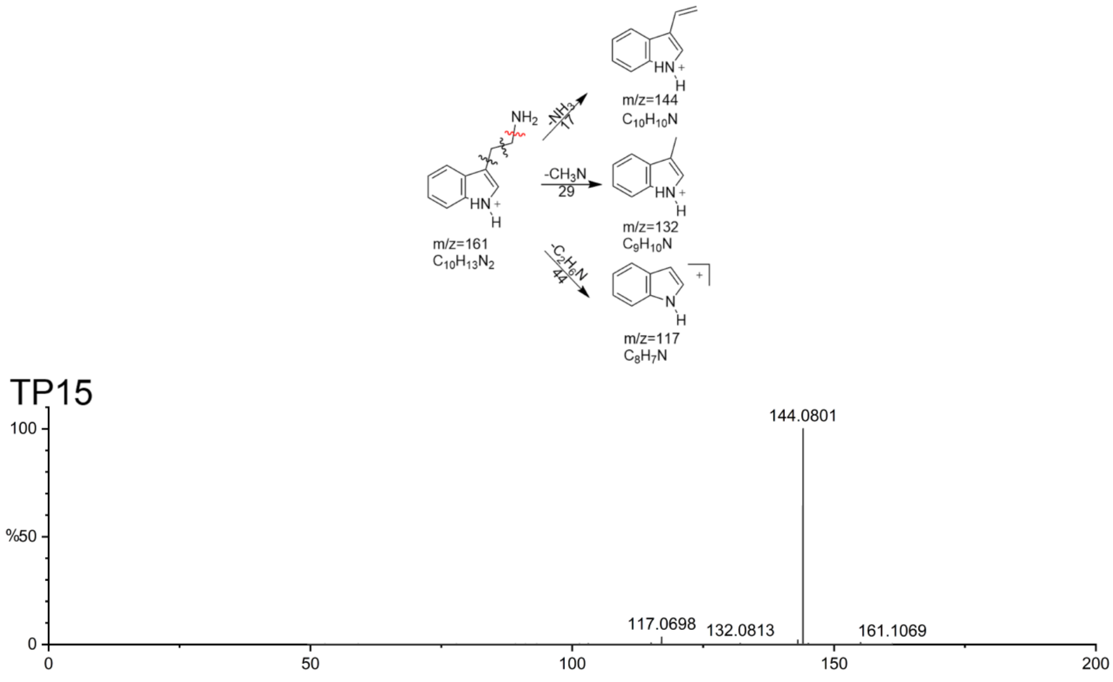

Supplementary Figure 4. Fragmentation pathways for the camptothecin (CPT) analogs and biosynthetic precursors.

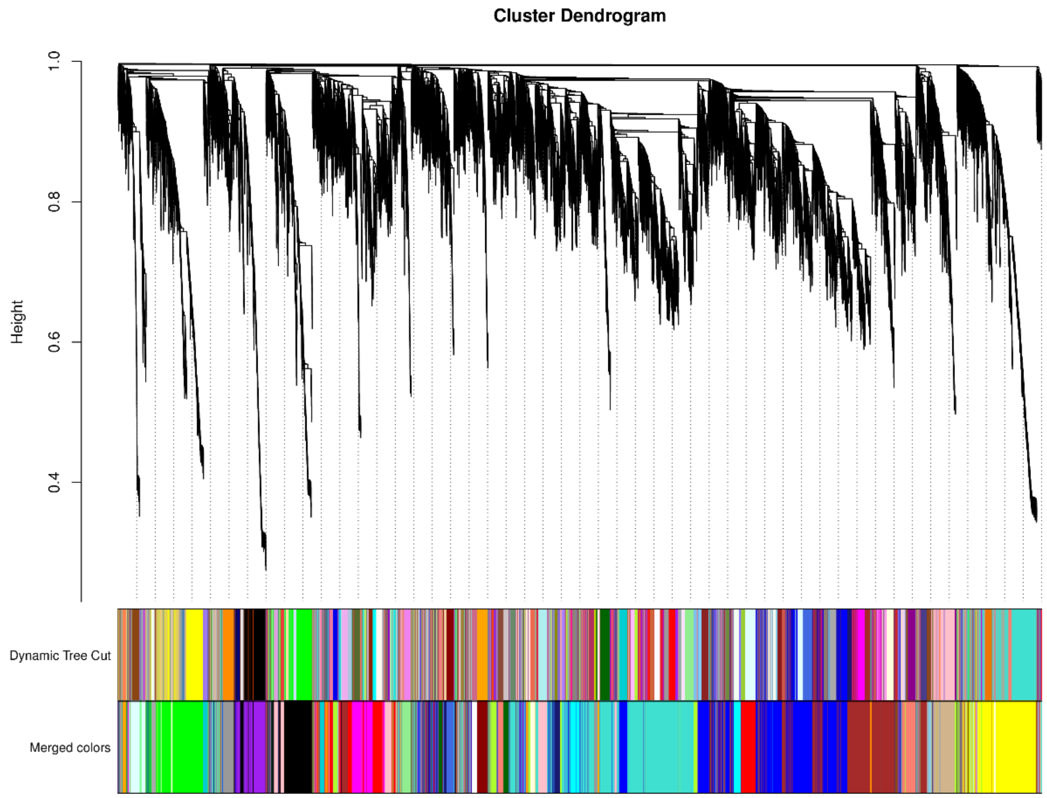

Supplementary Figure 5. Dendrogram of gene clusters with the assigned merged module colors and the original module colors. Different colors under the dendrogram indicate co-expression modules identified using weighted correlation network analysis.

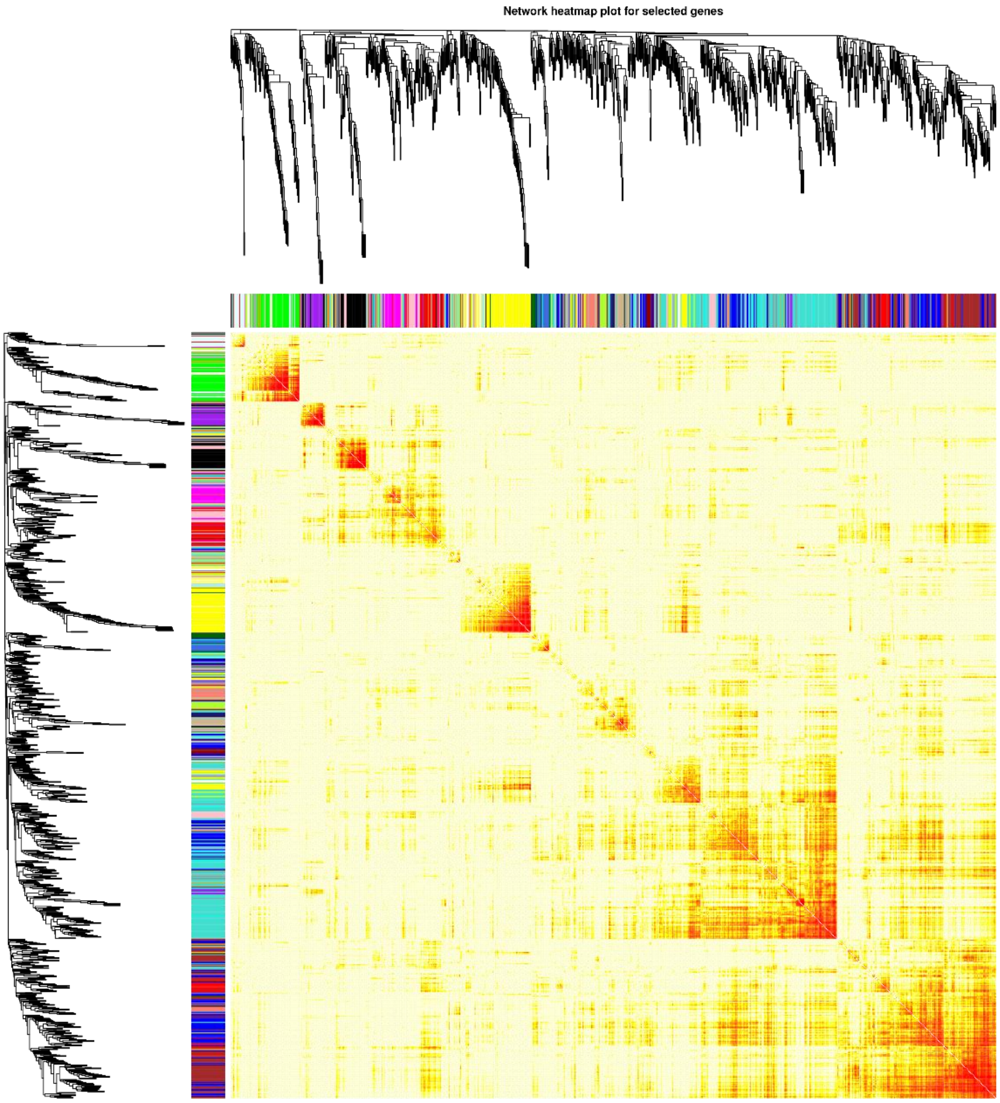

Supplementary Figure 6. Network heatmap for selected genes. The left side and the top represent the gene dendrogram and modules. The color bar next to the dendrogram indicates the co-expressed modules.
