# Supplementary material for: Integrative Analysis of Elicitor-Induced Camptothecin Biosynthesis in Camptotheca acuminata Plantlets Through a Combined Omics Approach
Source: Front Plant Sci. 2022 Mar 24;13:851077. doi: 10.3389/fpls.2022.851077 (PMC8987726; doi:10.3389/fpls.2022.851077)
Supplement: Supplementary file 2 [file Table_1.DOCX]

Supplementary Material

**Supplementary Table 1**. The primers used in qRT-PCR verification

| **Primer name** | **Primer sequence (5’→3’)** |
| --- | --- |
| Ca-18S-F | GGTGGTGACGGGTGACG |
| Ca-18S-R | GTCAGGATTGGGTAATTTGCG |
| Ca10OMT-F | GGGACAGAAAACTTGGACTT |
| Ca10OMT-R | ATCTGCTTTGACCTTCTCCA |
| CaTSB-F | GCACGGATGAAGAGGCATTG |
| CaTSB-R | AGCACGACCTTGGTTCCATT |
| CaTDC1-F | TTCGAGATCCTTGTGCCTCG |
| CaTDC1-R | TGGGTCATGTAAACTCGCCC |
| CaTDC2-F | AGGCCAAGTGTGTTGGTGAA |
| CaTDC2-R | TACAAACCACAGTCGGAGCG |
| CaDXR-F | CACTGTTCAACTCAGCCACC |
| CaDXR-R | CTCCAGCAATTAAAGTCTCCTT |
| CaIPI1-F | CGGATCCATGTCGGCAGCCTCACATATA |
| CaIPI1-R | CTCTAGATTATGTCAACTTGTGAATGGTTTTC |
| CaIPI2-F | AACCCTGATGAAGTCGCCG |
| CaIPI2-R | TGGTCCCACCACTTGAACAAA |
| CaGPPS-F | ACAGGCGAGACCATGCAAAT |
| CaGPPS-R | GCAGTTTGCCCAGCAAGAAG |
| CaCPR1-F | TGAAACTGGTGCACTCTCTGAG |
| CaCPR1-R | CAGTCTTGGAGCTGTCTAGAGATC |
| CYP72A610-F | TTTGTTTGATCTTTAATTTGCTGCTG |
| CYP72A610-R | ACCACAACTTTATTAATCAAGGAGAGAAC |
| CYP72A565-F | GCCTATTAATTTGTTATGTCCCCTAGC |
| CYP72A565-R | AGAAGGATGAAGGGTAACAATGCTAAC |
